# Decoding adipose–brain crosstalk: Distinct lipid cargo in human adipose‐derived extracellular vesicles modulates amyloid aggregation in Alzheimer's disease

**DOI:** 10.1002/alz.70603

**Published:** 2025-10-02

**Authors:** Li Yang, Michael Chan, Jianting Sheng, Shaohua Qi, Bill Chan, Dharti Shantaram, Xilal Y. Rima, Eduardo Reategui, Xianlin Han, Willa A. Hsueh, Stephen T. C. Wong

**Affiliations:** ^1^ Systems Medicine and Bioengineering Department Houston Methodist Neal Cancer Center Houston Methodist Hospital Houston Texas USA; ^2^ Ting Tsung & Wei Fong Chao Center for BRAIN Houston Methodist Academic Institute and Weill Cornell Medicine Houston Texas USA; ^3^ Diabetes and Metabolism Research Center Division of Endocrinology Diabetes & Metabolism Department of Internal Medicine The Ohio State University Wexner Medical Center Columbus Ohio USA; ^4^ Barshop Institute for Longevity and Aging Studies and Department of Medicine University of Texas Health Science Center at San Antonio San Antonio Texas USA; ^5^ Departments of Medicine Radiology, Neurology Pathology and Laboratory Medicine Houston Methodist Hospital and Weill Cornell Medicine Houston Texas USA

**Keywords:** adipocyte‐derived extracellular vesicles, Alzheimer's disease, Aβ fibrillization, exosome‐enriched extracellular vesicles, lipid homeostasis, lipidomics, obesity

## Abstract

**INTRODUCTION:**

Obesity is a major modifiable risk factor for Alzheimer's disease (AD), but the mechanistic link between peripheral metabolic dysfunction and AD progression remains unclear. Adipose‐derived extracellular vesicles (EVs) may penetrate the brain and alter lipid homeostasis, contributing to neurodegeneration.

**METHODS:**

We isolated exosome‐enriched EVs from subcutaneous and visceral fat of lean and obese individuals, followed by lipidomic profiling. An in vitro amyloid‐β (Aβ) aggregation assay using purified Aβ40 and Aβ42 peptides was performed under lipid environments mimicking physiological and pathological states.

**RESULTS:**

Obese‐derived EVs exhibited distinct lipid profiles, particularly in lysophosphatidylcholine (LPC) and sphingomyelin (SM) species. Functional assays demonstrated that lipid identity and concentration critically influenced Aβ aggregation kinetics.

**DISCUSSION:**

Our study reveals that obesity‐associated EV lipids modulate Aβ aggregation, linking adipose metabolism to AD pathology. These findings support lipid‐targeted strategies as potential therapeutics for neurodegenerative diseases.

**Highlights:**

- Human adipose‐derived extracellular vesicles (EVs) from obese individuals exhibit distinct lipidomic profiles.
- EV lipids modulate amyloid‐β (Aβ) 40 and Aβ42 aggregation in a lipid‐type‐ and concentration‐dependent manner.
- Lysophosphatidylcholine (LPC) and sphingomyelin (SM) species from obese EVs significantly deregulate Aβ fibrillization in vitro.
- EV lipid cargo links peripheral metabolic state to amyloid pathology in Alzheimer's disease.

## 1 BACKGROUND

Alzheimer's disease (AD) and its associated dementia are projected to affect about 82 million people worldwide by 2050.^1^ The lipid‐rich brain is influenced by lipid‐related genes and peripheral lipid abnormalities, particularly in obesity, which has been identified as a top modifiable risk factor for dementia in epidemiological studies.^2^ Mechanisms linking obesity to AD pathology include lipotoxicity, insulin resistance, adipokine signaling, inflammation, and immune cell fate shifts;^3^, ^4^, ^5^, ^6^ all of which are exacerbated in individuals with obesity and primarily contribute by the peripheral tissues. However, the molecular mechanisms connecting specific tissues, such as adipose tissue in obesity, to AD pathology remain largely unclear.

Clinical lipidomic and metabolomic studies consistently reveal early‐stage dysregulation in various lipid classes in AD brains, including ceramides, sphingomyelin, cholesterol, and glycerolipids.^7^ Lipid metabolism influences multiple pathogenic processes in AD, including amyloidosis, tauopathy, neuroinflammation, neuronal damage, energy deficits, oxidative stress, and myelin homeostasis.^8^, ^9^

The amyloid cascade hypothesis proposes that amyloid‐β (Aβ) 40 and 42 peptides are generated via the amyloidogenic pathway, mediated by the sequential cleavage of β‐secretase and γ‐secretase, and subsequently secreted into the extracellular space.^10^, ^11^, ^12^ Under pathophysiological conditions, Aβ40 and Aβ42 aggregate into oligomers or assemble into symmetrical, periodic fibrils, a process known as Aβ fibrillization, which ultimately leads to the formation of amyloid plaques, a hallmark pathological feature that is visibly detectable in the brains of AD patients.^13^, ^14^, ^15^

Aβ fibrillization is influenced by multiple genetic and environmental factors, including gene regulation, Aβ polymorphism,^16^ metal ions,^17^ and lipids.^18^, ^19^, ^20^ Adipocyte‐derived extracellular vesicles (EVs) can cross the blood–brain barrier and deliver exogenous molecules, including RNAs, DNAs, proteins, and lipids, thereby disrupting the brain microenvironment.^21^, ^22^, ^23^, ^24^, ^25^, ^26^, ^27^, ^28^, ^29^ Although EV‐associated miRNAs have been implicated in AD progression,^21^, ^30^ the role of EV‐derived lipids in exacerbating AD pathology remains largely unresolved.

Lipids constitute the majority of the brain's dry mass and are crucial for both normal function and pathology.^31^ Cryo‐electron microscopy analysis of amyloid plaques has identified a substantial presence of Aβ fibrils alongside an enriched lipid content.^32^ The in vivo and in vitro study demonstrated that, lipid‐driven condensation of Alzheimer's Aβ peptide initiates its transition into amyloid aggregates.^33^ Research on the interaction between lipids and Aβ aggregation indicates that negatively charged phospholipids facilitate Aβ fibrillization, whereas neutral lipids exhibit minimal or no impact.^34^, ^35^ Moreover, lipid membranes with distinct compositions have been found to enhance Aβ fibrillization and aggregation on biological surfaces, including cell and vesicle membranes.^36^, ^37^ Building upon these findings, investigating the influence of lipid components on Aβ fibrillization under pathophysiological conditions from a lipid‐type‐specific perspective may provide valuable insights into lipid‐based therapeutic strategies for AD.^8^, ^31^

In this study, we first isolated pure, well‐characterized samples of EVs originating from an obese/lean population and conducted quality control (QC) assessments to confirm EV purity, size distribution, and morphology. We then systematically profiled and quantified the full range of lipids detected in the isolated EVs using multidimensional mass spectrometry and advanced lipidomics analysis to annotate, quantify, and identify lipid species with pathophysiological relevance. Subsequently, we investigate how specific EV‐derived lipids influence the aggregation dynamics of human Aβ peptides to reveal new insights into the intersection of metabolic dysfunction and neurodegenerative risk.

## 2 METHODS

### 2.1 Human subjects and adipose tissue collection

Subcutaneous (SQA) and visceral (VA) adipose tissues were collected from lean and obese individuals (Supporting Information Table 1) undergoing elective abdominal surgery at The Ohio State University Wexner Medical Center. Subjects were excluded if they had active infection or febrile illness, a history of cancer or organ transplantation, chronic use of immunosuppressive or anti‐inflammatory medications, or recent chemotherapy within the past year. Additional exclusion criteria included uncontrolled metabolic conditions (such as type 1 diabetes), autoimmune disorders, human immunodeficiency virus/acquired immunodeficiency syndrome (HIV/AIDS), substantial weight fluctuation (>10% change within the previous 3 months), or prior diagnosis of lipodystrophy, hemochromatosis, or other systemic illnesses likely to impact adipose metabolism. Tobacco use and hormonal treatments (e.g., corticosteroids, estrogen replacement) were also considered grounds for exclusion. Baseline clinical parameters—including body weight, body mass index (BMI), waist circumference, blood pressure, fasting glucose, insulin, triglycerides, and high‐density lipoprotein (HDL)—were recorded preoperatively.

Adipose tissue samples were harvested intraoperatively at the surgical site near the umbilicus. Abdominal subcutaneous adipose tissue was obtained via a 3–4 cm skin incision (approximately 1 cm in depth), followed by aspiration with a small liposuction cannula under sterile conditions and local anesthesia (1% lidocaine with 1:100,000 epinephrine). All procedures were performed by experienced surgical collaborators at The Ohio State University (OSU) clinical research center. Between 5 and10 grams of tissue were collected from each participant and processed within 60 min of excision.

Upon collection, samples were divided for two distinct applications. One portion was immediately flash‐frozen in liquid nitrogen and stored at −80°C for lipidomic analysis. The remaining tissue was transferred into ice‐cold sterile saline and promptly transported to the laboratory for adipocyte isolation and subsequent EV extraction.

To maintain sterility throughout processing, samples were placed in sterile containers filled with prechilled sterile media and handled exclusively under a laminar flow hood. Adipocytes and the stromal vascular fraction (SVF) were isolated by collagenase I digestion of finely minced adipose tissue, following previously established protocols.^38^

**RESEARCH IN CONTEXT**

1. **Systematic review**: We searched PubMed and Google Scholar using the terms “adipose‐derived EVs,” “lipidomics,” and “Aβ aggregation.” Although prior studies have suggested a link between metabolic dysfunction and Alzheimer's disease (AD), the specific role of human adipose‐derived extracellular vesicles (EV) lipids in modulating amyloid‐β (Aβ) pathology remains poorly defined.
2. **Interpretation**: Our study shows that EVs from obese adipose tissue carry specific lipid species that modulate Aβ40 and Aβ42 aggregation in a lipid‐type‐ and concentration‐dependent manner. These findings provide compelling molecular evidence linking peripheral lipid imbalance to Aβ aggregation, suggesting that metabolic dysfunction associated with obesity may contribute to central amyloid pathology via adipose‐derived EV lipids. Further in vivo validation is warranted to substantiate this proposed link.
3. **Future directions**: This work provides a foundation for investigating lipid homeostasis for AD, particularly in metabolically at‐risk populations. Future studies will focus on validating these lipid–Aβ interactions in vivo, identifying promising therapeutic strategies, and assessing their relevance to clinical outcomes.

### 2.2 Isolation of human adipocyte‐derived EVs

Exosome‐enriched EVs were isolated from human adipocytes for lipidomic analysis and in vivo studies. Human adipocytes were isolated from subcutaneous adipose tissue samples as described above. After isolation, adipocytes were washed and cultured in serum‐free maintenance medium (DMEM/F‐12 supplemented with 25 µg/mL insulin and 1% penicillin/streptomycin) at 37°C for 16–20 h in 6‐well plates (3 mL/well). This serum‐free condition was used to eliminate lipid contamination from fetal bovine serum (FBS) and ensure the purity of EVs for downstream applications.

After incubation, culture supernatants were collected and cleared of cellular debris by sequential low‐speed centrifugation. To improve EV yield and scalability, exosome‐enriched EVs were isolated using tangential flow filtration (TFF), followed by ultrafiltration to concentrate the preparations. The resulting EVs reached concentrations of approximately 10^12^ particles/mL.

All EV isolation and characterization procedures adhered to the guidelines of the International Society for Extracellular Vesicles (ISEV). Characterization of human adipocyte‐derived EVs was performed using microfluidic resistive pulse sensing (MRPS) to assess particle size and concentration, immunomagnetic bead capture for exosomal surface markers, and Western blotting for adipocyte‐specific and exosomal proteins.

### 2.3 In vivo visualization of EV distribution

For in vivo tracing in mice, EVs were isolated from mouse adipose tissue using a modified protocol incorporating collagenase II digestion. The purified EVs were labeled with the ExoGlow™‐Vivo EV Labeling Kit (Cat # EXOGV900A‐1) according to the manufacturer's instructions, and subsequently administered via tail vein injection at a dose of 6.55 × 10⁹ particles per mouse. Fluorescence imaging was performed using an IVIS (In Vivo Imaging System) to visualize EV biodistribution.

### 2.4 Lipidomics

Lipid species were analyzed using multidimensional mass spectrometry‐based shotgun lipidomics approach.^39^ In brief, each exosome sample was homogenized, and the protein content of each sample was determined by a Pierce BCA assay. An equivalent of 0.01 mg protein homogenate was then added to a glass tube along with a premixed lipid internal standard. Lipid extraction was performed using a modified Bligh and Dyer procedure.^40^ The lipid extract was dispersed in chloroform:methanol (1:1, v:v) at a ratio of 4000 µL/mg protein for storage.

For shotgun lipidomics, the lipid extract was further diluted to a total lipid concentration of ∼2 pmol/µL. The mass spectrometric analysis was performed on a triple quadrupole mass spectrometer (TSQ Altis, Thermo Fisher Scientific, San Jose, CA) and a hybrid quadrupole‐Orbitrap mass spectrometer (Q‐Exactive, Thermo Fisher Scientific, San Jose, CA), both equipped with an automated nanospray ion source device (TriVersa NanoMate, Advion Bioscience Ltd., Ithaca, NY) as described previously.^41^

**FIGURE 1:**
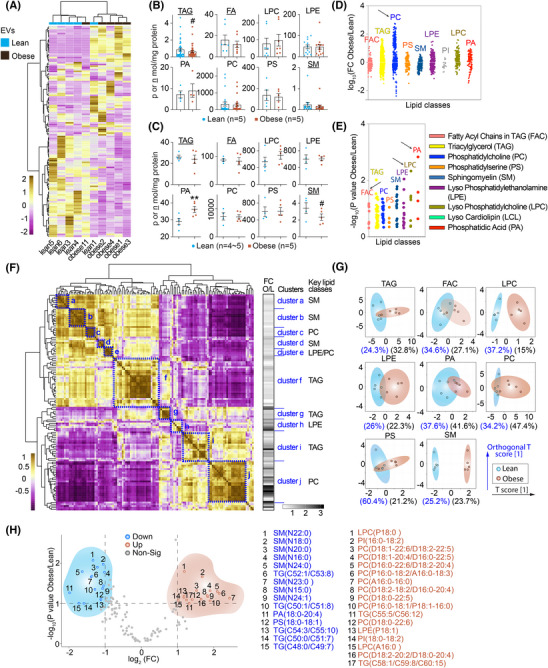
Lipidomic profiling of extracellular vesicles (EVs) from lean and obese individuals. (A) Unsupervised hierarchical clustering heatmap of lipidomic profiles from EVs isolated from lean and obese individuals. The analysis was performed using MetaboAnalyst 5.0 based on normalized lipid abundance values. Each column represents an individual sample (blue for lean, pink for obese), and each row corresponds to a lipid species. Color scale indicates Z‐score–normalized relative abundance, with blue representing lower levels and brown indicating higher levels. (B, C) Quantification of major lipid classes in EVs from lean and obese individuals. (B) Levels of specific lipid species—including triacylglycerol (TAG), free fatty acids (FA), lysophosphatidylcholine (LPC), lysophosphatidylethanolamine (LPE), phosphatidic acid (PA), phosphatidylcholine (PC), phosphatidylserine (PS), and sphingomyelin (SM)—were measured from EVs derived from pooled adipocyte isolates in each group (n = 5 per group). (C) Lipid class quantification in EVs from individual patients (*n* = 4‐5 per group), showing interindividual variation. Data are normalized to total EV protein (p or n mol/mg protein). Data are presented as mean ± SEM; two‐tailed t‐test; *, increase; ^#^, decrease; compared with the corresponding controls, *^/#^ *p *< 0.05, and **^/##^ *p *< 0.01. (D,E) Simplified Manhattan plots of lipid class alterations between obese and lean EVs (including Lean 1). (D) Fold changes (log₁₀[Obese/Lean]) of individual lipid classes stratified by lipid species. Each dot represents a detected lipid species, color‐coded by class. (E) Statistical significance (−log₁₀ P value) of lipid class differences calculated based on unpaired two‐tailed *t*‐tests using raw lipidomic concentrations from lean and obese individuals. Notable classes such as FAC, TAG, PC, LPC, and PA exhibit significant alterations in fold change and/or *p*‐value distribution. (F) Lipid–lipid correlation heatmap of EV lipidomic profiles from lean and obese individuals. Pairwise correlations between lipid species were calculated using MetaboAnalyst 5.0 based on combined datasets from both groups. The color scale represents the Pearson correlation coefficient, ranging from –0.5 (purple, negative correlation) to 1.0 (yellow, strong positive correlation). Hierarchical clustering was applied to group lipid species with similar covariation patterns, revealing distinct lipid modules and potential coregulatory networks. Ten major lipid clusters (a–j) were identified, each outlined and annotated with representative lipid classes on the right. Key lipid species contributing to each cluster are summarized and compared between obese and lean groups in the accompanying Supporting Information Tables 4 and 5. Fold‐change (obese/lean) values for each lipid species are also shown as a grayscale bar (FC O/L) adjacent to the cluster annotation, highlighting cluster‐specific lipid alterations associated with obesity. (G) Partial least squares discriminant analysis (PLS‐DA) plots showing lipid class–specific separation between EVs from lean and obese individuals (including Lean 1). EV lipidomic data were analyzed with individual patient samples as input. Samples from lean and obese subjects are shown in blue and brown, respectively, with ellipses representing 95% confidence intervals. The percentage of explained variance for the first and second components (T score [1] and orthogonal T score [1]) is indicated in parentheses. (H) Volcano plot identifying differentially abundant lipid species between obese and lean EVs. Each dot represents a lipid species, plotted by log_2_(fold change Obese/Lean) on the *x*‐axis and −log₁₀(P value) on the *y*‐axis. Blue dots indicate significantly downregulated species in obesity (*p*  <  0.05 and FC  <  −1), red dots represent significantly upregulated species (*p*  <  0.05 and FC  >  1), and grey dots are nonsignificant. Top 15 upregulated and 15 downregulated lipid species are annotated and listed by class and composition.

**FIGURE 2:**
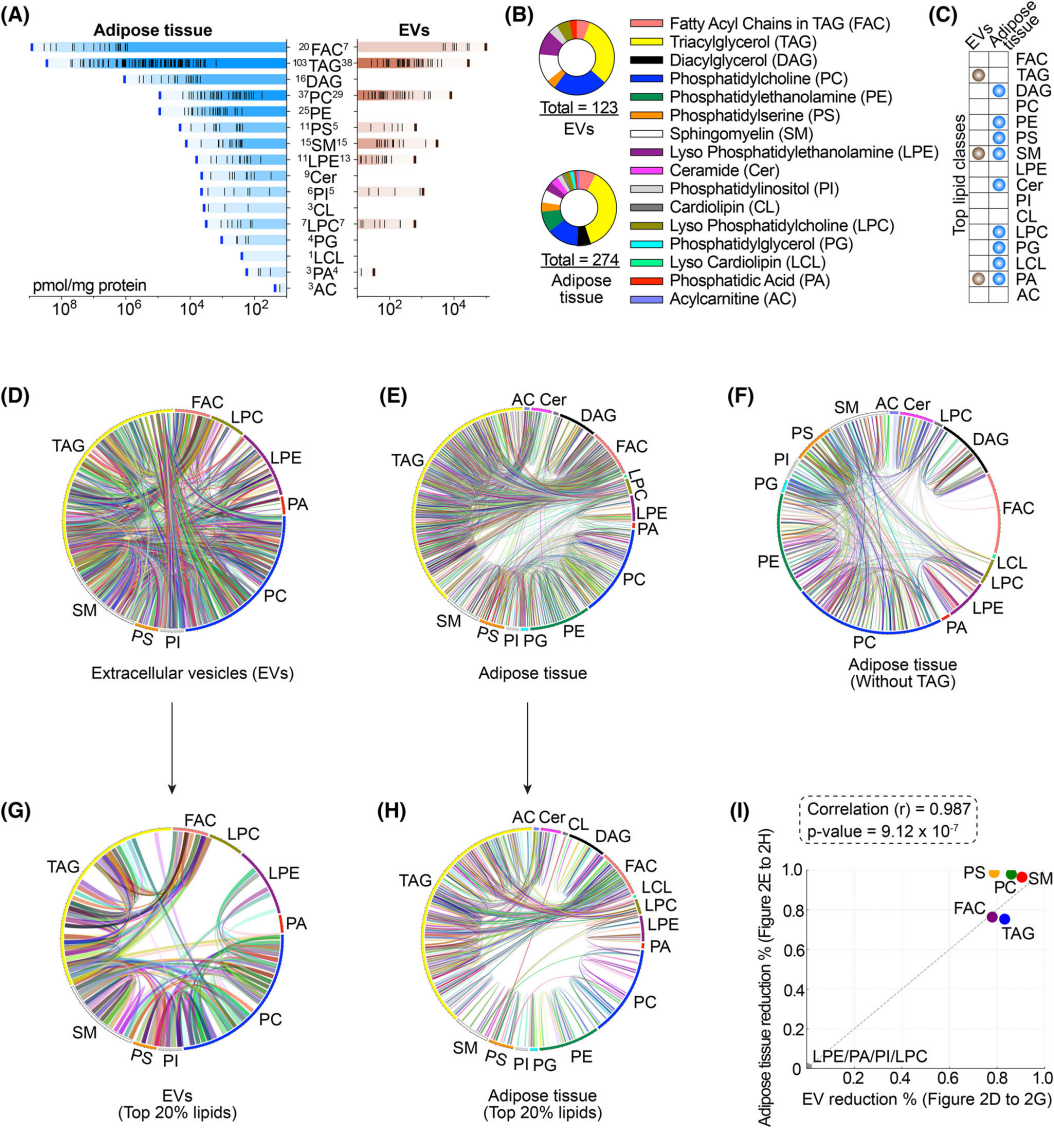
Lipid composition comparison between adipose tissue and extracellular vesicles (EVs). (A) Comparative analysis of lipid class abundance in adipose tissue and adipose‐derived EVs from lean and obese individuals. Lipid classes are ranked based on total abundance (pmol/mg protein) and displayed in descending order for each source. Horizontal bars represent the summed concentrations of all detected species within each lipid class, with tick marks indicating individual lipid species and bold tick mark indicating the total lipid class. Blue and brown bars denote adipose tissue and EVs, respectively. The numbers adjacent to each lipid class indicate the count of detected species. (B) Comparison of lipid species diversity across lipid classes in adipose‐derived EVs and matched adipose tissues. Donut charts show the distribution of identified lipid species grouped by class, with a total of 123 species detected in EVs and 274 in adipose tissue. Each color represents a distinct lipid class, as indicated in the legend. (C) Comparison of lipid class‐specific signatures between extracellular vesicles (EVs) and adipose tissue. The matrix highlights lipid classes that are enriched or selectively represented in either EVs (brown dots) or adipose tissue (blue dots). Signature lipid classes were defined based on relative abundance, distribution pattern, and specificity to each compartment. (D–H) Chord diagrams representing lipid coregulation networks among lipid species across different lipid classes in EVs and adipose tissue. (D) Full lipid network in EVs. (E) Full lipid network in adipose tissue. (F) Adipose tissue lipid network after excluding triacylglycerol (TAG) species to minimize skewing from high‐abundance lipids. (G) Network based on the top 20% most abundant lipid species in EVs. (H) Network based on the top 20% most abundant lipid species in adipose tissue. Each arc represents a lipid class, and connecting ribbons indicate pairwise coenrichment of lipid species between classes. The diagrams reveal distinct lipid interconnectivity patterns between EVs and adipose tissue, highlighting selective lipid packaging and modularity in EV lipid composition compared to the tissue of origin. (I) Correlation between lipid reduction in EVs and adipose tissue. Scatter plot showing the percentage reduction of major lipid classes in EVs (*x*‐axis; data from Figures 2D–G) versus corresponding reductions in adipose tissue (*y*‐axis; data from Figures 2E–H). Each dot represents a lipid class, labeled accordingly (e.g., SM, TAG, PC, etc.).

The data processing and analysis was performed based on the principles of shotgun lipidomics such as ion peak selection, baseline correction, isotope effect correction, etc.^39^, ^42^, ^43^ The final lipicomics result were normalized to the protein content (nmol/mg protein) (Supporting Information Table 2). Lipidomics profiling (Figures 1 and 2) was performed using MetaboAnalyst 5.0, following renormalization of the raw lipid data with the built‐in algorithm provided by the platform. Supporting Information Table 3 presents lipidomic data from adipose tissue.

### 2.5 Outlier detection analysis

To identify potential outlier samples from global lipidomic profiles, the Isolation Forest algorithm was applied using default hyperparameters. This unsupervised anomaly detection method is designed for high‐dimensional data and operates by randomly partitioning the feature space; samples that require fewer splits to be isolated are assigned higher anomaly scores. The algorithm is nonparametric, robust to noise, and does not assume a specific data distribution. To evaluate the influence of the identified outlier on downstream analyses, sensitivity analyses were performed by comparing results with and without the inclusion of outlier (Lean 1). This approach enabled validation of the robustness of major lipidomic findings and ensured that conclusions were not driven by a single data point.

### 2.6 Reagent preparation of ThT assay

Amyloid‐ β40 and 42, ultra pure, with NaOH, recombinant human (Sigma, #AG964 & AG970) were dissolved in 1% NH_4_OH to 1 mg/mL. And then add in 1XPBS to a stock concentration as 200 uM. Before thioflavin T (ThT) assay, the Aβs were sonicated for 200 s using a Marshall Scientific Sonifier model B450 (microtip, 10% amplitude) with intermittent pause to allow samples to cool down. ThT (Sigma, #T3516) was dissolved in PBS buffer and was filtered through a 0.2 µm syringe filter. Morin (Sigma, #M4008) and phenol Red (Sigma, #P3532) were also dissolved with PBS.

For the lipid details, sodium oleate (Sigma, #07501), sodium palmitate (Sigma, #P9767), Brain SM (Avanti, #860062P), egg SM (Avanti, #860061P), milk SM (Avanti, #860063P), egg  lysophosphatidylcholine (LPC) (Lyso PC; Avanti, #830071P), 18:0 LPC (Lyso PC; Avanti, #855775), 16:0‐20:4 phosphatidylethanolamine (PE) (Avanti, #850759C), 16:0‐18:1 PE (Avanti, #850757P), C18(Plasm)‐18:1 PE (Avanti, #852758P), and C18(Plasm)‐20:4 PE in ethanol (Cayman, #37137).

Stock solutions of sodium palmitate and sodium oleate (100 mM) were prepared by dissolving the fatty acid powders in ethanol, followed by brief sonication cycles (10 s per pulse, 200 W) on ice. Sonication was continued until the mixture became uniformly cloudy, indicating thorough dispersion. The stock solutions were protected from light and stored at 4°C. Prior to use, aliquots were prewarmed to 60°C and briefly sonicated again. For details, please refer to the previously published method.^44^

For all lipids, those provided in powder form were directly dissolved in molecular‐grade ethanol (Sigma, #E7023) to 100 mM as stock. For lipids supplied in chloroform, the solvent was first evaporated under a gentle nitrogen stream, and the resulting lipid film was redissolved in an equivalent volume of ethanol. During this process, sonication was applied to facilitate complete dispersion of the lipids in ethanol. Prior to use, aliquots and lipids in ThT‐reaction buffers were briefly sonicated again.

Lipid concentrations reflecting human pathophysiological states were obtained from the Human Metabolome Database (HMDB) along with relevant literature sources. To facilitate direct comparison, all concentration values were standardized to µM units, consistent with plasma lipid data. For brain‐derived measurements, the following conversion principles were applied:

- For values reported as nmol/mg protein, concentrations were converted to µM using the formula: nmol/mg protein × brain protein content (28‐54 mg/g)^45^ × brain density (1.03 kg/L)^46^
- For values reported as nmol/g brain wet weight, the conversion formula was: nmol/g × brain density (1.03 kg/L)^46^

### 2.7 Fibrillization kinetics assays

To identify an optimal assay buffer for monitoring Aβ fibrillization in the presence of various lipid components using ThT, three buffer systems were evaluated:

- Buffer A: 50 mM Tris, 150 mM NaCl, and pH 7.2
- Buffer B: 20 mM HEPES, 150 mM NaCl, and pH 7.2
- Buffer C: 10 mM phosphate, 150 mM NaCl, and pH 8.0

Buffer C was ultimately selected as the optimal condition based on signal consistency and reproducibility.

Fibrillization assays were conducted in 384‐well nonbinding surface microplates (Greiner, #781903), with a final reaction volume of 40 µL per well. The assay mixture included the following components:

- Lipid samples: 4 µL of ethanol‐dissolved lipids added per well, with serial dilutions ranging from 1X to 1/1024X.
- Buffer C: used as the base buffer in all wells
- ThT: added from a 200 µM stock to a final concentration of 20 µM
- Aβ42: added last into the reaction mixture from a 200 µM stock to a final concentration of 20 µM
- Negative control dyes: morin and phenol red, each added at 20 µM final from 200 µM stocks

Control groups were configured as follows:

- Blank: Buffer C only
- NTC (no template control): Buffer C + ThT
- Positive control: Buffer C + ThT + Aβ42
- Negative control: Buffer C + ThT + Aβ42 + phenol red + morin

Fluorescence‐based aggregation assays were performed using a BioTek Synergy H1 plate reader (Agilent, #1623193). Reactions were incubated at 37°C with fluorescence readings taken every 5 minutes. Prior to each read, the plate was subjected to a 20‐second double‐orbital shake. ThT fluorescence was measured with excitation at 440 nm and emission at 484 nm. Background subtraction was performed using the NTC wells containing only buffer and ThT.

### 2.8 Kinetic data presentation and statistics

To quantify the effects of different lipids on Aβ40 and Aβ42 ThT fluorescence signal and aggregation kinetics over time, a repeated measures analysis of variance (ANOVA) was applied using the area under the curve (AUC) as the within‐subject factor. For each sample, raw fluorescence intensity–time curves collected at equally spaced time points were summarized by AUC, capturing the overall response magnitude. As all samples were measured at identical intervals (each 5 minutes), AUC values were mathematically equivalent to mean relative fluorescence unit (RFU) up to a constant factor, allowing valid group‐level comparisons.

One‐way ANOVA was then performed on AUC values to assess differences between lipid treatment groups. Outliers were identified and excluded using a leave‐one‐out z‐score method, in which each replicate was compared to others within the same group at each time point. Replicates exhibiting consistently elevated deviations across the time course were removed. This QC step was applied to all lipid/Aβ conditions, each of which included at least duplicate or triplicate measurements for statistical analysis.

This approach preserved the advantages of repeated measurements in minimizing within‐group variability while allowing a straightforward assessment of group effects on cumulative or average response. Results were reported as mean ± standard error of the mean (SEM) for each condition. Post hoc comparisons were conducted using Dunnett's test, comparing each treatment group to the positive control. *p*‐values were reported in GraphPad style: *p* > 0.05 (ns), *p* < 0.05 (*), *p* < 0.01 (**), *p* < 0.001 (***), and *p* < 0.0001 (****). Statistical analyses and visualizations were conducted using GraphPad Prism [version 10.2.1].

## 3 RESULTS

### 3.1 EV purification and quality control (QC)

EVs mediate crosstalk between adipose tissue and the brain^21^ (Figure S1A). Exosome‐enriched EVs isolated from murine adipocytes exhibited efficient brain biodistribution following tail vein administration (Figure S1B,C). To characterize the lipid signature of human EVs derived from obese adipocytes, we first isolated exosome‐enriched EVs using tangential flow filtration (TFF) followed by ultrafiltration.^47^ EVs were obtained from subcutaneous adipose tissue (SQA) and visceral adipose tissue (VA) collected from elective surgical adipose biopsies of lean and obese donors.

Isolated EVs then underwent standard QC following established protocols.^48^, ^49^ Vesicles with sizes ranging from 60 to 300 nm were isolated from the supernatant of cultured human adipocytes (Figure S1D). These vesicles were found to contain tetraspanins, such as CD63, used to validate EV identity (Figure S1E, fluorescent staining). Adiponectin, produced by adipocytes, is present in the exosome cargo (Figure S1F), confirming that these are the adipocyte‐derived EVs. Also of interest is the presence of clusterin, which plays a role in AD pathogenesis.^50^ These data quantify EVs based on particle number, size, and the presence of specific molecules, such as CD63, adhering to MISEV2018.^48^, ^49^ The QC‐validated EVs were subsequently processed for lipidomic profiling.

### 3.2 Lipidomic profiling

We performed the multidimensional mass spectrometry‐based lipidomics for the EVs derived from both lean and obese individuals (Supporting Information Table 2). The heatmap reveals distinct lipidomic profiles between lean and obese EVs, with hierarchical clustering showing clear separation between the two groups and highlighting lipid species that are differentially abundant in obesity (Figure 1A). Stratified by lipid species and individual patients, several lipid classes, including triacylglycerols (TAG), phosphatidic acids (PA), and SM, show significant differences between the lean and obese groups (Figure 1B,C).

To investigate sample‐level deviations observed in EV lipidomic clustering (Figure 1A), we applied the Isolation Forest algorithm to identify potential outliers. This analysis revealed one lean sample (Lean 1) exhibited a high anomaly score, flagging it as a potential outlier, whereas the deviating obese sample (Obese 11) was not flagged. Importantly, we found no technical or biological abnormalities (e.g., age, sex, BMI, batch effects; Supporting Information Table 1) associated with either sample. To evaluate the impact of retaining Lean 1, we conducted sensitivity analyses with and without its inclusion. key lipid species such as phosphatidylcholine (PC), PA, LPC, and fatty acyl‐Coenzyme A (FAC) remained significantly altered in obesity, regardless of whether Lean 1 was included, as confirmed by fold‐change and statistical significance analyses (Figure 1D–E and S2A–E).

The correlation heatmap reveals distinct clustering patterns among lipid species in lean and obese EVs, indicating obesity‐associated reorganization of lipid coregulation networks (Figure 1F; Supporting Information Table 4). Hierarchical clustering identified ten discrete lipid modules (clusters a–j), each enriched for specific lipid classes, such as SM, TAG, LPE, or PC. The corresponding fold changes (obese/lean) for lipid species within each cluster further illustrate their differential abundance and distribution between obese and lean individuals (Figure 1F; Supporting Information Table 5).

Furthermore, the clear separation of LPC and SM between lean and obese groups across various lipid classes indicates significant differences in EV lipid composition that remain consistent irrespective of Lean 1 inclusion (Figure 1G and S2F). When Lean 1 was excluded (Figure S2F), the lean group exhibited markedly reduced intragroup variation, suggesting that Lean 1 captures a unique aspect of biological heterogeneity within the lean cohort. As the overall findings remained consistent with or without Lean 1, we retained this sample in all downstream analyses to preserve biological variability and minimize selection bias. Notably, SM were predominantly downregulated, while PC, LPC, and LPE were primarily upregulated in obesity (Figure 1H).

To determine the extent to which EVs reflect the lipid composition of adipose tissue (Supporting Information Table 3) in mediating adipose tissue–brain crosstalk, we conducted a comparative analysis of the two compartments. Among the detected lipids, the lipidomic comparison revealed a selective overlap of FAC, TAG, diacylglycerol (DAG), PC, PE, PS, SM, LPE, and PI (Figure 2A), with a similar lipid composition ratio (Figure 2B), suggesting a conserved lipid distribution pattern. Both SM and PA were identified as signature lipid classes in both EVs and adipose tissue (Figure 2C).

In the network association analysis, phospholipids such as PC, PA, and LPE exhibit strong interconnections in EVs (Figure 2D), whereas TAG dominates the coregulation network in adipose tissue (Figure 2E). When excluding TAG, PE and PC emerge as the primary connectors within the adipose tissue network (Figure 2F). When restricting the analysis to the top 20% most abundant lipid species in EVs and adipose tissue (from Figure 2D  to 2G; from Figure 2E to 2H), we observed that overall network connectivity was notably reduced (Figure 2I), as reflected by sparser interlipid associations in both groups (Figure 2G–H). This finding suggests that the majority of lipid coregulation occurs across the full lipidome rather than being driven solely by the most abundant ones. Importantly, this reduction pattern in network density was consistently observed across both EVs and adipose tissue (Figure 2I), suggesting a conserved organizational principle of lipid network architecture in both systems.

These findings indicate that EVs reflect the lipidomic profile of adipose tissue to a certain extent, with selective lipid enrichment and preserved network associations. This reinforces the hypothesis that EVs serve as lipid carriers facilitating adipose–brain crosstalk.

### 3.3 Aβ fibrillization evaluation

**FIGURE 3:**
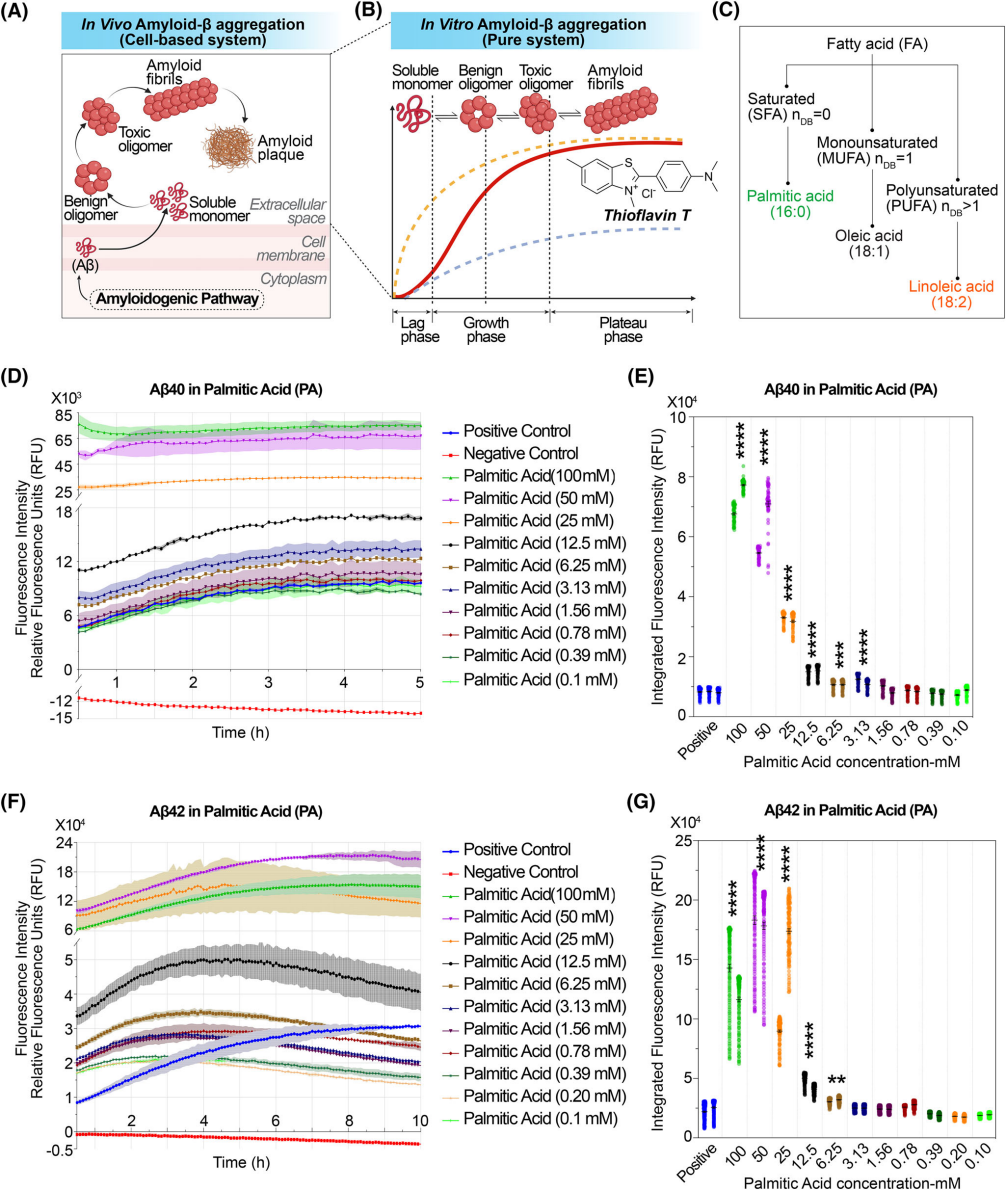
In vivo and in vitro models of amyloid‐β (Aβ) aggregation and modulation by palmitic acid. (A,B) Conceptual overview of Aβ aggregation pathways under in vivo and in vitro conditions. (A) Schematic of in vivo Aβ aggregation in a cell‐based system. Soluble Aβ monomers generated in the cytoplasm or near the membrane transition into benign oligomers, toxic oligomers, and eventually amyloid fibrils and plaques within the extracellular space. The process involves dynamic interconversion between aggregation species and spatial localization across cellular compartments. (B) Illustration of in vitro Aβ aggregation in a purified, cell‐free system, monitored by thioflavin T (ThT) fluorescence. Soluble monomers self‐assemble into benign and toxic oligomers, which ultimately form mature amyloid fibrils. The aggregation kinetics follow a sigmoidal curve comprising a lag phase, growth phase, and plateau phase. Different ThT fluorescence curves (dashed lines) represent modulation of fibrillization kinetics under various experimental conditions. (C) Classification of fatty acids based on the number of double bonds (n_DB). Fatty acids (FAs) are categorized into saturated fatty acids (SFAs; n_DB = 0), monounsaturated fatty acids (MUFAs; n_DB = 1), and polyunsaturated fatty acids (PUFAs; n_DB > 1). Representative examples of each class are shown: palmitic acid (16:0) as an SFA, oleic acid (18:1) as a MUFA, and linoleic acid (18:2) as a PUFA. (D‐G) Experimental kinetics for Aβ40 and Aβ42 aggregation under varying concentrations of palmitic acid (PA).   (D and F) Aggregation kinetics of Aβ40 and Aβ42 in the presence of serially diluted palmitic acid (0.1–100 mM), using the ThT fluorescence assay. Fluorescence intensity is shown as relative fluorescence units (RFU) at Ex/Em = 440/484 nm. Positive control: Aβ40 or Aβ42 + ThT; negative control: Aβ40 or Aβ42 + ThT + phenol red + morin. (E,G) Quantification of aggregation kinetics at varying concentrations. Each dot represents an individual fluorescence measurement (RFU) at a specific time point for the indicated lipid concentration. Bars indicate the mean ± SEM for each group. Asterisks (*) and hash symbols (#) indicate statistical significance compared to the Positive control group. Statistical approach consistent with Methods Section 2.8. Statistical comparisons were conducted using an ordinary one‐way analysis of variance (ANOVA), with removal of outliers as appropriate. Post hoc multiple comparisons were performed using Dunnett's test to compare each treatment group against the positive control. *p*‐values were reported in GraphPad style, with significance thresholds as follows: *, increase; ^#^, decrease; *p* > 0.05 (ns), *p* < 0.05 (*^/#^), *p* < 0.01 (**^/##^), *p* < 0.001 (***^/###^), and *p* < 0.0001 (****^/####^).

The aforementioned lipid components in EVs derived from human adipocytes primarily consist of phospholipids and SM. Regardless of lipid class, all contain fundamental fatty acid chains. Our systematic profiling of EVs and adipose tissue revealed that both are enriched in unsaturated fatty acid chains (Figure S2G–I). Given the diverse effects of lipids on AD, we specifically focused on their role in Aβ fibrillization, as amyloid aggregation is a well‐defined pathological hallmark of AD and a critical step in disease progression. Investigating how lipids influence Aβ fibrillization provides direct mechanistic insights into their contribution to amyloid pathology and offers a refined framework for understanding lipid‐mediated neurodegenerative processes (Figure 3A).

To ensure a high‐purity background, we employed an in vitro ThT fluorescence assay to assess the lipid‐type‐specific effects on Aβ fibrillization (Figure 3B). Because Aβ aggregation occurs primarily in the brain's extracellular space (Figure 3A,B), lipidomic profiles reflecting this microenvironment would, in principle, provide the most physiologically relevant context. However, currently available brain lipidomic datasets are largely derived from whole‐tissue homogenates or region‐specific bulk analyses, representing mixed cell populations and lacking specificity for the extracellular compartment where Aβ aggregation occurs.^51^ Moreover, many of these studies report only relative abundances, with limited data on absolute lipid concentrations.^52^, ^53^

Given these limitations, the lipid concentrations used in this study were primarily guided by reported physiological and pathophysiological levels in human plasma. Where available, we also compiled absolute concentration data from human brain and cerebrospinal fluid (CSF) lipidomics to provide a more comprehensive frame of reference. Together, these datasets offer a valuable basis for interpreting the relevance of lipid exposures to Aβ aggregation dynamics. Based on our analysis (Figure S2G–I), we first evaluated the effects of saturated and unsaturated fatty acids, using palmitic acid and oleic acid as representative molecules (Figure 3C). Both are key lipid components broadly represented across multiple EV lipid classes detected in our lipidomic dataset and were directly detected as constituent lipid species within the EV lipidome.

### 3.4 Fatty acids and Aβ fibrillization

Palmitic acid, also known as hexadecanoic acid, is one of the most common saturated long‐chain fatty acids (C16:0) in mammals, comprising approximately 21%–30% (molar) of human depot fat. In healthy adults, the blood concentration of palmitic acid can reach 2360 ± 430 µM in males and 2500 ± 630 µM in females,^54^ while lower physiological levels have been reported around 30.49 ± 2.64 µM.^55^ Additionally, reported values in the human CSF of normal adults are 18.0 ± 12.0 µM,^56^ and in human brain tissue, palmitic acid was measured at approximately 496 ± 123 nmol/g (∼510.88 ± 126.69 µM) wet weight in control subjects.^57^ Under lipotoxic conditions (25–100 mM), both Aβ40 and Aβ42 exhibit pronounced aggregation (Figure 3D–G). At 3.13 mM, a concentration representative of pathophysiological overload, palmitic acid significantly promotes Aβ40 aggregation (Figure 3D,E), whereas its effect on Aβ42 is minimal (Figure 3F,G). At lower concentrations, palmitic acid shows negligible influence on the fibrillization of either Aβ40 or Aβ42, with aggregation rates comparable to the positive control, indicating a baseline, nonpromotive state under physiological conditions.

Before examining the effects of additional lipid species, several methodological considerations warrant clarification to aid in the interpretation of the Aβ aggregation data. The apparent lack of sharply defined lag, growth, and plateau phases (Figure 3D) is primarily due to the wide concentration range (0.1 to 100 mM) of palmitic acid presented in a single plot. This required an expanded *y*‐axis scale to accommodate high‐signal conditions, which in turn visually compressed the kinetic transitions. When only intermediate concentrations were examined (Figure S3A), the classical sigmoidal features of Aβ aggregation became more distinguishable. However, to preserve comparability across all tested concentrations, a unified plot was retained (Figure 3D)

In our optimized assay system (Method section 2.7), Aβ aggregation proceeds more rapidly than conventional chronic protocols which typically span 20–150 hours.^58^, ^59^ The “time zero” (Figure 3D) refers to the first fluorescence readout, which was collected approximately 20‐30 minutes after the reaction was initiated. This short delay, caused by sample mixing and plate handling, means partial preaggregation may have already occurred before the first measurement. As a result, some lipid‐treated samples displayed elevated fluorescence at the initial time point, and lag phases appeared less distinct compared to longer‐duration assays. To preserve potentially informative early kinetic differences, fluorescence values were not normalized to a uniform baseline across conditions (Figure 3D). This decision was made to avoid artificially masking early seeding effects or subtle differences in nucleation kinetics.

In the Aβ aggregation assay, area under the curve (AUC) quantifies the overall extent of Aβ aggregation over time, as reflected by the integrated kinetic fluorescence signal. To assess overall Aβ aggregation under different lipid concentrations, AUC could be calculated by numerical integration of relative fluorescence unit(RFU) over time using the trapezoidal rule, a method well‐suited for discrete, uniformly spaced time‐series data. Since fluorescence measurements were collected at consistent 5‐minute intervals, the AUC is effectively proportional to the cumulative RFU values, and their average reliably reflects aggregation kinetics across conditions (Figure 3E).

To address potential concerns regarding nonzero initial baselines—a consequence of preaggregation during sample handling prior to the first readout—we reconstructed simulated curves by extrapolating back to the actual reaction start time (Figure S3B). These simulations showed that regardless of the integration start point, experimental and control groups are statistically distinguishable, supporting the reproducibility, robustness, and validity of RFU‐based comparisons in capturing both early seeding dynamics and the total extent of Aβ aggregation.

Oleic acid, also known as octadecenoic acid, is a widely distributed and abundant long‐chain monounsaturated fatty acid (C18:1, omega‐9). In healthy adults, oleic acid concentrations range from 11.42 ± 1.67 to 2135.4 ± 665.5 µM (*n* = 54),^55^, ^60^ with peak levels in pathological states reaching up to 2365.1 ± 844.5 µM.^60^ Additionally, oleic acid concentrations in human CSF have been reported at 36.0 ± 36.0 µM in healthy adults,^56^ and human brain tissue levels measured at ∼367  ±  123 nmol/g (∼378.01 ± 126.69 µM) wet weight in control subjects.^57^ Under lipotoxic conditions (6.25–50 µM), Aβ40 undergoes severe fibrillization (Figure S3C,D). Notably, even at physiopathological concentrations (0.39–3.23 µM), Aβ40 aggregation remains evident (Figure S3C,D). Unexpectedly, at moderate concentrations (0.1–0.2 mM, or 100‐200 µM), the aggregation rate of Aβ40 is significantly lower than that of the positive control (Figure S3C,D). Surprisingly, Aβ42 exhibits robust aggregation across all tested concentrations, both low and high (Figure S3E–F).

In the experiments described above, the convergent finding is that both saturated and unsaturated fatty acids can promote Aβ fibrillization at relatively high concentrations approaching lipotoxic conditions. Notably, the same fatty acid exerts differential effects on Aβ40 and Aβ42 aggregation. A divergent observation is that palmitic acid, the most abundant saturated fatty acid in vivo, does not influence baseline Aβ aggregation at physiological concentrations. Our study expands the known benefits of unsaturated fatty acids. In addition to their classical antioxidant effects—attributed to the presence double bonds—we observed that their lipid characteristics confer an additional advantage: at physiological concentrations (100–200 µM), unsaturated fatty acids can suppress the spontaneous fibrillization of Aβ40.

### 3.5 SM and Aβ fibrillization

 SM exhibited pronounced deregulation in our human EV lipidomics profiling (Figure 1G,H). SM is abundantly present in the nervous system, where it plays a critical role in maintaining the structural integrity of cellular membranes. It is also a key component of the myelin sheath, particularly in the membranous layers that insulate neuronal axons.^61^ To further investigate the role of SM in Aβ fibrillization, we selected three physiologically relevant SM sources: milk SM, egg SM, and brain SM, which are primarily composed of SM 23:0, SM 16:0, and SM 18:0, respectively. Each of these SM species was among the detected lipid constituents in our EV lipidomic analysis.

Among these, SM (23:0) is the least studied in terms of physiological and pathological concentrations, with limited reports indicating a plasma level of 7 ± 2.7 µM.^62^ SM (16:0) is more commonly detected in human plasma, ranging from 60 to 190 µM in both males and females under normal conditions (HMDB‐Human metabolome database).^63^, ^64^ SM (18:0) is reported to reach concentrations of 1145 ± 67 or 1364 ± 106 µM in adult females,^65^ while levels as low as 16.2 ± 0.4 µM have also been documented in healthy male and female individuals.^62^

In human CSF, SM (16:0) and SM (18:0) have been detected at concentrations of 0.336  ±  0.109 and 0.340  ±  0.154 µM,^66^ respectively, in adults over 18 years of age. In contrast, no CSF data are currently available for SM (23:0), highlighting a gap in the lipidomic characterization of this species within central nervous system compartments. Although absolute concentrations of these SM species have been reported in various brain regions of mice,^67^ corresponding quantitative data in the human brain remain scarce.

**FIGURE 4:**
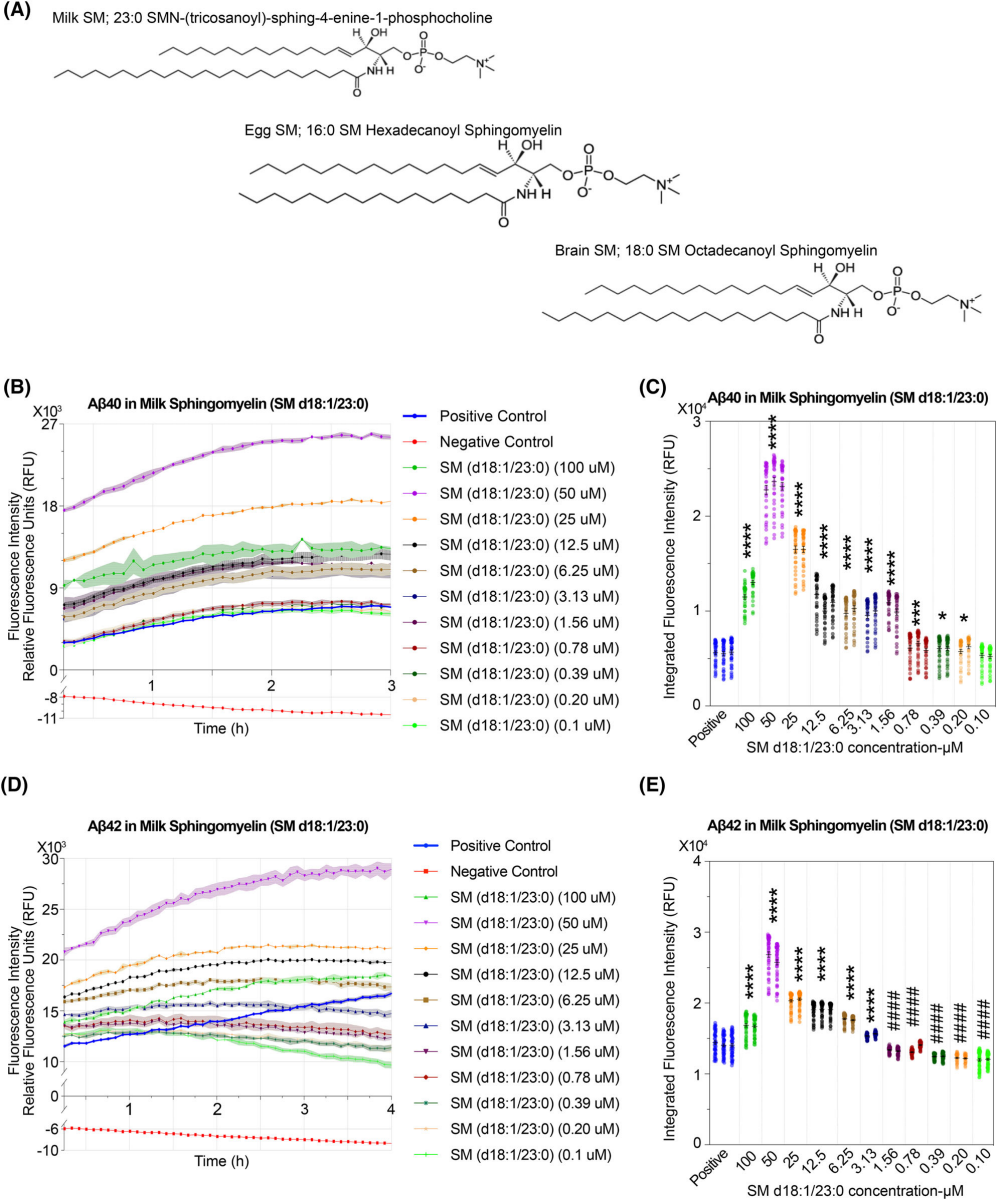
Experimental kinetics of amyloid‐β (Aβ) aggregation in the presence of milk sphingomyelin. (A) Molecular structure of lipids of Milk sphingomyelin (SM), Egg SM, and Brain SM. (B–E) Experimental kinetics for Aβ40 and Aβ42 aggregation under varying concentrations of Milk SM. (B, D) Aggregation kinetics of Aβ40 and Aβ42 in the presence of serially diluted Milk SM (0.1–100 mM), using the thioflavin T (ThT) fluorescence assay. Fluorescence intensity is shown as relative fluorescence units (RFU) at Ex/Em = 440/484 nm. Positive control: Aβ40 or Aβ42 + ThT; negative control: Aβ40 or Aβ42 + ThT + phenol red + morin. (C, E) Quantification of aggregation kinetics at varying concentrations. Each dot represents an individual fluorescence measurement (RFU) at a specific time point for the indicated lipid concentration. Bars indicate the mean ± SEM for each group. Asterisks (*) and hash symbols (#) indicate statistical significance compared to the Positive control group. Statistical approach consistent with Methods Section 2.8, with significance thresholds as follows: *, increase; ^#^, decrease; *p* > 0.05 (ns), *p* < 0.05 (*^/#^), *p* < 0.01 (**^/##^), *p* < 0.001 (***^/###^), and *p* < 0.0001 (****^/####^).

In our ThT‐based fibrillization assays, SM 23:0 significantly enhanced Aβ aggregation at higher concentrations, including at physiologically relevant levels near 7 µM (Figure 4B–E). However, this proaggregation effect was markedly attenuated at lower concentrations. Notably, in the Aβ42 system, SM 23:0 at concentrations below 2 µM appeared to suppress fibrillization over time (Figure 4D,E), suggesting a potential biphasic effect.

In contrast, SM 16:0 consistently promoted Aβ aggregation across both high and low concentration ranges (Figure S4). For Aβ40, a threshold concentration for fibrillization enhancement was observed at 25 µM, while Aβ42 showed a peak proaggregation response at approximately 100 µM. These differing peak responses imply that Aβ40 and Aβ42 interact with SM 16:0 in distinct structural conformations, leading to divergent fibrillization kinetics. SM 18:0, the predominant component of brain‐derived sphingomyelin, exhibited a strong proaggregation effect on Aβ42. Its effect on Aβ40 was relatively moderate, particularly at lower concentrations, where little to no promotion of aggregation was observed (Figure S5).

These findings suggest that certain sphingomyelin species, particularly SM (23:0), can significantly inhibit Aβ42 fibrillization at relatively low, physiologically relevant concentrations, potentially contributing to the attenuation of AD‐related amyloid pathology. In contrast, SM (18:0) and SM (16:0) did not exhibit such inhibitory effects under similar conditions. This highlights that, despite sharing a common sphingomyelin backbone, variations in acyl chain length and saturation can result in markedly different impacts on Aβ aggregation dynamics.

The observed differential effects may be attributed to several factors. Longer, fully saturated acyl chains such as those in SM (23:0) may confer distinct biophysical properties, such as increased membrane rigidity or altered lipid packing, that influence Aβ interaction at the lipid interface. Alternatively, the ability of specific SM species to modulate local microenvironments—such as surface hydrophobicity or curvature stress—may selectively affect Aβ42's conformational transition and nucleation kinetics.

### 3.6 LPC and Aβ fibrillization

Lysophospholipids are bioactive lipids formed as intermediates during the dynamic turnover of membrane phospholipids.^68^ Although studies have reported altered lysophospholipid levels in AD. Region‐specific increases in LPC and LPE have been observed in the AD brain using various lipidomic techniques, including LC‐ESI‐MS and FIA‐MS/MS.^69^, ^70^, ^71^, ^72^

**FIGURE 5:**
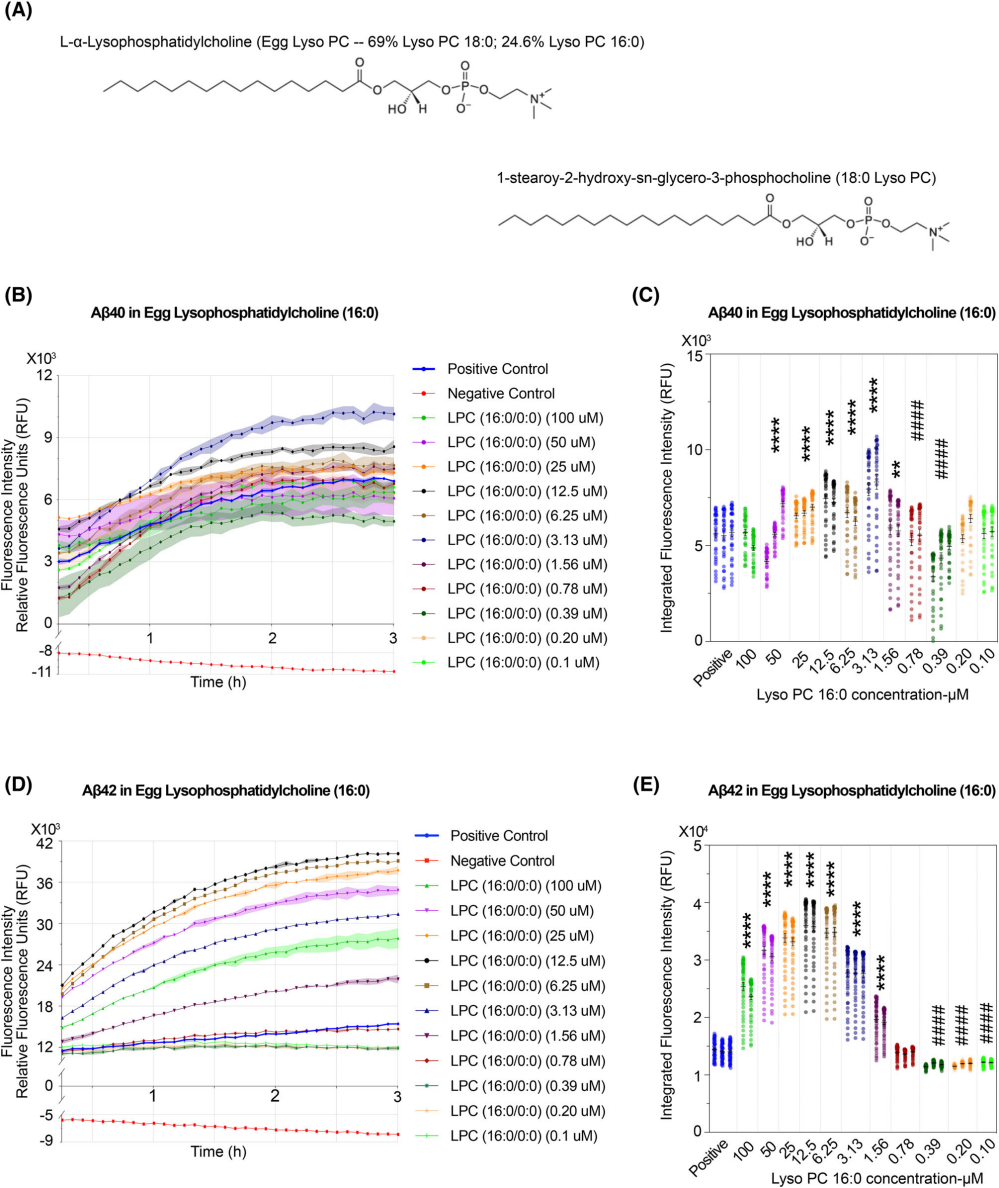
Experimental kinetics of amyloid‐β (Aβ) aggregation in the presence of egg lysophosphatidylcholine (LPC; Lyso PC). (A) Molecular structure of lipids of egg Lyso PC and 18:0 Lyso PC. (B‐E) Experimental kinetics for Aβ40 and Aβ42 aggregation under varying concentrations of LPC (16:0). (B and D) Aggregation kinetics of Aβ40 and Aβ42 in the presence of serially diluted LPC (16:0) (0.1–100 mM), using the ThT fluorescence assay. Fluorescence intensity is shown as relative fluorescence units (RFU) at Ex/Em = 440/484 nm. Positive control: Aβ40 or Aβ42 + ThT; negative control: Aβ40 or Aβ42 + ThT + phenol red + morin. (C, E) Quantification of aggregation kinetics at varying concentrations. Each dot represents an individual fluorescence measurement (RFU) at a specific time point for the indicated lipid concentration. Bars indicate the mean ± SEM for each group. Asterisks (*) and hash symbols (#) indicate statistical significance compared to the Positive control group. Statistical approach consistent with Methods Section 2.8, with significance thresholds as follows: *, increase; ^#^, decrease; *p* > 0.05 (ns), *p* < 0.05 (*^/#^), *p* < 0.01 (**^/##^), *p* < 0.001 (***^/###^), and *p* < 0.0001 (****^/####^).

In our study, EV lipidomic profiling revealed distinct, nonoverlapping clusters and marked deregulation of LPC species between obese and lean individuals (Figure 1G,H). We selected egg LPC and LPC 18:0 for Aβ aggregation assays (Figure 5A). Both species were directly detected in our EV lipidomics dataset. Egg LPC consists of approximately 69% LPC 16:0 and 24% LPC 18:0. In healthy individuals (both male and female), circulating LPC 16:0 levels range from 41 to 150 µM (HMDB), with reported averages of 106.6  ±  16.7 and 141  ±  50 µM.^10^ Under abnormal condition, levels may rise to 142.1  ±  39.6 µM in females.^73^ In comparison, physiological LPC 18:0 levels are typically lower, ranging from 9.1 to 54 µM (HMDB), and may reach up to 48.5  ±  20.2 µM in healthy individuals in both genders.^74^ LPC 18:0 has been reported at 0.069  ±  0.019 µM in adults.^66^

In our ThT‐based fibrillization assays, LPC species demonstrated concentration‐dependent effects on Aβ aggregation. At higher pathophysiological concentrations (1.56 to 100 µM), LPC 16:0 significantly promoted the aggregation of both Aβ40 and Aβ42 (Figure 5B–E). This proaggregatory effect diminished at lower LPC 16:0 concentrations. However, considering the reported physiological and pathological plasma levels of LPC 16:0, such low concentrations are infrequent in vivo, suggesting that the observed inhibitory effects may have limited physiological relevance. These findings imply that under conditions such as obesity, elevated LPC levels transported to the brain via extracellular vesicles could enhance Aβ fibrillization.​

Unexpectedly, LPC 18:0 exhibited a robust proaggregatory effect on Aβ40 across all tested concentrations, surpassing the positive control by approximately threefold (Figure S6A,B). In contrast, its influence on Aβ42 aggregation was less pronounced, with significant yet modest fold changes observed at both high and low concentrations (Figure S6C,D).​

The evidence indicates that LPC exerts differential effects on Aβ aggregation, varying with concentration and Aβ subtype. Notably, under obesity‐related conditions, elevated LPC levels transported to the brain via EVs may exacerbate Aβ fibrillization. These findings underscore the intricate role of lipid metabolism in AD pathogenesis and suggest that lipid dyshomeostasis is integral to AD pathology.

### 3.7 PE and Aβ fibrillization

**FIGURE 6:**
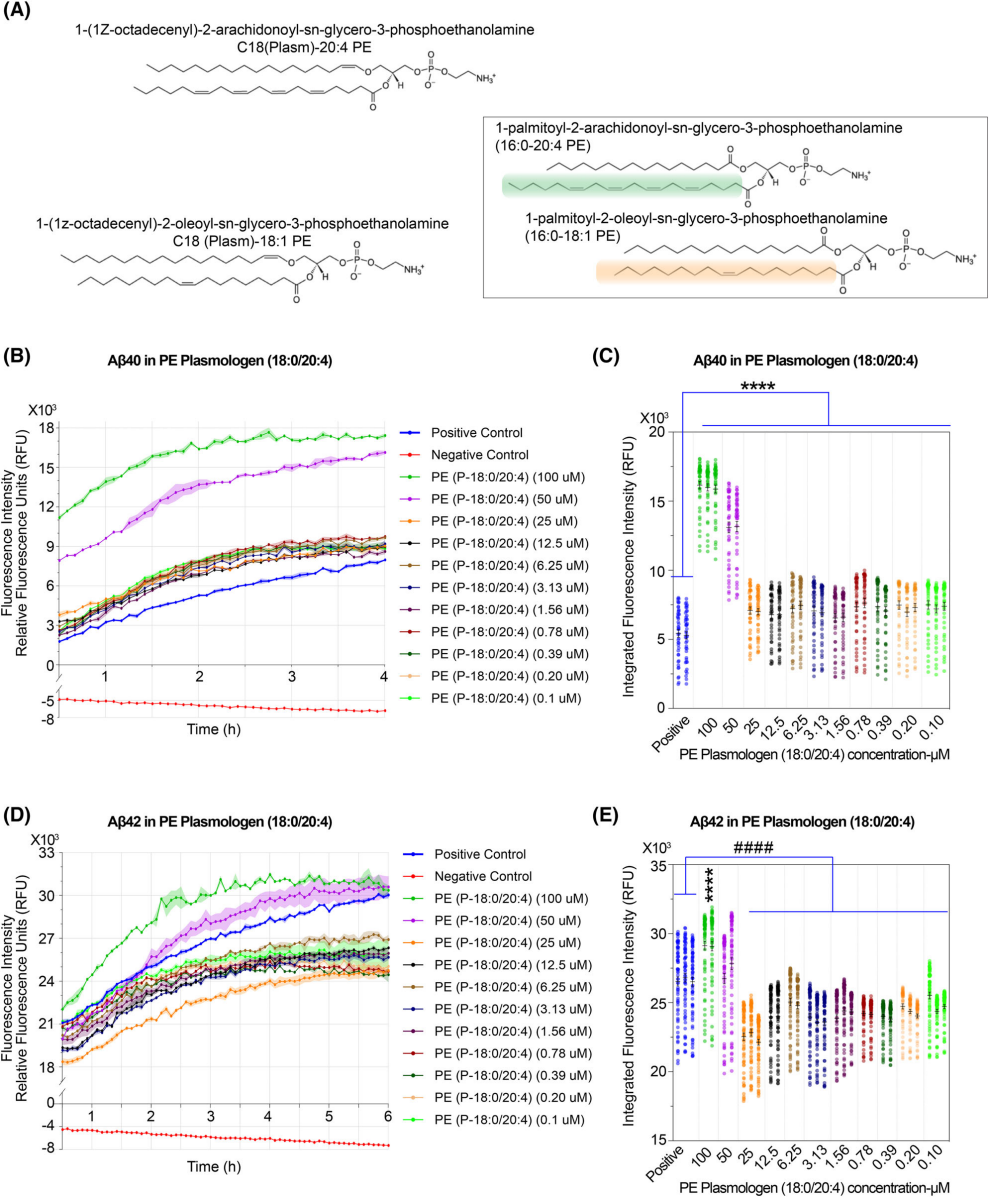
Experimental kinetics of amyloid‐β (Aβ) aggregation in the presence of C18(Plasm)‐20:4 phosphatidylethanolamine (PE). (A) Molecular structure of lipids of C18(Plasm)‐20:4 PE, C18(Plasm)‐18:1 PE, 16:0‐20:4 PE, and 16:0‐18:1 PE. (B‐E) Experimental kinetics for Aβ40 and Aβ42 aggregation under varying concentrations of C18(Plasm)‐20:4 PE. (B and D) Aggregation kinetics of Aβ40 and Aβ42 in the presence of serially diluted C18(Plasm)‐20:4 PE (0.1‐100 mM), using the thioflavin T (ThT) fluorescence assay. Fluorescence intensity is shown as relative fluorescence units (RFU) at Ex/Em = 440/484 nm. Positive control: Aβ40 or Aβ42 + ThT; negative control: Aβ40 or Aβ42 + ThT + phenol red + morin. (C,E) Quantification of aggregation kinetics by calculating the area under the fluorescence curve (AUC) for each concentration. AUC values reflect the extent of Aβ40/ Aβ42 fibrillization. Asterisks (*) and hash symbols (#) indicate statistical significance compared to the Positive control group. Statistical approach consistent with Methods Section 2.8, with significance thresholds as follows: *, increase; ^#^, decrease; *p* > 0.05 (ns), *p* < 0.05 (*^/#^), *p* < 0.01 (**^/##^), *p* < 0.001 (***^/###^), and *p* < 0.0001 (****^/####^).

In EV lipidomic profiles, lipid species such as PC, SM, TAG, and LPC were prominently detected, likely reflecting their high intrinsic abundance, efficient ionization, and preferential incorporation into EVs during biogenesis, underscoring their structural and functional relevance in EV composition. To explore the contribution of lipids beyond those enriched in EVs, we included another Zwitterionic lipid, PE in our Aβ aggregation assays (Figures 6 and S7‐S9), given its high abundance in neuronal membranes and its known biophysical effects on membrane curvature and protein–lipid interactions.^75^, ^76^, ^77^

Plasmalogen PEs, defined by a vinyl ether linkage at the sn‐1 position, are enriched in the brain and have been shown to protect against oxidative stress.^78^ Changes in their levels indicate peroxisomal dysfunction, implicating redox imbalance and lipid metabolism disturbances commonly observed in metabolic and neurodegenerative diseases such as diabetes and AD.^79^, ^80^ Based on our lipidomics profiling, we selected C18(Plasm)‐20:4 PE, C18(Plasm)‐18:1 PE, 16:0/20:4 PE, and 16:0/18:1 PE for ThT‐based fibrillization assays (Figure 6A). These PE species were not among those directly detected in our current EV lipidomics dataset.

According to concentration estimates from the HMDB, C18(Plasm)‐20:4 PE ranges from approximately 3.717  ±  0.679 to 28.290  ±  21.925 µM in the human circulatory system, while C18(Plasm)‐18:1 PE ranges from 2.160  ±  0.374 to 27.865  ±  22.648 µM. For diacyl PE species, concentrations estimated via MetaboAnalyst indicate that 16:0/20:4 PE ranges from 6.002  ±  2.136 to 52.133  ±  21.658 µM, and 16:0/18:1 PE ranges from 3.660  ±  1.734 to 41.967  ±  12.350 µM.

In human brain, all four PE species—C18(Plasm)‐20:4 PE, C18(Plasm)‐18:1 PE, 16:0/20:4 PE, and 16:0/18:1 PE—were detected across multiple brain regions, including the frontal, parietal, and temporal cortices, as well as the cerebellum.^81^ Notably, C18(Plasm)‐20:4 PE (P16:0‐22:4/P18:0‐20:4) was the most abundant, with concentrations ranging from ∼20 to 54 nmol/mg protein (∼576.8 to 1557.36 µM), and showed a marked reduction in individuals with higher Clinical Dementia Rating (CDR) scores.^81^ C18(Plasm)‐18:1 PE (P18:0‐18:1/P16:0‐20:1) also demonstrated broad regional presence (∼7–32 nmol/mg protein (∼201.88–922.88 µM)) and a similar CDR‐dependent decline.^81^ In contrast, the diacyl species 16:0/20:4 PE and 16:0/18:1 PE were present at lower levels (∼1.4–5.4 nmol/mg protein (∼40.376–155.736 µM)) and exhibited minimal changes with CDR.^81^ These results suggest a selective vulnerability of plasmalogen PE species.

In our ThT‐based fibrillization assays, both C18(Plasm)‐20:4 PE and C18(Plasm)‐18:1 PE markedly promoted Aβ40 and Aβ42 aggregation at supraphysiological concentrations (Figures 6B–E and S7A–D). Interestingly, under concentrations approximating physiological levels, these plasmalogen PEs continued to enhance Aβ40 aggregation (Figures 6B–C and S7A,B), but exhibited a significant inhibitory effect on Aβ42 aggregation (Figures 6D–E and S7C,D). Similarly, both 16:0/20:4 PE and 16:0/18:1 PE showed a strong proaggregatory effect on Aβ40 (Figures S8A–B and S9A,B), with 16:0/18:1 PE displaying a dose‐dependent response, except at the lipid‐toxic concentration of 100 µM (Figure S9A,B). For Aβ42, 16:0/20:4 PE significantly promoted aggregation at moderate to high concentrations (Figure S8C,D), whereas 16:0/18:1 PE elicited variable responses across low and high concentrations (Figure S9C,D).

These results indicate that both the chemical structure and concentration of PE species critically influence Aβ aggregation dynamics. Plasmalogen PEs exhibit isoform‐specific and concentration‐dependent effects—enhancing Aβ40 aggregation while suppressing Aβ42 aggregation at physiological levels. In contrast, diacyl PEs (e.g., 16:0/20:4 and 16:0/18:1 PE) consistently promote Aβ40 aggregation, with 16:0/18:1 PE displaying a clear dose‐dependent response. However, their effects on Aβ42 aggregation appear less predictable. Collectively, these findings suggest that lipid structural remodeling may selectively modulate amyloidogenic processes in AD.

## 4 DISCUSSION

Obesity is increasingly recognized as one of the most prominent modifiable risk factors for dementia, including AD.^2^ A substantial body of evidence, from both long‐term and recent studies, has demonstrated that high‐fat diet (HFD) feeding induces neuroinflammation and cognitive decline in animal models and humans.^6^, ^82^, ^83^, ^84^ EVs have been identified as key mediators of adipose tissue–brain communication,^21^ underscoring their potential to convey obesity‐related signals that influence central nervous system (CNS) function. Even beyond obesity, disruptions in lipid homeostasis are critical in AD pathogenesis,^7^, ^8^ highlighting the relevance of studying adipose‐derived EV lipids in the broader context of neurodegenerative disease.

In this study, we first employed an EV tracking strategy combined with organ‐specific analysis to evaluate the biodistribution of adipose‐derived EVs in the brain (Figure S1B,C). Building on this foundational insight, we performed rigorous QC of the isolated EVs, adhering to the guidelines provided by the international society forextracellular vesicles (ISEV) and other established protocols,^48^, ^85^, ^86^ including particle size and concentration analysis using microfluidic resistive pulse sensing (MRPS) (Figure S1D), immunomagnetic capture of EV markers (Figure S1E), and Western blotting to assess adipocyte‐ and exosome‐enriched markers (Figure S1F). Together, these steps ensured high‐quality EV preparations and minimized the risk of confounding artifacts in subsequent analyses.

Next, we conducted a comprehensive lipidomics profiling of these EVs. Although most prior studies have focused on murine adipocyte‐derived EVs,^86^ our systematic study focused on human‐derived samples, thus increasing the clinical relevance and translational potential of our findings. To further investigate the lipid alterations associated with obesity, we conducted both a standalone lipidomic analysis of adipocyte‐derived EVs and a comparative analysis with lipidomic profiles from human adipose tissue. We identified a subset of lipids that were markedly deregulated in EVs derived from obese individuals (Figure 1H). Among these, LPC and SM emerged as two lipid classes that consistently segregated lean and obese samples into nonoverlapping clusters (Figure 1G), highlighting their strong linkage to metabolic dysregulation and underscoring their potential as clinically relevant biomarkers.

These lipids are known to participate in a variety of intracellular processes and extracellular signaling events. Elevated levels of LPC, for example, are associated with cellular damage, potentially through mechanisms such as altering membrane permeability and disrupting osmotic balance.^87^ DESI‐based mass spectrometry imaging has identified spatial colocalization of lysophospholipids with Aβ aggregates in AD brains, suggesting LPC may play a direct role in modulating Aβ pathology.^88^ Similarly, SM plays an essential role in intracellular signaling. As a major component of sphingolipids, it can function as a lipid second messenger regulating cellular stress responses, proliferation, differentiation, and neuronal survival.^89^, ^90^ Within membrane microdomains such as lipid rafts, sphingolipids—together with cholesterol—modulate the activity of transmembrane proteins,^91^ which are critical for synaptic function and neuronal communication. These findings raise the intriguing hypothesis that direct interactions between Aβ and these lipid classes may influence Aβ conformation, ultimately modulating aggregation propensity in AD and contributing to AD progression.

Because adipocyte‐derived EVs can penetrate the blood–brain barrier (BBB) and accumulate in the brain parenchyma,^21^ their cargos—including DNA/RNA molecules, proteins, and lipids—may have diverse impacts on neuronal health. For instance, EV‐associated microRNAs have been shown to induce synaptic damage and cognitive impairment,^21^ while specific EV lipid components may further disrupt or remodel local lipid homeostasis in the CNS. Given the multifaceted nature of EV cargo, we isolated the specific impact of lipids on Aβ fibrillization without confounding effects from other molecular components. To achieve this, we employed a ThT‐based in vitro fibrillization assay using a pure system containing only synthetic human Aβ peptides—rather than mouse‐derived Aβ—to maximize clinical relevance. Although lipid–Aβ interactions have been probed in bilayer membrane models,^33^, ^92^ such systems often comprise complex lipid mixtures, confounding the role of individual lipid species.

By contrast, our pure lipid–Aβ system allows for direct and specific interactions with minimal confounders, likely explaining the accelerated fibrillization kinetics observed. Interestingly, the ThT fluorescence curves exhibited a rapid increase reminiscent of seeded or secondary nucleation‐like aggregation, rather than the slower kinetics typically seen with spontaneous Aβ monomer aggregation.^93^ This suggests that under these simplified conditions, direct interactions between lipid molecules and Aβ may facilitate nucleation or conformational shifts that expedite the aggregation process.

A growing body of literature supports the notion that Aβ exhibits strong affinity for lipid molecules.^18^, ^32^, ^33^, ^94^ Moreover, our study provides important new insights by systematically examining multiple lipid species over a range of biologically relevant concentrations, extending from pathophysiologically high (lipotoxic) to normal physiological levels. This approach also encompassed both Aβ40 and Aβ42 monomers, direct evidence that the same lipid species can exert distinct effects on different Aβ isoforms. Our data suggested that:

### 4.1 Concentration‐dependent lipid effects

The influence of lipid concentration on Aβ aggregation varies significantly among lipid types. Certain lipids exhibited a clear dose‐dependent promotion of Aβ aggregation, as reflected by stepwise changes in kinetic parameters such as lag time (*T*_lag_), half‐maximal fluorescence time (*T_1/2_*), and growth phase duration (*T*_grow_), promoting fibrillization at high concentrations yet inhibiting it at lower levels.

Others showed less predictable patterns, signifying complex lipid–peptide interactions. A consistent observation across multiple lipid species, including oleic acid (Figure S3), egg‐derived lysoPC (Figure 5), egg SM (Figure S4), and brain SM (Figure S5), was a biphasic response, in which Aβ aggregation peaks at intermediate lipid concentrations and decreases at both lower and higher levels. Although the underlying mechanism is not fully understood, this trend may reflect a concentration range that promotes fibrillization. These effects may involve changes in condensate dynamics, as recent studies have implicated lipid‐mediated phase separation as a key modulator of Aβ aggregation dynamics.^33^, ^95^, ^96^ In addition, steric hindrance and molecular crowding^97^ may also contribute to this biphasic phenomenon. In the present study, we focused primarily on direct lipid–Aβ interactions rather than lipid–lipid assembly or supramolecular organization. However, lipid–lipid interactions could indirectly influence Aβ aggregation by reshaping membrane curvature, surface tension, or fluidity—all factors known to affect amyloid formation.^20^, ^36^, ^37^, ^98^ Collectively, these factors may explain why aggregation peaks at intermediate lipid concentrations, potentially through convergent or distinct mechanisms, although further molecular‐level validation is warranted.

### 4.2 Isoform‐specific responses

The same lipid species could have either similar or distinct effects on Aβ40 and Aβ42 aggregation, suggesting that structural differences between the two isoforms may influence their lipid‐binding properties.

It is well established that Aβ neurotoxicity follows the hierarchy of nonfibrillar oligomers > fibrils > monomers,^37^ implying any perturbation in the aggregation pathway can have profound implications for disease pathogenesis. Our findings reveal that lipid‐specific modulation of Aβ aggregation may not only alter fibrillization kinetics but shift the distribution of Aβ species toward those with distinct toxic potentials. Given that Aβ40 and Aβ42 can behave differentially in the same lipid milieu, it is plausible that their toxic potentials could also diverge—particularly under obese conditions, where lipid composition and concentration are significantly altered.

From a mechanistic standpoint, our findings highlight the critical role of lipid composition and concentration in modulating Aβ aggregation dynamics and, by extension, AD pathology. Interactions between lipid molecules and Aβ can facilitate pathological aggregation through biophysical processes such as phase separation and condensate formation,^33^ effectively creating nucleation platforms or altering local molecular crowding to accelerate oligomer or fibril formation. Crucially some lipids exhibit concentration‐dependent biphasic effects—promoting aggregation at higher levels yet inhibiting it at lower doses—highlighting the need for nuanced control of lipid microenvironments when considering therapeutic interventions or interpreting disease mechanisms.

Moreover, these findings carry important translational implications. As EVs and lipid‐based carriers gain traction as promising therapeutic delivery systems capable of crossing the BBB, rational design of their lipid constituents becomes critical. Ensuring biocompatibility and target specificity must be balanced against the risk of inadvertently driving Aβ aggregation or exacerbating neurotoxic pathways. By demonstrating that even subtle shifts in lipid composition or concentration can meaningfully alter Aβ aggregation outcomes, our work underscores the necessity for precise lipid profiling and engineering in such delivery platforms.

Finally, the lipid–Aβ specificity observed here underscores that maintaining tightly regulated lipid metabolic homeostasis in the brain is pivotal for mitigating or preventing neurodegeneration. As metabolic dysfunction and obesity continue to rise globally, their intersection with neurodegenerative diseases demands closer scrutiny. Our study contributes a foundational understanding of how discrete lipid species, present at various concentrations, can shape Aβ assembly and possibly translate into altered neuronal toxicity. Although our findings provide molecular insights using a well‐controlled pure system, we acknowledge that the current study does not establish whether EV lipid differences directly contribute to Aβ aggregation in the brain. Future studies incorporating appropriate animal models, allowing the disentanglement of potential confounding effects, will be essential to determine the physiological relevance of EV‐mediated lipid–Aβ interactions in the context of AD pathology.

## CONFLICT OF INTEREST STATEMENT

The authors declare no competing interests. Author disclosures are available in the supporting information.

## CONSENT STATEMENT

All procedures involving human participants were approved by the Institutional Review Board (IRB# 2014H0471) of The Ohio State University. Written informed consent was obtained from all individuals in accordance with institutional and ethical guidelines.

## Supporting information



Supporting Information

Supporting Information
