# Supplementary material for: Decoding adipose–brain crosstalk: Distinct lipid cargo in human adipose‐derived extracellular vesicles modulates amyloid aggregation in Alzheimer's disease
Source: Alzheimers Dement. 2025 Oct 2;21(10):e70603. doi: 10.1002/alz.70603 (PMC12489747; doi:10.1002/alz.70603)
Supplement: Supplementary file 2 — Supporting Information [file ALZ-21-e70603-s001.pdf]

Figure S1

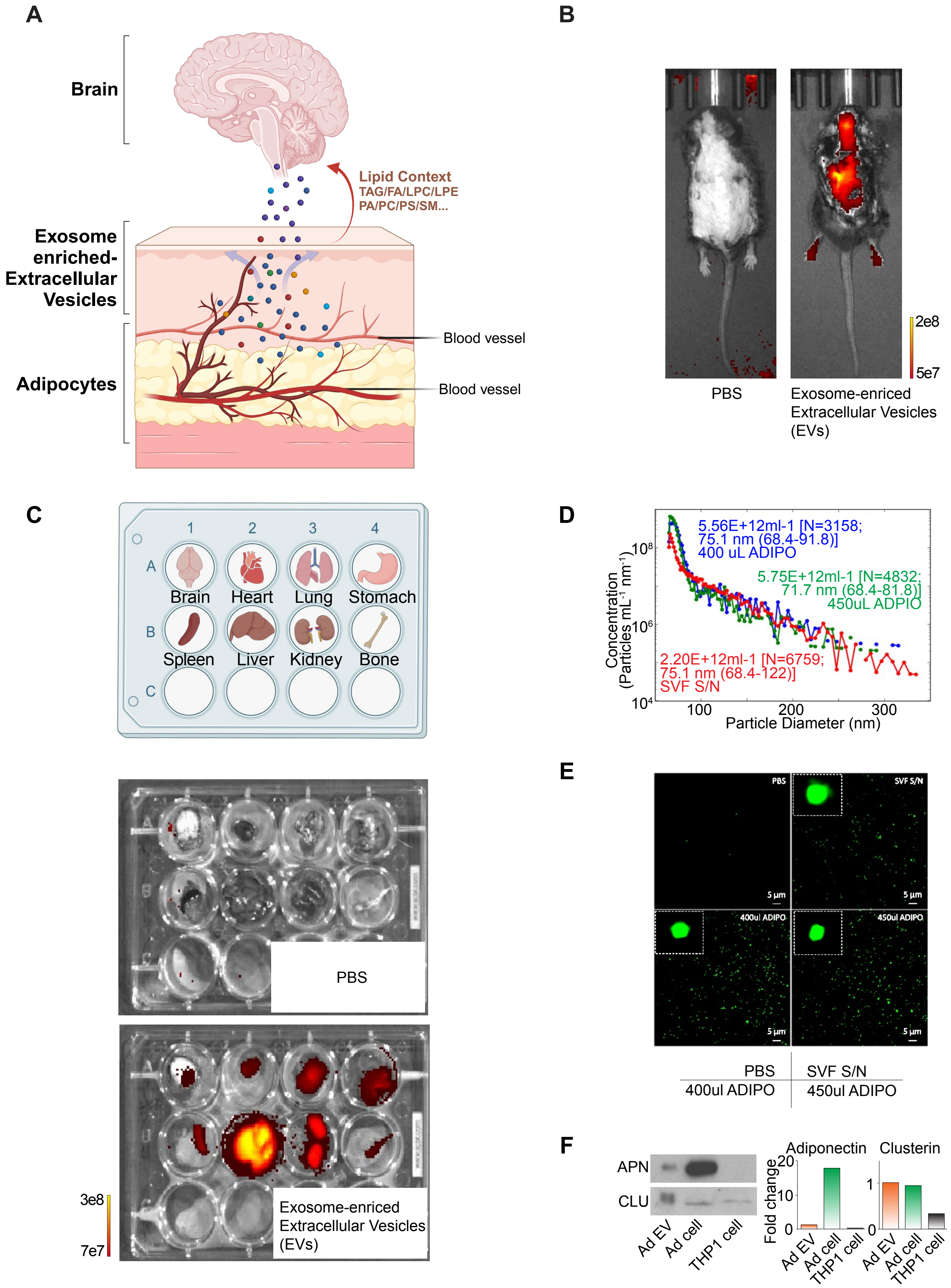
A
Brain
Lipid Context
TAG/FA/LPC/LPE
PA/PC/PS/SM...
Exosome enriched-Extracellular Vesicles
Adipocytes
Blood vessel
Blood vessel
B
PBS
Exosome-enriced Extracellular Vesicles (EVs)
2e8
5e7
C
1
2
3
4
A
B
C
Brain
Heart
Lung
Stomach
Spleen
Liver
Kidney
Bone
PBS
Exosome-enriced Extracellular Vesicles (EVs)
3e8
7e7
D
5.56E+12ml-1 [N=3158; 75.1 nm (68.4-91.8)] 400 uL ADIPO
5.75E+12ml-1 [N=4832; 71.7 nm (68.4-81.8)] 450uL ADPIO
2.20E+12ml-1 [N=6759; 75.1 nm (68.4-122)] SVF S/N
Concentration (Particles mL^{-1} nm^{-1})
10^8
10^6
10^4
100
200
300
Particle Diameter (nm)
E
PBS
SVF S/N
400ul ADIPO
450ul ADIPO
5 µm
PBS
SVF S/N
400ul ADIPO
450ul ADIPO
F
APN
CLU
Ad EV
Ad cell
THP1 cell
Adiponectin
Clusterin
Fold change
20
10
0
1
0
Ad EV
Ad cell
THP1 cell
Ad EV
Ad cell
THP1 cell

Figure S1 Characterization and in vivo distribution of adipocyte-derived EVs.

A. Schematic representation of adipocyte-derived EVs penetrating the brain and mediating crosstalk between adipose tissue and the central nervous system.

B. Biodistribution of isolated EVs in C57bl/6j WT mice with In Vivo Imaging System (IVIS). The fur of the mice was shaved for imaging. The EVs were labeled with ExoGlow™-Vivo EV Labeling Kit.

C. In vivo biodistribution of exosome-enriched EVs assessed by fluorescence imaging 6 hours after injection. Top: Schematic layout of organ placement in a 12-well plate following dissection. Middle: Representative IVIS fluorescence image of organs collected from mice injected with PBS (negative control). Bottom: Representative IVIS image of organs collected from mice intravenously injected with fluorescently labeled EVs.

D. Microfluidic Resistive Pulse Sensing (MRPS) analysis of exosome-enriched EVs derived from human adipocytes.

E. Representative fluorescence images showing CD63-positive EVs detected across different isolation conditions. CD63, a tetraspanin enriched in exosomes, was used to validate EV identity. EVs were stained with a CD63-targeting probe and imaged under the same acquisition parameters. EV signal intensity and particle abundance were elevated in samples isolated from adipocyte-conditioned media (400 µL and 450 µL ADIPO), while minimal signal was observed in the PBS control. Insets highlight individual fluorescent EV-like structures. Scale bars: 5 µm.

F. Western blot and quantification of adiponectin (APN, ~30 kDa) and clusterin (CLU, ~40 kDa) expression in adipocyte-derived EVs and cell lysates. EVs (Ad EV), cultured human subcutaneous adipocytes (Ad cell), and THP-1 monocytes (THP1 cell, negative control) were analyzed. Protein abundance was normalized to total protein levels assessed by Ponceau S staining. Bar graphs show fold change relative to the Ad EV group.

Figure S2

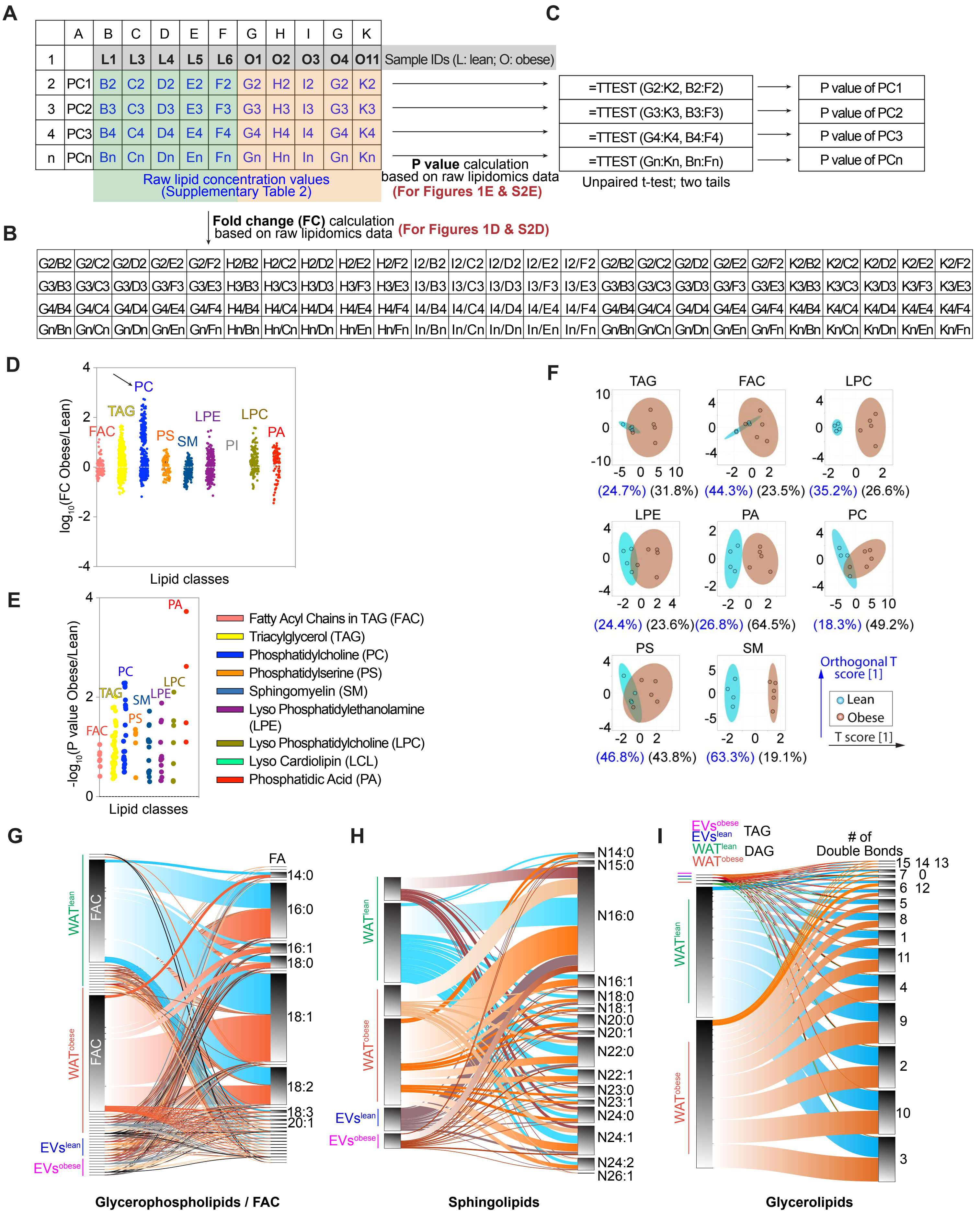

A
Sample IDs (L: lean; O: obese)
L1 L3 L4 L5 L6 O1 O2 O3 O4 O11
PC1 PC2 PC3 PCn
Raw lipid concentration values
(Supplementary Table 2)
P value calculation
based on raw lipidomics data
(For Figures 1E & S2E)
C
=TTEST (G2:K2, B2:F2)
=TTEST (G3:K3, B3:F3)
=TTEST (G4:K4, B4:F4)
=TTEST (Gn:Kn, Bn:Fn)
Unpaired t-test; two tails
P value of PC1
P value of PC2
P value of PC3
P value of PCn
Fold change (FC) calculation
based on raw lipidomics data
(For Figures 1D & S2D)
B
D
log10(FC Obese/Lean)
Lipid classes
FAC TAG PC PS SM LPE PI LPC PA
E
-log10(P value Obese/Lean)
Lipid classes
Fatty Acyl Chains in TAG (FAC)
Triacylglycerol (TAG)
Phosphatidylcholine (PC)
Phosphatidylserine (PS)
Sphingomyelin (SM)
Lyso Phosphatidylethanolamine (LPE)
Lyso Phosphatidylcholine (LPC)
Lyso Cardiolipin (LCL)
Phosphatidic Acid (PA)
F
TAG FAC LPC
(24.7%) (31.8%) (44.3%) (23.5%) (35.2%) (26.6%)
LPE PA PC
(24.4%) (23.6%)(26.8%) (64.5%) (18.3%) (49.2%)
PS SM
(46.8%) (43.8%) (63.3%) (19.1%)
Orthogonal T score [1]
Lean
Obese
T score [1]
G
FA
WATlean WATobese EVslean EVsobese
FAC
14:0 16:0 16:1 18:0 18:1 18:2 18:3 20:1
Glycerophospholipids / FAC
H
N14:0 N15:0 N16:0 N16:1 N18:0 N18:1 N20:0 N20:1 N22:0 N22:1 N23:0 N23:1 N24:0 N24:1 N24:2 N26:1
Sphingolipids
I
TAG
DAG
# of Double Bonds
Glycerolipids

Figure S2 Statistical framework and EV lipidomic alterations between lean and obese individuals.

A. Matrix layout of raw lipid concentration values used for statistical analysis. Columns represent individual samples from lean (L1–L6) and obese (O1–O5, O11) individuals, while rows represent individual lipid species (PC1 to PCn).

B. Fold change (FC) values were calculated as the ratio of lipid abundance between obese and lean individuals across all pairwise combinations (Obese/Lean). This forms the basis for comparative analysis of lipid enrichment between groups (used in Figures 1D and S2D).

C. Unpaired, two-tailed Student's t-tests were performed for each lipid species (e.g., PC1 to PCn) using raw values from lean and obese samples to determine statistical significance (used in Figures 1E and S2E). The example formula shown (e.g., =TTEST(G2:K2, B2:F2)) illustrates how each lipid's p-value was computed across the two groups. Supplementary Table 2 contains the complete raw data matrix used in this analysis.

D-E. Simplified Manhattan plots of lipid class alterations between obese and lean EVs (excluding Lean 1). (**D**) Fold changes ($\log_{10}$[Obese/Lean]) of individual lipid classes stratified by lipid species. Each dot represents a detected lipid species, color-coded by class. (**E**) Statistical significance ($-\log_{10}$ P value) of lipid class differences calculated based on unpaired two-tailed t-tests using raw lipidomic concentrations from lean and obese individuals. Notable classes such as FAC, TAG, PC, LPC, and PA exhibit significant alterations in fold change and/or P value distribution.

F. Partial least squares discriminant analysis (PLS-DA) plots showing lipid class–specific separation between EVs from lean and obese individuals (excluding Lean 1). EV lipidomic data were analyzed with individual patient samples as input. Samples from lean and obese subjects are shown in blue and brown, respectively, with ellipses representing 95% confidence intervals. The percentage of explained variance for the first and second components (T score [1] and orthogonal T score [1]) is indicated in parentheses.

G-I. Glycerophospholipids, sphingolipids, glycerolipids and their associated fatty acyl chains (FAC).

Figure S3

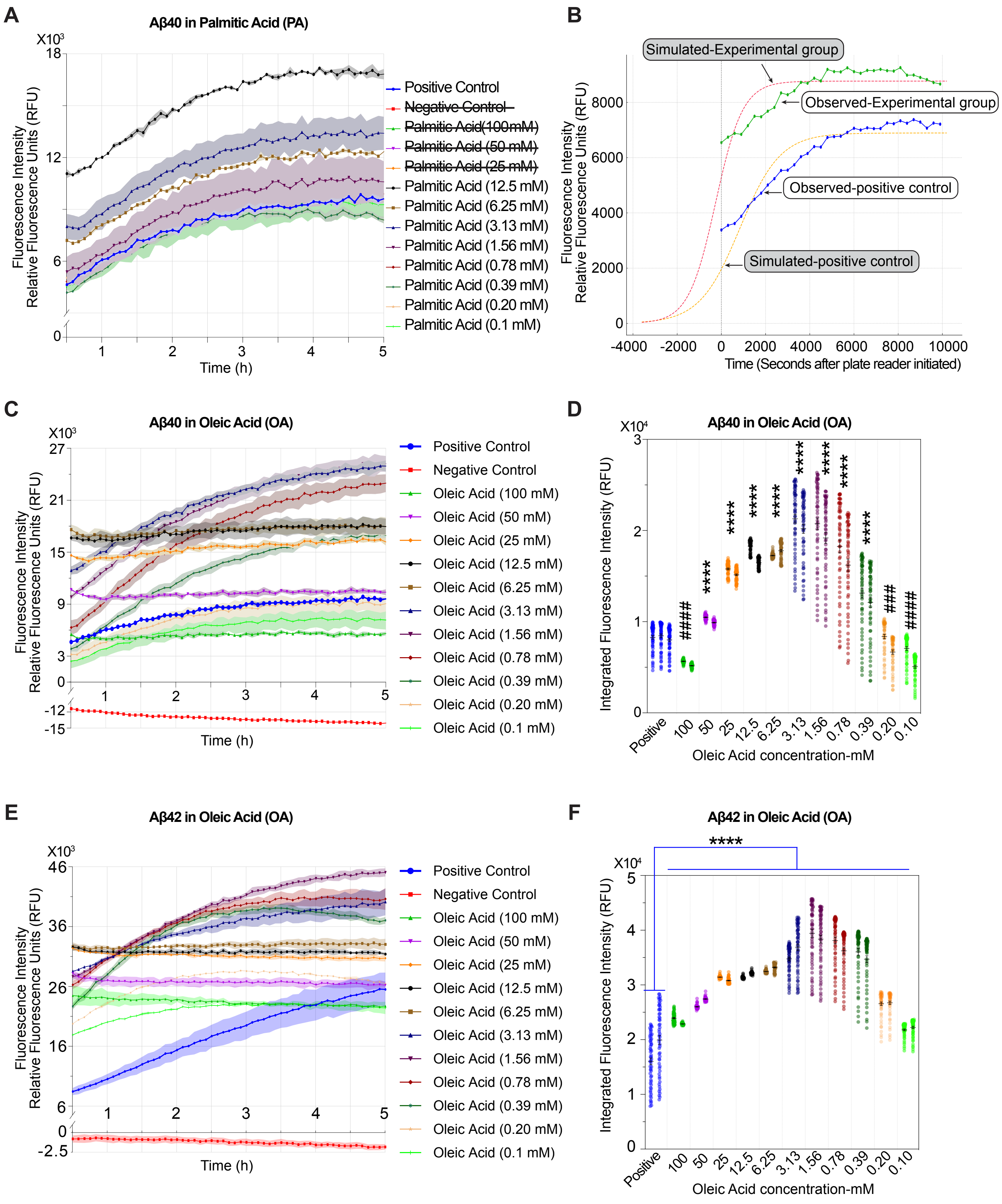
A
Aβ40 in Palmitic Acid (PA)
X10³
Fluorescence Intensity
Relative Fluorescence Units (RFU)
Time (h)
Positive Control
Negative Control
Palmitic Acid(100mM)
Palmitic Acid (50 mM)
Palmitic Acid (25 mM)
Palmitic Acid (12.5 mM)
Palmitic Acid (6.25 mM)
Palmitic Acid (3.13 mM)
Palmitic Acid (1.56 mM)
Palmitic Acid (0.78 mM)
Palmitic Acid (0.39 mM)
Palmitic Acid (0.20 mM)
Palmitic Acid (0.1 mM)
B
Simulated-Experimental group
Observed-Experimental group
Observed-positive control
Simulated-positive control
Time (Seconds after plate reader initiated)
C
Aβ40 in Oleic Acid (OA)
Positive Control
Negative Control
Oleic Acid (100 mM)
Oleic Acid (50 mM)
Oleic Acid (25 mM)
Oleic Acid (12.5 mM)
Oleic Acid (6.25 mM)
Oleic Acid (3.13 mM)
Oleic Acid (1.56 mM)
Oleic Acid (0.78 mM)
Oleic Acid (0.39 mM)
Oleic Acid (0.20 mM)
Oleic Acid (0.1 mM)
D
Aβ40 in Oleic Acid (OA)
X10⁴
Integrated Fluorescence Intensity (RFU)
Oleic Acid concentration-mM
E
Aβ42 in Oleic Acid (OA)
F
Aβ42 in Oleic Acid (OA)

Figure S3 Experimental kinetics of Aβ aggregation in the presence of palmitic acid and oleic acid.

A. Aggregation kinetics of Aβ40 in the presence of serially diluted palmitic acid (0.1–12.5 mM), higher concentrations (25–100 mM) and negative controls were excluded.

B. Comparison of observed and simulated fluorescence kinetics for experimental and control groups, with fluorescence intensity initiated from baseline (RFU = 0). The experimental group (green: observed; red dashed: simulated) and the positive control group (blue: observed; orange dashed: simulated) were presented in parallel. The x-axis represents time in seconds relative to the initiation of plate reader measurement (time zero). Observed traces reflect empirical kinetic data acquired from a 384-well fluorescence assay, while simulated curves represent fitted models capturing the expected fluorescence progression.

C-F. Experimental kinetics for Aβ40 and Aβ42 aggregation under varying concentrations of oleic acid (OA). **(C & E)** Aggregation kinetics of Aβ40 and Aβ42 in the presence of serially diluted oleic acid (0.1–100 mM), using the ThT fluorescence assay. Fluorescence intensity is shown as Relative Fluorescence Units (RFU) at Ex/Em = 440/484 nm. Positive control: Aβ40 or Aβ42 + ThT; negative control: Aβ40 or Aβ42 + ThT + phenol red + morin. **(D & F)** Quantification of aggregation kinetics at varying concentrations. Each dot represents an individual fluorescence measurement (RFU) at a specific time point for the indicated lipid concentration. Bars indicate the mean ± SEM for each group. Asterisks (*) and hash symbols (#) indicate statistical significance compared to the Positive control group. Statistical approach consistent with Methods section 2.8, with significance thresholds as follows: *, increase; $^{\#}$, decrease; P>0.05 (ns), P<0.05 ($^{*/\#}$), P<0.01 ($^{**/\#\#}$), P<0.001 ($^{***/\#\#\#}$), and P< 0.0001 ($^{****/\#\#\#\#}$).

Figure S4

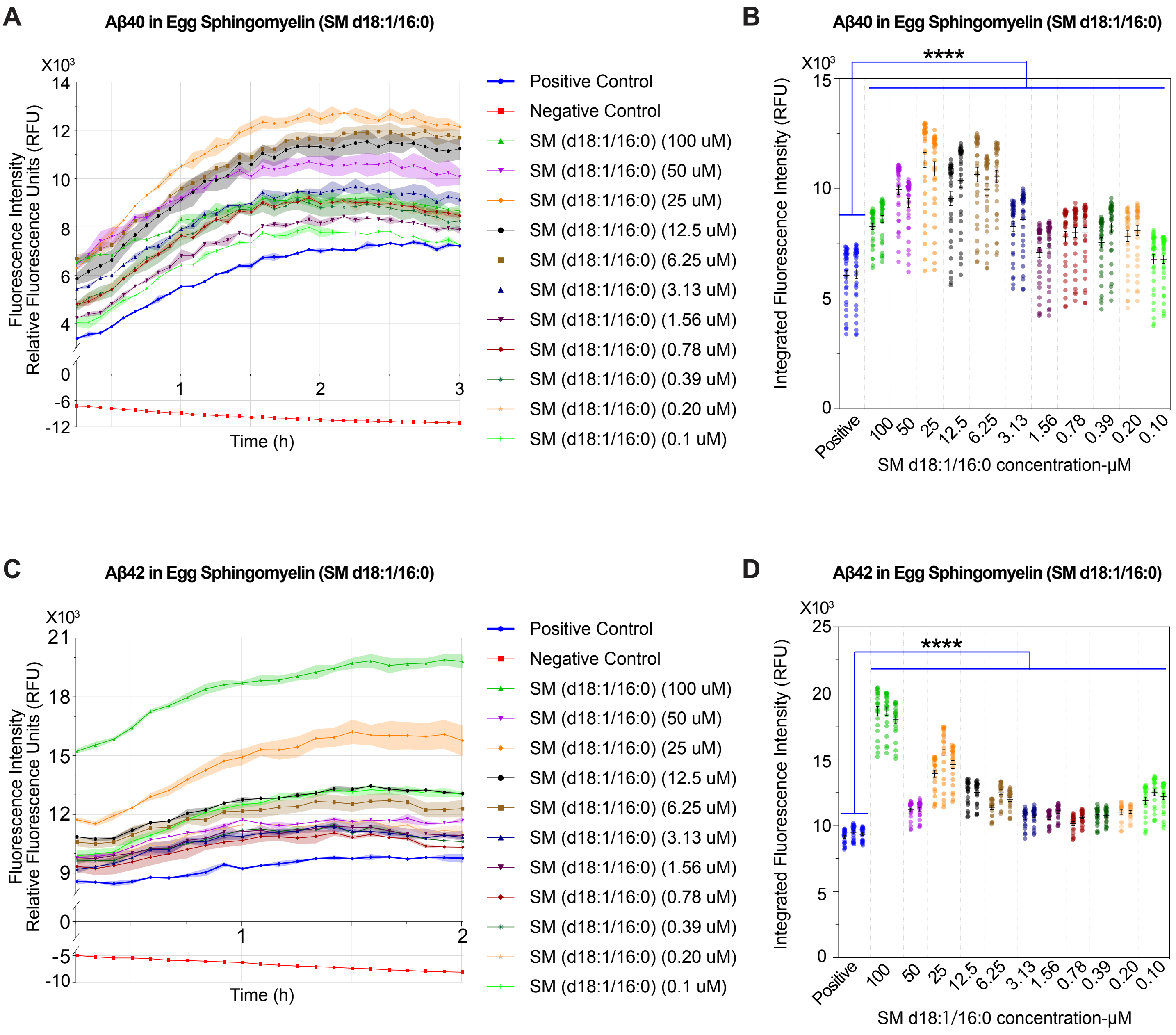
A
Aβ40 in Egg Sphingomyelin (SM d18:1/16:0)
Fluorescence Intensity
Relative Fluorescence Units (RFU)
X10³
Time (h)
Positive Control
Negative Control
SM (d18:1/16:0) (100 uM)
SM (d18:1/16:0) (50 uM)
SM (d18:1/16:0) (25 uM)
SM (d18:1/16:0) (12.5 uM)
SM (d18:1/16:0) (6.25 uM)
SM (d18:1/16:0) (3.13 uM)
SM (d18:1/16:0) (1.56 uM)
SM (d18:1/16:0) (0.78 uM)
SM (d18:1/16:0) (0.39 uM)
SM (d18:1/16:0) (0.20 uM)
SM (d18:1/16:0) (0.1 uM)
B
Aβ40 in Egg Sphingomyelin (SM d18:1/16:0)
Integrated Fluorescence Intensity (RFU)
X10³
****
Positive
100
50
25
12.5
6.25
3.13
1.56
0.78
0.39
0.20
0.10
SM d18:1/16:0 concentration-μM
C
Aβ42 in Egg Sphingomyelin (SM d18:1/16:0)
Fluorescence Intensity
Relative Fluorescence Units (RFU)
X10³
Time (h)
Positive Control
Negative Control
SM (d18:1/16:0) (100 uM)
SM (d18:1/16:0) (50 uM)
SM (d18:1/16:0) (25 uM)
SM (d18:1/16:0) (12.5 uM)
SM (d18:1/16:0) (6.25 uM)
SM (d18:1/16:0) (3.13 uM)
SM (d18:1/16:0) (1.56 uM)
SM (d18:1/16:0) (0.78 uM)
SM (d18:1/16:0) (0.39 uM)
SM (d18:1/16:0) (0.20 uM)
SM (d18:1/16:0) (0.1 uM)
D
Aβ42 in Egg Sphingomyelin (SM d18:1/16:0)
Integrated Fluorescence Intensity (RFU)
X10³
****
Positive
100
50
25
12.5
6.25
3.13
1.56
0.78
0.39
0.20
0.10
SM d18:1/16:0 concentration-μM

Figure S4 Experimental kinetics of Aβ aggregation in the presence of egg sphingomyelin.

A-D. Experimental kinetics for Aβ40 and Aβ42 aggregation under varying concentrations of Egg SM. **(A & C)** Aggregation kinetics of Aβ40 and Aβ42 in the presence of serially diluted Egg SM (0.1–100 mM), using the ThT fluorescence assay. Fluorescence intensity is shown as Relative Fluorescence Units (RFU) at Ex/Em = 440/484 nm. Positive control: Aβ40 or Aβ42 + ThT; negative control: Aβ40 or Aβ42 + ThT + phenol red + morin. **(B & D)** Quantification of aggregation kinetics at varying concentrations. Each dot represents an individual fluorescence measurement (RFU) at a specific time point for the indicated lipid concentration. Bars indicate the mean ± SEM for each group. Asterisks (*) and hash symbols (#) indicate statistical significance compared to the Positive control group. Statistical approach consistent with Methods section 2.8, with significance thresholds as follows: *, increase; $^{\#}$, decrease; P>0.05 (ns), P<0.05 ($^{*/\#}$), P<0.01 ($^{**/\#\#}$), P<0.001 ($^{***/\#\#\#}$), and P< 0.0001 ($^{****/\#\#\#\#}$).

Figure S5

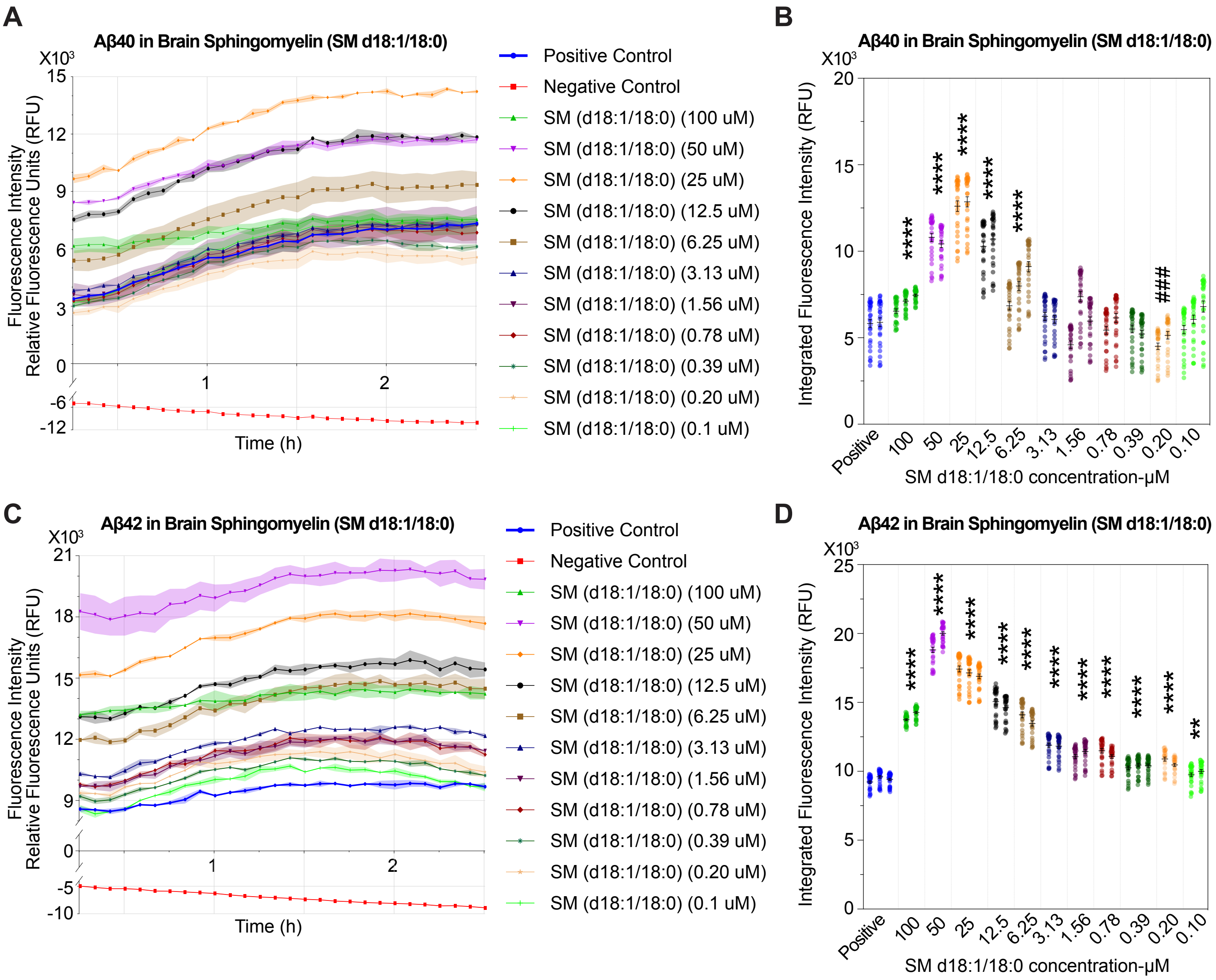
A
Aβ40 in Brain Sphingomyelin (SM d18:1/18:0)
Fluorescence Intensity
Relative Fluorescence Units (RFU)
X10³
Time (h)
Positive Control
Negative Control
SM (d18:1/18:0) (100 uM)
SM (d18:1/18:0) (50 uM)
SM (d18:1/18:0) (25 uM)
SM (d18:1/18:0) (12.5 uM)
SM (d18:1/18:0) (6.25 uM)
SM (d18:1/18:0) (3.13 uM)
SM (d18:1/18:0) (1.56 uM)
SM (d18:1/18:0) (0.78 uM)
SM (d18:1/18:0) (0.39 uM)
SM (d18:1/18:0) (0.20 uM)
SM (d18:1/18:0) (0.1 uM)
B
Aβ40 in Brain Sphingomyelin (SM d18:1/18:0)
Integrated Fluorescence Intensity (RFU)
X10³
Positive
100
50
25
12.5
6.25
3.13
1.56
0.78
0.39
0.20
0.10
****
###
SM d18:1/18:0 concentration-μM
C
Aβ42 in Brain Sphingomyelin (SM d18:1/18:0)
Fluorescence Intensity
Relative Fluorescence Units (RFU)
X10³
Time (h)
Positive Control
Negative Control
SM (d18:1/18:0) (100 uM)
SM (d18:1/18:0) (50 uM)
SM (d18:1/18:0) (25 uM)
SM (d18:1/18:0) (12.5 uM)
SM (d18:1/18:0) (6.25 uM)
SM (d18:1/18:0) (3.13 uM)
SM (d18:1/18:0) (1.56 uM)
SM (d18:1/18:0) (0.78 uM)
SM (d18:1/18:0) (0.39 uM)
SM (d18:1/18:0) (0.20 uM)
SM (d18:1/18:0) (0.1 uM)
D
Aβ42 in Brain Sphingomyelin (SM d18:1/18:0)
Integrated Fluorescence Intensity (RFU)
X10³
Positive
100
50
25
12.5
6.25
3.13
1.56
0.78
0.39
0.20
0.10
****
**
SM d18:1/18:0 concentration-μM

Figure S5 Experimental kinetics of Aβ aggregation in the presence of brain sphingomyelin.

A-D. Experimental kinetics for Aβ40 and Aβ42 aggregation under varying concentrations of Brain SM. **(A & C)** Aggregation kinetics of Aβ40 and Aβ42 in the presence of serially diluted Brain SM (0.1–100 mM), using the ThT fluorescence assay. Fluorescence intensity is shown as Relative Fluorescence Units (RFU) at Ex/Em = 440/484 nm. Positive control: Aβ40 or Aβ42 + ThT; negative control: Aβ40 or Aβ42 + ThT + phenol red + morin. **(B & D)** Quantification of aggregation kinetics at varying concentrations. Each dot represents an individual fluorescence measurement (RFU) at a specific time point for the indicated lipid concentration. Bars indicate the mean ± SEM for each group. Asterisks (*) and hash symbols (#) indicate statistical significance compared to the Positive control group. Statistical approach consistent with Methods section 2.8, with significance thresholds as follows: *, increase; $^{\#}$, decrease; P>0.05 (ns), P<0.05 ($^{*/\#}$), P<0.01 ($^{**/\#\#}$), P<0.001 ($^{***/\#\#\#}$), and P< 0.0001 ($^{****/\#\#\#\#}$).

Figure S6

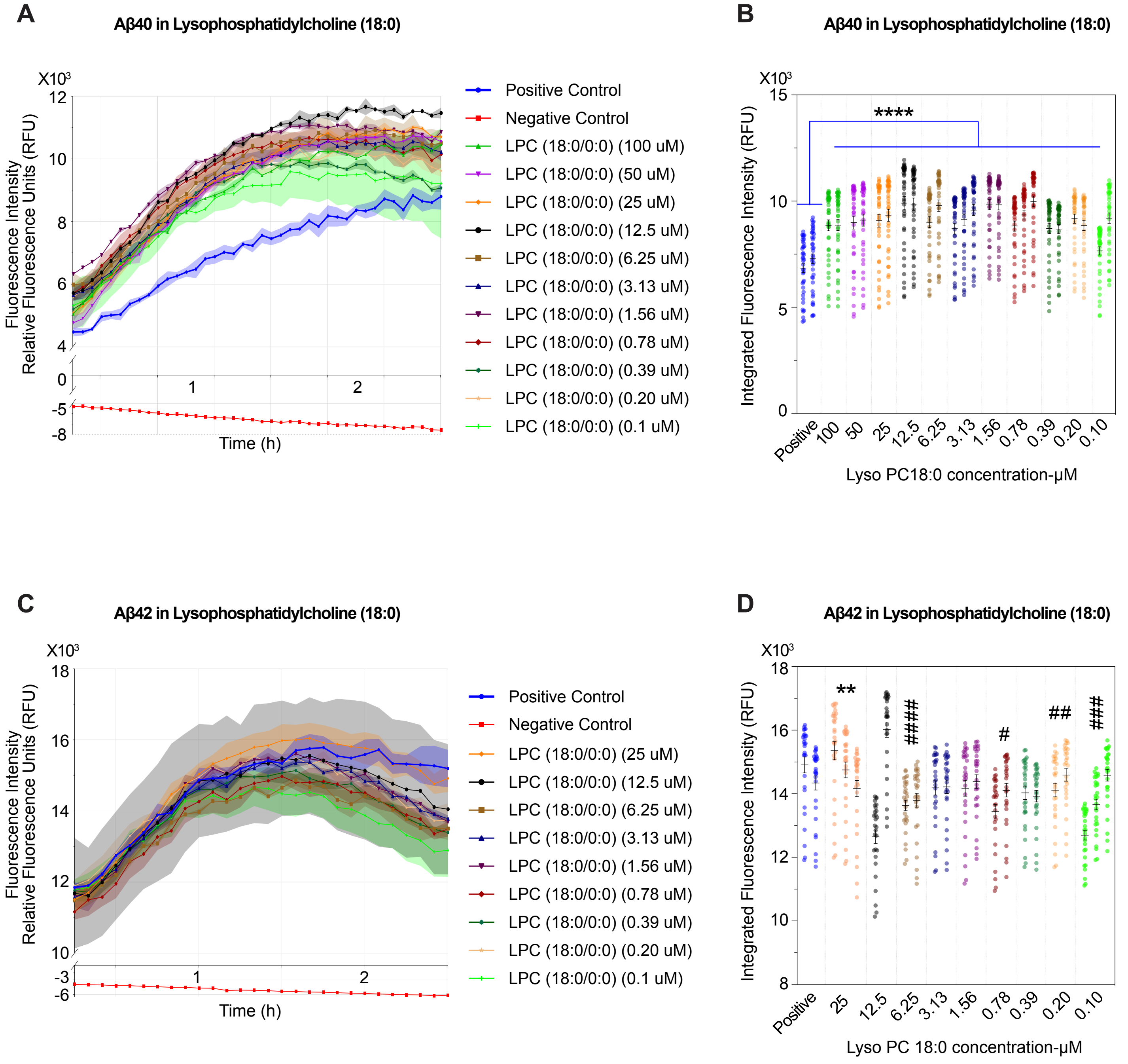
A
Aβ40 in Lysophosphatidylcholine (18:0)
X10³
Fluorescence Intensity
Relative Fluorescence Units (RFU)
12
10
8
6
4
0
-5
-8
1
2
Time (h)
Positive Control
Negative Control
LPC (18:0/0:0) (100 uM)
LPC (18:0/0:0) (50 uM)
LPC (18:0/0:0) (25 uM)
LPC (18:0/0:0) (12.5 uM)
LPC (18:0/0:0) (6.25 uM)
LPC (18:0/0:0) (3.13 uM)
LPC (18:0/0:0) (1.56 uM)
LPC (18:0/0:0) (0.78 uM)
LPC (18:0/0:0) (0.39 uM)
LPC (18:0/0:0) (0.20 uM)
LPC (18:0/0:0) (0.1 uM)
B
Aβ40 in Lysophosphatidylcholine (18:0)
X10³
Integrated Fluorescence Intensity (RFU)
15
10
5
0
****
Positive
100
50
25
12.5
6.25
3.13
1.56
0.78
0.39
0.20
0.10
Lyso PC18:0 concentration-μM
C
Aβ42 in Lysophosphatidylcholine (18:0)
X10³
Fluorescence Intensity
Relative Fluorescence Units (RFU)
18
16
14
12
10
-3
-6
1
2
Time (h)
Positive Control
Negative Control
LPC (18:0/0:0) (25 uM)
LPC (18:0/0:0) (12.5 uM)
LPC (18:0/0:0) (6.25 uM)
LPC (18:0/0:0) (3.13 uM)
LPC (18:0/0:0) (1.56 uM)
LPC (18:0/0:0) (0.78 uM)
LPC (18:0/0:0) (0.39 uM)
LPC (18:0/0:0) (0.20 uM)
LPC (18:0/0:0) (0.1 uM)
D
Aβ42 in Lysophosphatidylcholine (18:0)
X10³
Integrated Fluorescence Intensity (RFU)
18
16
14
12
10
8
**
####
#
##
###
Positive
25
12.5
6.25
3.13
1.56
0.78
0.39
0.20
0.10
Lyso PC 18:0 concentration-μM

Figure S6 Experimental kinetics of Aβ aggregation in the presence of lysophosphatidylcholine 18:0.

A-D. Experimental kinetics for Aβ40 and Aβ42 aggregation under varying concentrations of LPC 18:0. **(A & C)** Aggregation kinetics of Aβ40 and Aβ42 in the presence of serially diluted LPC 18:0 (0.1–100 mM), using the ThT fluorescence assay. Fluorescence intensity is shown as Relative Fluorescence Units (RFU) at Ex/Em = 440/484 nm. Positive control: Aβ40 or Aβ42 + ThT; negative control: Aβ40 or Aβ42 + ThT + phenol red + morin. **(B & D)** Quantification of aggregation kinetics at varying concentrations. Each dot represents an individual fluorescence measurement (RFU) at a specific time point for the indicated lipid concentration. Bars indicate the mean ± SEM for each group. Asterisks (*) and hash symbols (#) indicate statistical significance compared to the Positive control group. Statistical approach consistent with Methods section 2.8, with significance thresholds as follows: *, increase; $^{\#}$, decrease; P>0.05 (ns), P<0.05 ($^{*/\#}$), P<0.01 ($^{**/\#\#}$), P<0.001 ($^{***/\#\#\#}$), and P< 0.0001 ($^{****/\#\#\#\#}$).

Figure S7

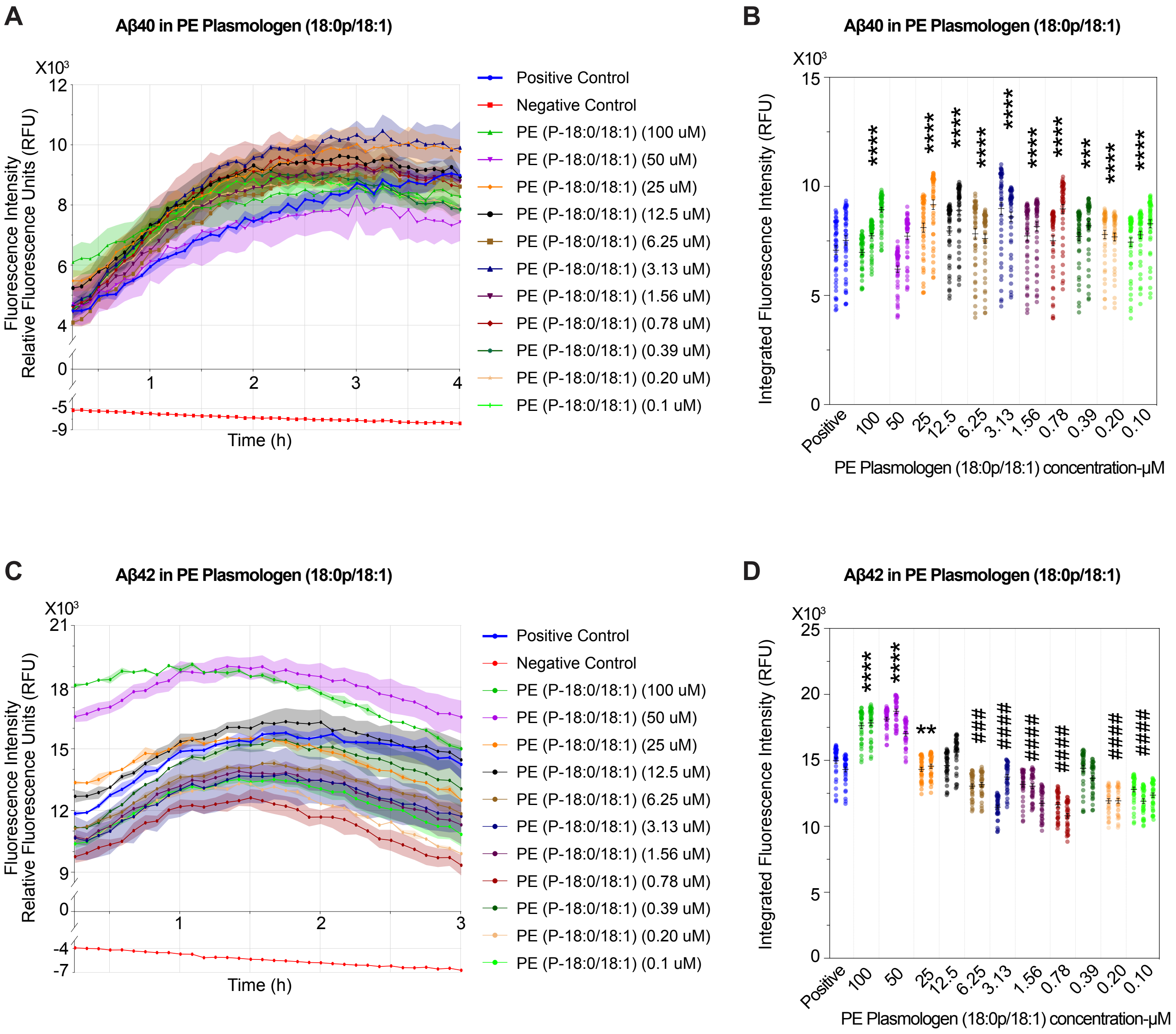
A
Aβ40 in PE Plasmologen (18:0p/18:1)
X10³
Fluorescence Intensity
Relative Fluorescence Units (RFU)
Time (h)
Positive Control
Negative Control
PE (P-18:0/18:1) (100 uM)
PE (P-18:0/18:1) (50 uM)
PE (P-18:0/18:1) (25 uM)
PE (P-18:0/18:1) (12.5 uM)
PE (P-18:0/18:1) (6.25 uM)
PE (P-18:0/18:1) (3.13 uM)
PE (P-18:0/18:1) (1.56 uM)
PE (P-18:0/18:1) (0.78 uM)
PE (P-18:0/18:1) (0.39 uM)
PE (P-18:0/18:1) (0.20 uM)
PE (P-18:0/18:1) (0.1 uM)
B
Aβ40 in PE Plasmologen (18:0p/18:1)
X10³
Integrated Fluorescence Intensity (RFU)
Positive
100
50
25
12.5
6.25
3.13
1.56
0.78
0.39
0.20
0.10
PE Plasmologen (18:0p/18:1) concentration-μM
C
Aβ42 in PE Plasmologen (18:0p/18:1)
X10³
Fluorescence Intensity
Relative Fluorescence Units (RFU)
Time (h)
Positive Control
Negative Control
PE (P-18:0/18:1) (100 uM)
PE (P-18:0/18:1) (50 uM)
PE (P-18:0/18:1) (25 uM)
PE (P-18:0/18:1) (12.5 uM)
PE (P-18:0/18:1) (6.25 uM)
PE (P-18:0/18:1) (3.13 uM)
PE (P-18:0/18:1) (1.56 uM)
PE (P-18:0/18:1) (0.78 uM)
PE (P-18:0/18:1) (0.39 uM)
PE (P-18:0/18:1) (0.20 uM)
PE (P-18:0/18:1) (0.1 uM)
D
Aβ42 in PE Plasmologen (18:0p/18:1)
X10³
Integrated Fluorescence Intensity (RFU)
Positive
100
50
25
12.5
6.25
3.13
1.56
0.78
0.39
0.20
0.10
PE Plasmologen (18:0p/18:1) concentration-μM

Figure S7 Experimental kinetics of Aβ aggregation in the presence of C18(Plasm)-18:1 PE.

A-D. Experimental kinetics for Aβ40 and Aβ42 aggregation under varying concentrations of C18(Plasm)-18:1 PE. **(A & C)** Aggregation kinetics of Aβ40 and Aβ42 in the presence of serially diluted C18(Plasm)-18:1 PE (0.1–100 mM), using the ThT fluorescence assay. Fluorescence intensity is shown as Relative Fluorescence Units (RFU) at Ex/Em = 440/484 nm. Positive control: Aβ40 or Aβ42 + ThT; negative control: Aβ40 or Aβ42 + ThT + phenol red + morin. **(B & D)** Quantification of aggregation kinetics at varying concentrations. Each dot represents an individual fluorescence measurement (RFU) at a specific time point for the indicated lipid concentration. Bars indicate the mean ± SEM for each group. Asterisks (*) and hash symbols (#) indicate statistical significance compared to the Positive control group. Statistical approach consistent with Methods section 2.8, with significance thresholds as follows: *, increase; $^{\#}$, decrease; $P>0.05$ (ns), $P<0.05$ ($^{*/\#}$), $P<0.01$ ($^{**/\#\#}$), $P<0.001$ ($^{***/\#\#\#}$), and $P< 0.0001$ ($^{****/\#\#\#\#}$).

Figure S8

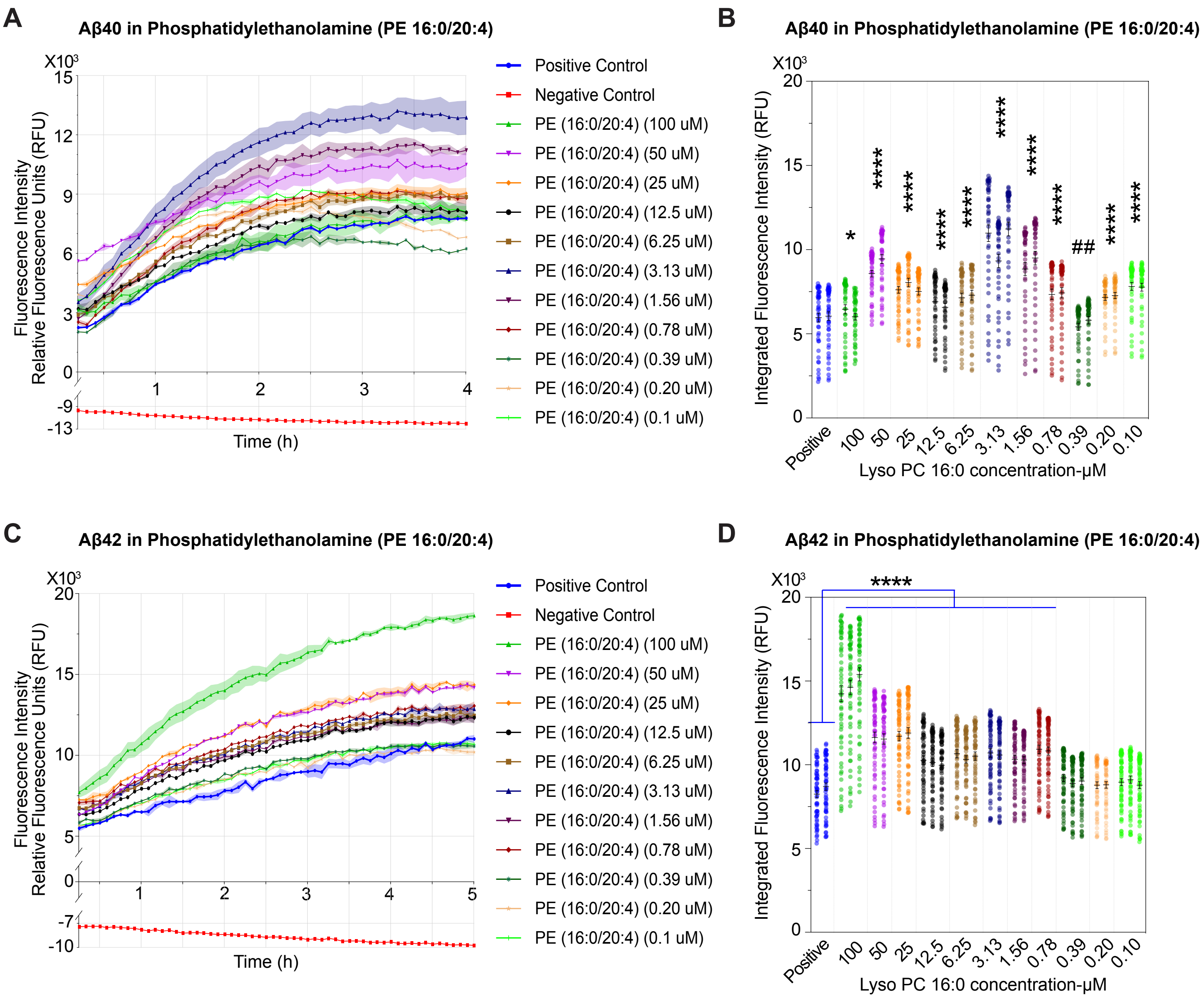

A
Aβ40 in Phosphatidylethanolamine (PE 16:0/20:4)
X10³
Fluorescence Intensity
Relative Fluorescence Units (RFU)
Time (h)
Positive Control
Negative Control
PE (16:0/20:4) (100 uM)
PE (16:0/20:4) (50 uM)
PE (16:0/20:4) (25 uM)
PE (16:0/20:4) (12.5 uM)
PE (16:0/20:4) (6.25 uM)
PE (16:0/20:4) (3.13 uM)
PE (16:0/20:4) (1.56 uM)
PE (16:0/20:4) (0.78 uM)
PE (16:0/20:4) (0.39 uM)
PE (16:0/20:4) (0.20 uM)
PE (16:0/20:4) (0.1 uM)
B
Aβ40 in Phosphatidylethanolamine (PE 16:0/20:4)
X10³
Integrated Fluorescence Intensity (RFU)
Positive
100
50
25
12.5
6.25
3.13
1.56
0.78
0.39
0.20
0.10
Lyso PC 16:0 concentration-μM
*
****
##
C
Aβ42 in Phosphatidylethanolamine (PE 16:0/20:4)
X10³
Fluorescence Intensity
Relative Fluorescence Units (RFU)
Time (h)
Positive Control
Negative Control
PE (16:0/20:4) (100 uM)
PE (16:0/20:4) (50 uM)
PE (16:0/20:4) (25 uM)
PE (16:0/20:4) (12.5 uM)
PE (16:0/20:4) (6.25 uM)
PE (16:0/20:4) (3.13 uM)
PE (16:0/20:4) (1.56 uM)
PE (16:0/20:4) (0.78 uM)
PE (16:0/20:4) (0.39 uM)
PE (16:0/20:4) (0.20 uM)
PE (16:0/20:4) (0.1 uM)
D
Aβ42 in Phosphatidylethanolamine (PE 16:0/20:4)
X10³
****
Integrated Fluorescence Intensity (RFU)
Positive
100
50
25
12.5
6.25
3.13
1.56
0.78
0.39
0.20
0.10
Lyso PC 16:0 concentration-μM

Figure S8 Experimental kinetics of Aβ aggregation in the presence of 16:0-20:4 PE.

A-D. Experimental kinetics for Aβ40 and Aβ42 aggregation under varying concentrations of 16:0-20:4 PE. **(A & C)** Aggregation kinetics of Aβ40 and Aβ42 in the presence of serially diluted 16:0-20:4 PE (0.1–100 mM), using the ThT fluorescence assay. Fluorescence intensity is shown as Relative Fluorescence Units (RFU) at Ex/Em = 440/484 nm. Positive control: Aβ40 or Aβ42 + ThT; negative control: Aβ40 or Aβ42 + ThT + phenol red + morin. **(B & D)** Quantification of aggregation kinetics at varying concentrations. Each dot represents an individual fluorescence measurement (RFU) at a specific time point for the indicated lipid concentration. Bars indicate the mean ± SEM for each group. Asterisks (*) and hash symbols (#) indicate statistical significance compared to the Positive control group. Statistical approach consistent with Methods section 2.8, with significance thresholds as follows: *, increase; $^{\#}$, decrease; $P>0.05$ (ns), $P<0.05$ ($^{*/\#}$), $P<0.01$ ($^{**/\#\#}$), $P<0.001$ ($^{***/\#\#\#}$), and $P< 0.0001$ ($^{****/\#\#\#\#}$).

Figure S9

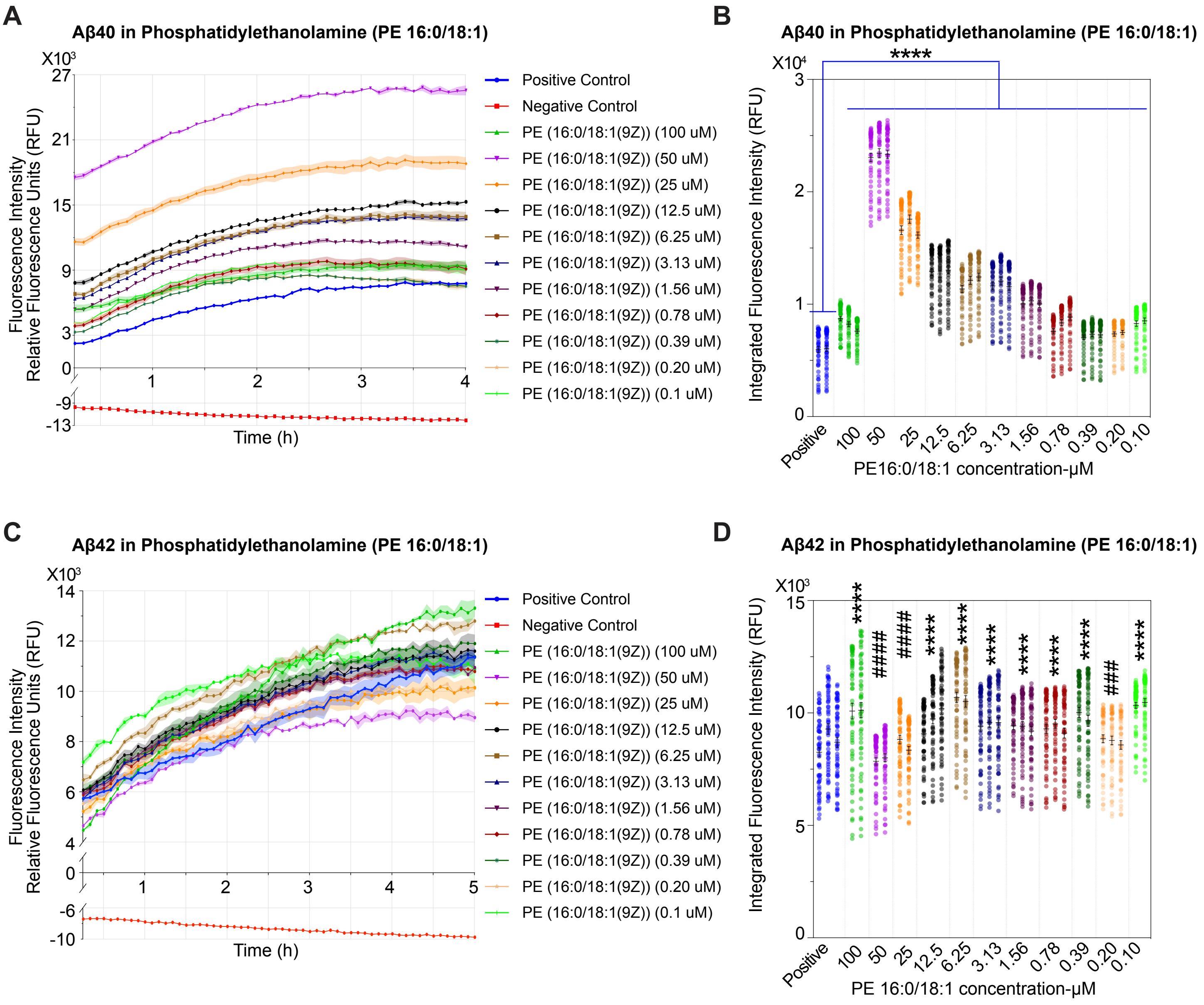
A
Aβ40 in Phosphatidylethanolamine (PE 16:0/18:1)
X10³
Fluorescence Intensity
Relative Fluorescence Units (RFU)
Time (h)
Positive Control
Negative Control
PE (16:0/18:1(9Z)) (100 uM)
PE (16:0/18:1(9Z)) (50 uM)
PE (16:0/18:1(9Z)) (25 uM)
PE (16:0/18:1(9Z)) (12.5 uM)
PE (16:0/18:1(9Z)) (6.25 uM)
PE (16:0/18:1(9Z)) (3.13 uM)
PE (16:0/18:1(9Z)) (1.56 uM)
PE (16:0/18:1(9Z)) (0.78 uM)
PE (16:0/18:1(9Z)) (0.39 uM)
PE (16:0/18:1(9Z)) (0.20 uM)
PE (16:0/18:1(9Z)) (0.1 uM)
B
Aβ40 in Phosphatidylethanolamine (PE 16:0/18:1)
X10⁴
****
Integrated Fluorescence Intensity (RFU)
Positive
100
50
25
12.5
6.25
3.13
1.56
0.78
0.39
0.20
0.10
PE16:0/18:1 concentration-μM
C
Aβ42 in Phosphatidylethanolamine (PE 16:0/18:1)
X10³
Fluorescence Intensity
Relative Fluorescence Units (RFU)
Time (h)
Positive Control
Negative Control
PE (16:0/18:1(9Z)) (100 uM)
PE (16:0/18:1(9Z)) (50 uM)
PE (16:0/18:1(9Z)) (25 uM)
PE (16:0/18:1(9Z)) (12.5 uM)
PE (16:0/18:1(9Z)) (6.25 uM)
PE (16:0/18:1(9Z)) (3.13 uM)
PE (16:0/18:1(9Z)) (1.56 uM)
PE (16:0/18:1(9Z)) (0.78 uM)
PE (16:0/18:1(9Z)) (0.39 uM)
PE (16:0/18:1(9Z)) (0.20 uM)
PE (16:0/18:1(9Z)) (0.1 uM)
D
Aβ42 in Phosphatidylethanolamine (PE 16:0/18:1)
X10³
Integrated Fluorescence Intensity (RFU)
****
####
####
****
****
****
****
****
****
###
****
Positive
100
50
25
12.5
6.25
3.13
1.56
0.78
0.39
0.20
0.10
PE 16:0/18:1 concentration-μM

Figure S9 Experimental kinetics of Aβ aggregation in the presence of 16:0-18:1 PE.

A-D. Experimental kinetics for Aβ40 and Aβ42 aggregation under varying concentrations of 16:0-18:1 PE. **(A & C)** Aggregation kinetics of Aβ40 and Aβ42 in the presence of serially diluted 16:0-18:1 PE (0.1–100 mM), using the ThT fluorescence assay. Fluorescence intensity is shown as Relative Fluorescence Units (RFU) at Ex/Em = 440/484 nm. Positive control: Aβ40 or Aβ42 + ThT; negative control: Aβ40 or Aβ42 + ThT + phenol red + morin. **(B & D)** Quantification of aggregation kinetics at varying concentrations. Each dot represents an individual fluorescence measurement (RFU) at a specific time point for the indicated lipid concentration. Bars indicate the mean ± SEM for each group. Asterisks (*) and hash symbols (#) indicate statistical significance compared to the Positive control group. Statistical approach consistent with Methods section 2.8, with significance thresholds as follows: *, increase; $^{\#}$, decrease; $P>0.05$ (ns), $P<0.05$ ($^{*/\#}$), $P<0.01$ ($^{**/\#\#}$), $P<0.001$ ($^{***/\#\#\#}$), and $P< 0.0001$ ($^{****/\#\#\#\#}$).

Supplementary Table 1. Phenotypic parameters of the total subject population for the EV lipidomics study, including patient ID, age, gender, and BMI.

Group (EV sample description)	EV sample ID	Age (years)	Sex	BMI (kg/m^2)
Human lean 1	L1121	60	Female	25.9
Human lean 3	L1284	68	Female	23.3
Human lean 4	L1285	22	Male	27.2
Human lean 5	L1286	42	Female	20.2
Human lean 6	L1302	71	Male	25.9
Human obese 1	1050	25	Female	48.2
Human obses 2	1055	48	Female	40.7
Human obese 3	1132	35	Female	44.3
Human obese 4	1078	47	Female	41
Human obese 11	1279	57	Female	49.5

Supplementary Table 2. Lipidomics result of human adipocyte-derived EVs normalized to the protein content.

		MASS (*m/z*)	Molecular Weight	Human lean 1	Human lean 3	Human lean 4	Human lean 5	Human lean 6	Human obese 1	Human obese 2	Human obese 3	Human obese 4	Human obese 11
'Triacylglycerol (TAG) (nmol/mg protein)	C48:0/C49:7	813.7523409	806.7363409	2.241225153	0.479083727	0.656263044	2.983804223	2.00078688	0.292400516	0.562900831	0.280663609	0.321335582	1.259281466
'Triacylglycerol (TAG) (nmol/mg protein)	C48:1	811.7366908	804.7206908	1.955043218	1.069851031	0.983519272	1.274763207	1.152128174	0.314861393	1.743280968	0.493475973	0.773599513	1.033036325
'Triacylglycerol (TAG) (nmol/mg protein)	C48:2	809.7210408	802.7050408	1.328159416	0.679715116	0.560463381	0.661550832	0.614723101	0.232346212	2.407291975	0.447773904	0.520150896	0.534353181
'Triacylglycerol (TAG) (nmol/mg protein)	C48:3	807.7053907	800.6893907	0.709319455	0.171008707	0.15280255	0.206322961	0.148030357	0.163593117	0.65724072	0.22678781	0.162959204	0.129780361
'Triacylglycerol (TAG) (nmol/mg protein)	C50:0/C51:7	841.783641	834.767641	1.880056882	0.26086105	0.43473738	2.01890457	1.158670281	0.168746319	0.490232833	0.2681214	0.229004031	0.850669793
'Triacylglycerol (TAG) (nmol/mg protein)	C50:1/C51:8	839.7679909	832.7519909	3.145493159	1.627041226	1.714062358	1.904517977	2.364682655	0.404286174	0.909639651	0.702716594	1.414116601	1.816677814
'Triacylglycerol (TAG) (nmol/mg protein)	C50:2/C51:9	837.7523409	830.7363409	3.465080004	2.104195677	1.937155354	1.546582884	2.242319149	0.507263867	2.001313404	1.332712799	1.986261746	2.003685629
'Triacylglycerol (TAG) (nmol/mg protein)	C50:3/C51:10	835.7366908	828.7206908	1.560087093	0.902252916	0.761082716	0.580435061	0.808268615	0.28314736	1.022319279	0.852400409	0.823514649	0.708409075
'Triacylglycerol (TAG) (nmol/mg protein)	C50:4	833.7210408	826.7050408	0.648546736	0.179570416	0.157207982	0.131783068	0.145202583	0.104859564	0.599697323	0.218790604	0.134367181	0.120189739
'Triacylglycerol (TAG) (nmol/mg protein)	C52:0/C53:7	869.8149411	862.7989411	1.322670737	0.101082126	0.18560687	0.813994827	0.360560609	0.297899613	0.315431498	0.275975734	0.190821989	0.293161692
'Triacylglycerol (TAG) (nmol/mg protein)	C52:1/C53:8	867.7992911	860.7832911	4.447743937	0.669218275	0.844031812	1.097508311	1.003971277	0.259231062	0.889960742	0.299832304	0.732817229	0.809156668
'Triacylglycerol (TAG) (nmol/mg protein)	C52:2/C53:9	865.783641	858.767641	7.015160134	4.544139387	3.753333038	2.862722967	5.124190563	1.264114128	5.364744064	2.414017101	4.868029038	4.012264383
'Triacylglycerol (TAG) (nmol/mg protein)	C52:3/C53:10	863.7679909	856.7519909	5.447689492	3.828618315	2.950546223	1.951991811	3.805732333	1.315173606	2.287608542	2.920875564	4.069035966	2.890279367
'Triacylglycerol (TAG) (nmol/mg protein)	C52:4/C53:11	861.7523409	854.7363409	2.048481625	1.218882773	0.919818635	0.559366613	1.099865086	0.4578856	1.138144468	1.453186764	1.378363818	0.755369047
'Triacylglycerol (TAG) (nmol/mg protein)	C52:5	859.7366908	852.7206908	0.579694766	0.202470674	0.185831177	0.171684542	0.179080974	0.139355743	0.649461364	0.370737189	0.201801865	0.132914523
'Triacylglycerol (TAG) (nmol/mg protein)	C53:0/C54:7	883.7366908	876.7206908	0.536837136	0.039529696	0.063644266	0.1032529	0.055471721	0.092381659	1.281108084	0.159504663	0.182689648	0.054443045
'Triacylglycerol (TAG) (nmol/mg protein)	C53:1/C54:8	881.7210408	874.7050408	1.095869624	0.122842151	0.139164275	0.135922513	0.141859743	0.141662334	0.687580882	0.216061079	1.101942077	0.154552124
'Triacylglycerol (TAG) (nmol/mg protein)	C53:2/C54:9	879.7053907	872.6893907	0.762484463	0.175396158	0.153140084	0.126001633	0.196401344	0.249519937	0.363779087	0.295167621	1.118400468	0.195955951
'Triacylglycerol (TAG) (nmol/mg protein)	C53:3/C54:10	877.783641	870.767641	0.96185655	0.165061053	0.132546704	0.117135499	0.164122708	0.230252123	0.334674799	0.369499765	1.029246912	0.177222716
'Triacylglycerol (TAG) (nmol/mg protein)	C53:4/C54:11	875.7679909	868.7519909	0.849712955	0.034506901	0.043251144	0.050719089	0.028733866	0.141196524	0.314269275	0.147885654	0.232148635	0.048083168
'Triacylglycerol (TAG) (nmol/mg protein)	C54:0/C55:7	897.8462413	890.8302413	1.473229191	0.067339804	0.115547138	0.353128633	0.104083631	0.146632343	0.551651422	0.37244156	0.259732176	0.108301925
'Triacylglycerol (TAG) (nmol/mg protein)	C54:1/C55:8	895.8305912	888.8145912	0.661069514	0.100476007	0.141019177	0.240827257	0.24124929	0.123626119	0.296266258	0.191026454	0.672638705	0.225458259
'Triacylglycerol (TAG) (nmol/mg protein)	C54:2/C55:9	893.8149411	886.7989411	2.070003103	0.799183693	0.811396291	0.707928729	1.23050089	0.172841311	0.916069321	0.569382911	1.413997755	0.917308319
'Triacylglycerol (TAG) (nmol/mg protein)	C54:3/C55:10	891.7992911	884.7832911	4.226822533	2.437904126	2.048187168	1.662921193	3.224340811	0.662351786	1.321973241	1.201538208	2.496149329	2.184453406
'Triacylglycerol (TAG) (nmol/mg protein)	C54:4/C55:11	889.783641	882.767641	2.682733546	1.857322037	1.416894348	0.943101536	2.242257248	0.680528266	1.127904844	1.494041789	1.90817734	1.392872632
'Triacylglycerol (TAG) (nmol/mg protein)	C54:5/C55:12	887.7679909	880.7519909	1.29582158	0.727399136	0.594208504	0.45840261	0.815291389	0.291274697	0.730660956	0.901531415	0.679860562	0.449399049
'Triacylglycerol (TAG) (nmol/mg protein)	C54:6	885.7523409	878.7363409	0.525917782	0.179674085	0.187352081	0.189424311	0.195852669	0.130004068	0.47659805	0.42171074	0.179041796	0.09770018
'Triacylglycerol (TAG) (nmol/mg protein)	C55:2/C56:9	907.7366908	900.7206908	0.533649333	0.066253634	0.070806511	0.090579964	0.104031619	0.121155007	0.234466954	0.236898644	0.587190897	0.106931208
'Triacylglycerol (TAG) (nmol/mg protein)	C55:3/C56:10	905.7210408	898.7050408	0.539938479	0.075744664	0.084215938	0.102442937	0.106036198	0.055455456	0.238046593	0.162723334	0.518352061	0.10567513
'Triacylglycerol (TAG) (nmol/mg protein)	C55:4/C56:11	903.7992911	896.7832911	0.434995466	0.053151544	0.061469857	0.043019892	0.069904863	0.133350427	0.208232142	0.208498159	0.446412876	0.083601903
'Triacylglycerol (TAG) (nmol/mg protein)	C55:5/C56:12	901.783641	894.767641	0.173414365	0.018939233	0.020085805	0.029491778	0.01195038	0.095792223	0.225343478	0.105598708	0.154370992	0.018068756
'Triacylglycerol (TAG) (nmol/mg protein)	C55:6	899.7679909	892.7519909	0.292260604	0.005283377	0.015547658	0.029558294	0.005006376	0.066794795	0.235296193	0.218271276	0.042301783	0.009082252
'Triacylglycerol (TAG) (nmol/mg protein)	C58:0/C59:7/C60:14	953.8149411	946.7989411	0.184888113	0.011684629	0.028011278	0.076547241	0.005154346	0.084648919	0.071164276	0.07324815	0.077476631	0.034100528
'Triacylglycerol (TAG) (nmol/mg protein)	C58:1/C59:8/C60:15	951.8931915	944.8771915	0.139888146	0.030363207	0.022567693	0.044794029	0.004329885	0.176948653	0.111870771	0.140629975	0.106999897	0.027592797
'Triacylglycerol (TAG) (nmol/mg protein)	C58:2/C59:9	949.8775414	942.8615414	0.19721381	0.015803757	0.013170802	0.087303189	0.007123648	0.079973517	0.12018608	0.250242226	0.146082024	0.038394396
'Triacylglycerol (TAG) (nmol/mg protein)	C58:3/C59:10	947.8618913	940.8458913	2.106193685	0.206979536	0.276912392	0.558819329	0.123275315	0.205037776	0.548343036	0.299013429	1.163081133	0.365209532
'Triacylglycerol (TAG) (nmol/mg protein)	C59:5/C60:12	957.7523409	950.7363409	0.087078901	0.011660342	0.004461473	0.019985422	0.006779244	0.040559266	0.035861018	0.046364965	0.01238415	0.011066862
'Triacylglycerol (TAG) (nmol/mg protein)	C59:6/C60:13	955.8305912	948.8145912	0.074366937	0.011443712	0.012670903	0.014772256	0.008170634	0.059777011	0.022241316	0.190955675	0.021221876	0.00444743
Fatty Acyl Chains in TAG (FA) (nmol/mg protein)	228(14:0)	228	228	10.36490124	3.8724311	3.703100282	6.521173286	4.313479649	2.483851467	5.55957075	3.143975591	3.007120045	3.980973769
Fatty Acyl Chains in TAG (FA) (nmol/mg protein)	254(16:1)	254	254	11.04002083	4.916831598	4.018788616	4.598884366	3.831882121	3.668599651	7.420551987	4.939572904	5.186833632	4.11745284
Fatty Acyl Chains in TAG (FA) (nmol/mg protein)	256(16:0)	256	256	48.47312091	22.56662563	21.32030957	30.83968176	31.70670574	11.74101314	26.27081064	16.73025146	27.94212204	24.41410864
Fatty Acyl Chains in TAG (FA) (nmol/mg protein)	278(18:3)	278	278	42.1510934	2.859204119	4.231206948	7.441702201	2.185762791	3.96429216	28.41366085	9.625847842	14.07586067	3.907273988
Fatty Acyl Chains in TAG (FA) (nmol/mg protein)	280(18:2)	280	280	19.08510049	10.17815794	8.49494393	6.809684498	10.85081205	4.692049298	10.21179034	12.01506875	14.01941741	7.511906743
Fatty Acyl Chains in TAG (FA) (nmol/mg protein)	282(18:1)	282	282	56.14892194	32.06809767	27.10483337	23.16111631	38.71961215	10.91547846	18.88785955	20.92360358	40.34147727	28.95898627
Fatty Acyl Chains in TAG (FA) (nmol/mg protein)	284(18:0)	284	284	25.23908714	5.670997449	6.523612173	11.935456	9.047529889	4.093648467	10.88101517	6.811142963	6.441466883	6.940608389
Lyso Phosphatidylcholine (LPC) **(pmol/mg protein)**	16:0	502.3479409	495.3319409	53.85075805	190.2188828	195.7399908	322.2691974	218.2608558	363.1796386	113.3015301	261.1382406	262.0734728	175.7989024
Lyso Phosphatidylcholine (LPC) **(pmol/mg protein)**	18:0	530.379241	523.363241	158.9443588	118.5642975	127.8315708	444.9809291	190.5496002	208.8842403	175.6550379	185.650124	411.9064375	130.075838
Lyso Phosphatidylcholine (LPC) **(pmol/mg protein)**	18:1	528.363591	521.347591	24.16922213	21.71157588	52.92899782	83.20608317	66.48297189	150.6307339	46.27782653	101.7192251	81.43237967	76.79678817
Lyso Phosphatidylcholine (LPC) **(pmol/mg protein)**	18:2	526.3479409	519.3319409	20.23363848	48.32313954	72.21965463	55.32660872	63.51051539	79.72151379	10.06217989	44.93698583	50.0341185	58.04313011
Lyso Phosphatidylcholine (LPC) **(pmol/mg protein)**	A16:0	488.3686763	481.3526763	12.59695504	3.314923744	0.85951518	8.779123063	7.918657152	4.39753337	20.1301037	33.28085316	31.72488651	8.490296973
Lyso Phosphatidylcholine (LPC) **(pmol/mg protein)**	P16:0	486.3530263	479.3370263	46.81983836	7.579404282	16.06918233	12.62686998	17.84418564	10.26453864	77.84227423	83.56007314	44.08557573	30.99388258
Lyso Phosphatidylcholine (LPC) **(pmol/mg protein)**	P18:0	514.3843264	507.3683264	10.01849632	14.15772252	4.564827326	2.241320362	4.712264671	14.60155205	26.67748433	28.8308221	33.49820037	12.05026724
Lyso Phosphatidylethanolamine (LPE) **(pmol/mg protein)**	16:0	460.3020879	453.2860879	145.4559753	7.744760445	20.71481225	53.57746527	15.28688021	44.20583076	52.18458151	52.75166036	37.019624	21.84468726
Lyso Phosphatidylethanolamine (LPE) **(pmol/mg protein)**	16:1	458.2864378	451.2704378	52.79736976	21.32071426	26.58004885	45.47213054	11.17233408	17.0530615	49.6394797	30.29780951	10.49817785	20.15729682
Lyso Phosphatidylethanolamine (LPE) **(pmol/mg protein)**	18:0	488.333388	481.317388	30.92799012	14.93153045	43.20019209	156.9640745	33.4204819	35.90524664	23.01844284	26.23790594	52.9867206	33.33466081
Lyso Phosphatidylethanolamine (LPE) **(pmol/mg protein)**	18:1	486.3177379	479.3017379	112.5674579	129.591035	72.09716518	71.2210955	33.34661156	38.24467655	38.53303841	49.80998442	66.51619497	85.2251186
Lyso Phosphatidylethanolamine (LPE) **(pmol/mg protein)**	18:2	484.3020879	477.2860879	47.89140703	124.9759223	153.412153	97.11017009	73.99726564	37.66118561	21.46687844	44.90825152	44.15495727	127.2534367
Lyso Phosphatidylethanolamine (LPE) **(pmol/mg protein)**	20:1	514.3490381	507.3330381	10.1368816	15.74633917	9.907599817	25.2462823	1.520503194	13.50768122	5.320477851	6.335357401	30.16532128	7.914512161
Lyso Phosphatidylethanolamine (LPE) **(pmol/mg protein)**	20:2	512.333388	505.317388	12.97405626	27.87073182	6.478790826	0	2.112152481	7.596256535	8.86886927	12.40613881	47.66717099	8.98311063
Lyso Phosphatidylethanolamine (LPE) **(pmol/mg protein)**	20:4	508.3020879	501.2860879	49.22529333	40.14480756	57.68497188	120.8630318	53.83528551	16.46097973	31.32429218	47.74324748	80.30891005	104.4716617
Lyso Phosphatidylethanolamine (LPE) **(pmol/mg protein)**	P16:0	444.3071732	437.2911732	89.01452607	29.4788269	80.48333801	81.08797288	65.32944406	272.9046802	119.6170743	232.7292535	93.74168928	96.39652862
Lyso Phosphatidylethanolamine (LPE) **(pmol/mg protein)**	P18:0	472.3384734	465.3224734	84.2225998	53.93061771	107.513276	165.8722819	56.00182165	74.59741255	57.2454282	81.05910117	47.42598453	84.24883233
Lyso Phosphatidylethanolamine (LPE) **(pmol/mg protein)**	P18:1	470.3228233	463.3068233	29.08391191	16.61554855	19.06782952	18.81934084	18.3816411	89.69974772	45.61354444	83.7799594	23.47663035	47.60865678
Lyso Phosphatidylethanolamine (LPE) **(pmol/mg protein)**	P20:0	500.3697735	493.3537735	19.38813357	46.81126556	13.13176353	25.94896424	7.008581335	15.62954626	9.406639246	7.721632195	25.69384687	14.80931831
Lyso Phosphatidylethanolamine (LPE) **(pmol/mg protein)**	P22:0	528.4010736	521.3850736	2.731260011	2.694846449	16.4113738	5.970538489	0.344061925	9.434702616	5.747670391	10.24003385	9.696837936	1.183502181
Phosphatidic Acid (PA) **(pmol/mg protein)**	16:0-18:1	673.4808307	674.4886558	6.912482867	2.323217933	4.365685633	1.297434476	5.588048118	8.062856171	8.446086094	9.579734242	7.206153062	2.162847195
Phosphatidic Acid (PA) **(pmol/mg protein)**	16:0-18:2	671.4651807	672.4730057	10.5647149	1.706963444	9.092262089	1.045668229	3.765384781	6.608502511	7.920455168	9.596459606	4.539910355	5.72434733
Phosphatidic Acid (PA) **(pmol/mg protein)**	18:0-18:2/18:1-18:1	699.4964808	700.5043058	15.25789314	6.437155224	8.217266055	6.565806938	4.185730279	17.4161279	17.50583288	18.74275385	23.88992393	14.47716246
Phosphatidic Acid (PA) **(pmol/mg protein)**	18:0-20:4	723.4964808	724.5043058	1.279180536	19.21356709	9.155482512	14.7267918	13.41795811	0.697489037	1.847326822	2.803323832	4.096590794	9.650325847
Phosphatidylcholine (PC)	D14:0-16:0	712.5468549	705.5308549	73.02740876	82.70781332	48.54105005	97.55314902	31.97113723	23.26111346	32.53556566	41.44386016	47.8611005	60.08971171
Phosphatidylcholine (PC)	D16:0-16:0	740.5781551	733.5621551	300.0367869	215.4549675	282.4840322	773.2162197	165.9774538	135.6735744	158.5934002	233.7077663	281.4097743	341.8588059
Phosphatidylcholine (PC)	D16:0-18:0	768.6094552	761.5934552	59.04363212	43.24777977	49.01543527	157.7197278	32.77276114	10.37472317	28.90285593	48.46943825	49.06067312	70.26207909
Phosphatidylcholine (PC)	D16:0-18:1	766.5938051	759.5778051	1992.980821	2209.266346	1641.121367	2584.303052	1016.864657	907.4743719	1006.736669	1347.124405	1708.78681	1967.020641
Phosphatidylcholine (PC)	D16:0-18:2	764.5781551	757.5621551	2339.263692	2277.782181	2410.869258	2375.134874	1120.947425	1295.062372	1069.827568	2081.550267	2283.752054	1448.124624
Phosphatidylcholine (PC)	D16:0-22:6/D18:2-20:4	812.5781551	805.5621551	110.9325756	1.996908797	0.663182421	2.193450031	2.396540386	90.64560828	84.14924666	98.12067762	91.47439275	1.182637543
Phosphatidylcholine (PC)	D16:1-16:0/D14:1-18:0	738.562505	731.546505	431.809417	530.6928319	298.0313176	298.7759406	148.1046413	239.998656	211.2913708	318.244242	306.1782007	274.4789514
Phosphatidylcholine (PC)	D16:1-16:1/D14:1-18:1	736.5468549	729.5308549	43.26955705	55.38447778	31.54254879	22.81629255	12.51262267	28.99420685	21.24594039	36.10057516	25.56748042	19.54231017
Phosphatidylcholine (PC)	D16:1-18:2	762.562505	755.546505	108.2334983	130.8007524	100.491144	87.86982248	46.12601835	74.08081154	41.47839089	110.8421028	93.63319248	54.20335428
Phosphatidylcholine (PC)	D18:0-18:1	794.6251053	787.6091053	637.4333691	579.7626375	245.7127925	696.8500614	247.8427242	206.3859505	246.5727685	297.0952329	425.5683891	455.5946134
Phosphatidylcholine (PC)	D18:0-18:2/D18:1-18:1	792.6094552	785.5934552	1950.902998	1466.857747	991.8620608	1863.173429	783.5761152	594.6090967	654.6898152	1131.379988	1546.949584	1069.116674
Phosphatidylcholine (PC)	D18:0-20:2/P18:2-22:6	820.6407553	813.6247553	20.66455793	18.81142016	12.15310979	29.56982358	19.13830833	8.744261925	28.72394994	19.20045103	50.83970945	14.2244004
Phosphatidylcholine (PC)	D18:0-20:3	818.6251053	811.6091053	73.55403627	66.59778535	29.51443791	81.98030227	27.9501149	68.1557558	64.49831055	70.54135514	73.52934039	58.43915607
Phosphatidylcholine (PC)	D18:1-18:2/D16:0-20:3	790.5938051	783.5778051	662.9253981	651.544032	427.0594725	865.5532615	321.6118686	421.816667	296.2471261	574.7645259	590.705972	448.1676627
Phosphatidylcholine (PC)	D18:1-20:4/D16:0-22:5	814.5938051	807.5778051	163.7722382	6.293416224	1.823981317	8.230031657	5.705269789	133.7219733	124.2866767	165.844655	113.2037309	6.561335361
Phosphatidylcholine (PC)	D18:2-18:2/D16:0-20:4	788.5781551	781.5621551	406.4009746	9.239811789	3.205466301	14.51221076	6.084752742	226.4904707	291.8823508	337.4671053	295.6376368	6.629305411
Phosphatidylcholine (PC)	D18:2-18:3/D16:1-20:4	786.562505	779.546505	34.21739656	7.403100848	4.859824363	13.21010594	7.412089022	25.05005287	25.70094563	24.42189796	13.59433628	4.708721027
Phosphatidylcholine (PC)	D18:2-20:2/D18:0-20:4	816.6094552	809.5934552	351.3006869	8.984393746	5.003541417	16.1755161	8.461329809	212.0888855	233.6349134	268.5421492	195.7032755	13.97307111
Phosphatidylcholine (PC)	0/P18:0-16:0/A18:1-16:0/A	752.6145406	745.5985406	215.5458041	1.626530956	2.278616898	0.251795243	2.607995427	52.24186738	83.99590394	131.4345983	69.90499477	1.379344369
Phosphatidylcholine (PC)	P16:0-18:1/P18:1-16:0	750.5988905	743.5828905	479.103053	6.651337257	4.619713722	4.389397286	4.852424923	343.6965801	244.4813326	490.2497915	249.4991296	4.059801511
Phosphatidylcholine (PC)	P16:0-18:2/A16:0-18:3	748.5832404	741.5672404	580.8951887	5.128124922	3.271091619	1.382420097	4.460347015	409.8819887	390.0543301	762.4049602	325.6450484	2.380358873
Phosphatidylcholine (PC)	P18:0-18:1/P18:1-18:0	778.6301906	771.6141906	211.7012509	4.820578881	3.657256513	4.852089804	6.774929986	74.51296316	86.9674014	164.5039779	79.36170637	4.382983195
Phosphatidylcholine (PC)	P18:0-20:1/P16:0-22:1	806.6614908	799.6454908	183.8684848	15.95995037	12.99003621	8.410416073	14.94072517	14.53717289	64.0485346	133.7575782	167.9351867	7.970362095
Phosphatidylcholine (PC)	P18:1-20:1/P16:1-22:1	804.6458407	797.6298407	158.2951819	3.342278271	4.054582474	3.435463316	5.023032986	20.23666445	54.78440511	68.90262831	40.05742419	5.68504845
Phosphatidylserine (PS) **(pmol/mg protein)**	16:0-16:1	732.4821076	733.4899326	127.0363139	12.030158	14.05221379	5.513069673	5.456601606	25.95139395	26.81449287	28.41455143	16.06809797	4.061536613
Phosphatidylserine (PS) **(pmol/mg protein)**	16:0-18:1	760.5134077	761.5212328	483.4098192	118.780688	180.1935454	80.3476383	75.97086795	147.1043372	215.8376893	225.9096981	224.3292687	69.68213869
Phosphatidylserine (PS) **(pmol/mg protein)**	16:0-18:2	758.4977577	759.5055827	221.8709015	83.70207238	100.9989388	55.0372453	52.23105505	131.1411092	121.8069152	212.5447437	187.1607216	37.89345999
Phosphatidylserine (PS) **(pmol/mg protein)**	18:0-18:1	788.5447079	789.5525329	172.5139659	267.183528	535.2966677	273.2399474	255.4544729	130.6168353	128.2523505	164.8895542	336.9126699	211.5407176
Phosphatidylserine (PS) **(pmol/mg protein)**	18:1-18:2	784.5134077	785.5212328	38.27198031	80.56013638	101.1462172	47.5115029	45.34617014	81.1927704	26.8751324	128.6252619	105.4157493	29.88802001
Sphingomyelin (SM) (nmol/mg protein)	N14:0	681.5522747	674.5362747	0.065232642	0.078914964	0.130385086	0.088225246	0.050582305	0.047995622	0.022361084	0.077655468	0.069305668	0.066903879
Sphingomyelin (SM) (nmol/mg protein)	N15:0	695.5679247	688.5519247	0.058390522	0.057194539	0.079344128	0.065067305	0.039611346	0.010494022	0.026643579	0.062210679	0.050219709	0.048570264
Sphingomyelin (SM) (nmol/mg protein)	N16:0	709.5835748	702.5675748	2.086547775	1.271016947	2.134238644	1.876666735	0.761405048	0.706822161	0.732703394	1.308022186	1.341714189	0.99377342
Sphingomyelin (SM) (nmol/mg protein)	N16:1	707.5679247	700.5519247	0.152068911	0.123025609	0.22104319	0.11864481	0.078219107	0.063311943	0.061660402	0.172092991	0.172621545	0.111276564
Sphingomyelin (SM) (nmol/mg protein)	N18:0	737.6148749	730.5988749	0.52359435	0.185451425	0.194210908	0.310713497	0.075877041	0.072656044	0.107058178	0.207290659	0.194205417	0.133751186
Sphingomyelin (SM) (nmol/mg protein)	N18:1	735.5992249	728.5832249	0.105127617	0.041899312	0.067084471	0.059897515	0.022176581	0.037460893	0.034027823	0.082866501	0.063523797	0.050353565
Sphingomyelin (SM) (nmol/mg protein)	N20:0	765.646175	758.630175	0.1674714	0.163459066	0.197542487	0.189131361	0.085884004	0.032333508	0.053423339	0.198309594	0.140936168	0.080414743
Sphingomyelin (SM) (nmol/mg protein)	N20:1	763.630525	756.614525	0.084911938	0.028558061	0.048587498	0.028908609	0.021014916	0.027640199	0.025006816	0.070741947	0.036177751	0.027991855
Sphingomyelin (SM) (nmol/mg protein)	N22:0	793.6774752	786.6614752	0.362183486	0.324851374	0.467022324	0.371015051	0.203922707	0.073732359	0.11752263	0.318366881	0.274908156	0.17722349
Sphingomyelin (SM) (nmol/mg protein)	N22:1	791.6618251	784.6458251	0.199704455	0.092266234	0.1991877	0.115482417	0.081332132	0.093507716	0.082463695	0.225440705	0.145421639	0.112179043
Sphingomyelin (SM) (nmol/mg protein)	N23:0	807.6931252	800.6771252	0.078979549	0.1142348	0.144311391	0.08569706	0.05664939	0.023693514	0.036013653	0.09053095	0.074778586	0.044821102
Sphingomyelin (SM) (nmol/mg protein)	N23:1	805.6774752	798.6614752	0.057117723	0.03775523	0.082885756	0.036829461	0.027335128	0.023108451	0.016347525	0.063837358	0.045505125	0.043057114
Sphingomyelin (SM) (nmol/mg protein)	N24:0	821.7087753	814.6927753	0.136667417	0.156471496	0.180375755	0.298544053	0.088783804	0.045206556	0.05004336	0.108873938	0.102759926	0.073810336
Sphingomyelin (SM) (nmol/mg protein)	N24:1	819.6931252	812.6771252	0.345545537	0.279219702	0.340141254	0.550840092	0.148926421	0.151757934	0.142463779	0.294395857	0.210710235	0.215804241
Sphingomyelin (SM) (nmol/mg protein)	N24:2	817.6774752	810.6614752	0.122046955	0.07428993	0.097966903	0.12657177	0.037835312	0.064808508	0.063883372	0.118113349	0.087855179	0.086008124
Phosphatidylcholine (PC)	A16:0-16:0	726.5988905	719.5828905	29.78069351					34.91201371	51.82136401	52.73953884	5.484775979	
Phosphatidylcholine (PC)	A18:0-18:1/P18:0-18:0	780.6458407	773.6298407	428.8993118					116.4011779	139.0616957	251.4039862	130.345194	
Phosphatidylcholine (PC)	D18:0-22:5	842.6251053	835.6091053	43.80852838					59.18261041	59.1444154	55.66385068	21.3995914	
Phosphatidylcholine (PC)	D18:0-22:6	840.6094552	833.5934552	70.78569785					82.5181167	61.02389549	72.88365357	42.77499176	
Phosphatidylcholine (PC)	D18:1-22:6/D18:2-22:5	838.5938051	831.5778051	23.38366106					32.42101743	38.98442128	22.05596821	26.84080675	
Phosphatidylinositol (PI) **(pmol/mg protein)**	16:0-18:2	833.5180041	834.5258291	17.83924152					17.35667127	12.46471033	36.68368809	30.2764371	
Phosphatidylinositol (PI) **(pmol/mg protein)**	16:0-20:4	857.5180041	858.5258291	222.3983835					111.6997022	86.29801581	190.3559636	123.9954772	
Phosphatidylinositol (PI) **(pmol/mg protein)**	18:0-18:2	861.5493042	862.5571293	58.52809271					25.06972738	32.23326586	57.2050962	54.70632475	
Phosphatidylinositol (PI) **(pmol/mg protein)**	18:0-20:4	885.5493042	886.5571293	1310.131484					580.407752	579.8805286	1267.767099	693.0650768	
Phosphatidylinositol (PI) **(pmol/mg protein)**	18:2-20:4/16:0-22:6	881.5180041	882.5258291	6.709033042					1.385902498	1.426610374	10.67161851	3.800955984	

Supplementary Table 3. Lipidomics result of human adipose tissue normalized to the protein content.

Diacylglycerol (DAG) (nmol/mg protein)	MASS (m/z)	Molecular Weight	717 VAd	717 SAd	681 VAd	681 SAd	711 VAd	711 SAd	771 VAd	771 SAd	777 VAd	777 SAd	779 VAd	779 SAd	L707 VAd	L707 SAd	L657 VAd	L688 VAd	L752 VAd	L694 SAd	L868 SAd
C32:2	650.536	564.48	5.81	5.90	12.80	15.50	8.60	3.19	9.29	12.64	1.81	1.71	2.69	1.34	5.00	0.94	6.68	1.36	7.80	2.61	8.66
C32:1	652.552	566.49	23.36	53.51	107.27	127.42	82.13	72.99	95.04	116.13	6.17	0.15	58.84	33.63	16.61	10.11	25.07	5.34	25.21	24.45	25.83
C32:0	654.567	568.51	19.92	33.95	25.52	39.78	76.96	66.05	33.62	68.62	9.14	20.90	22.22	27.55	22.03	18.86	13.05	6.54	19.71	22.02	17.12
C34:3	676.552	590.49	12.56	34.91	16.53	17.45	9.05	0.31	19.53	49.75	3.95	0.44	2.85	3.79	9.89	1.70	7.76	2.68	10.09	4.51	7.40
C34:2	678.567	592.51	312.22	530.45	449.01	679.05	766.09	678.43	362.14	511.83	336.42	146.01	491.07	319.97	495.71	237.74	166.84	142.69	98.80	373.17	162.12
C34:1	680.583	594.52	245.02	452.45	291.78	515.57	644.51	773.63	337.30	484.05	49.74	40.21	479.72	320.62	126.34	180.73	88.76	39.43	108.37	186.15	103.88
C34:0	682.599	596.54	6.87	1.34	9.16	11.78	26.10	9.39	11.21	19.55	3.98	2.36	38.15	9.25	7.43	12.37	1.74	2.06	6.96	5.95	5.73
C36:5	700.552	614.49	11.89	7.27	1.68	13.28	5.70	11.57	1.18	10.96	1.81	0.49	1.94	4.76	3.45	0.94	2.77	4.24	4.35	3.85	1.22
C36:4	702.567	616.51	22.68	98.11	20.41	54.60	15.69	28.23	24.82	70.35	7.72	1.03	19.05	21.04	21.84	6.74	9.35	7.37	22.78	12.22	11.30
C36:3	704.583	618.52	72.71	312.47	67.68	107.56	58.06	109.89	99.68	244.90	23.48	3.15	56.64	69.61	60.85	17.27	29.46	20.57	91.82	33.88	36.40
C36:2	706.599	620.54	120.43	289.62	171.20	257.83	372.31	394.81	154.05	216.95	29.28	60.01	304.51	160.03	76.71	99.54	55.60	28.67	76.91	119.02	62.80
C36:1	708.614	622.55	21.87	52.02	28.80	35.67	89.16	37.35	28.51	46.25	8.24	33.03	44.15	23.32	21.75	24.95	12.52	6.91	23.16	26.11	17.91
C38:5	728.583	642.52	11.65	39.78	6.11	24.08	4.00	9.70	10.98	34.03	1.15	0.15	6.53	8.76	3.89	1.93	2.44	0.93	4.33	2.34	1.64
C38:4	730.599	644.54	12.56	52.79	10.28	17.76	5.99	8.91	19.02	65.79	2.62	0.26	5.29	13.54	10.04	4.12	5.69	1.61	5.88	6.89	2.81
C38:3	732.614	646.55	13.16	9.45	10.82	20.02	4.72	10.62	16.60	29.06	1.12	0.17	8.90	13.47	4.93	1.93	2.39	1.03	2.51	2.88	1.68
C38:2	734.630	648.57	9.47	6.79	7.35	0.19	20.42	8.09	3.44	4.00	0.95	35.46	4.89	4.42	2.85	7.19	2.06	1.11	3.42	3.14	2.15
		**SUM**	**922.19**	**1980.82**	**1236.42**	**1937.55**	**2189.48**	**2223.15**	**1226.42**	**1984.84**	**487.57**	**345.53**	**1547.42**	**1035.10**	**889.33**	**627.07**	**432.18**	**272.54**	**512.11**	**829.18**	**468.65**

Ceramide (Cer) (nmol/mg protein)	MASS (m/z)	Molecular Weight	717 VAd	717 SAd	681 VAd	681 SAd	711 VAd	711 SAd	771 VAd	771 SAd	777 VAd	777 SAd	779 VAd	779 SAd	L707 VAd	L707 SAd	L657 VAd	L688 VAd	L752 VAd	L694 SAd	L868 SAd
N16:0	536.504	537.51	2.10	1.84	3.11	1.73	3.85	1.91	3.36	3.11	2.49	2.63	3.27	1.44	3.15	1.60	1.99	0.78	2.95	1.03	1.30
N18:0	564.536	565.54	0.15	0.14	0.18	0.11	0.11	0.11	0.30	0.28	0.39	0.30	0.23	0.07	0.25	0.22	0.13	0.06	0.09	0.09	0.04
N20:0	592.567	593.57	0.16	0.08	0.22	0.10	0.16	0.07	0.18	0.17	0.17	0.11	0.20	0.07	0.19	0.11	0.16	0.05	0.13	0.05	0.04
N22:0	620.598	621.61	0.44	0.39	0.54	0.36	0.99	0.40	0.45	0.55	0.30	0.35	0.56	0.45	0.63	0.48	0.46	0.41	1.05	0.23	0.19
N23:0	634.614	635.62	0.19	0.15	0.19	0.12	0.35	0.10	0.20	0.20	0.14	0.28	0.23	0.19	0.20	0.14	0.20	0.07	0.29	0.05	0.14
N24:2	644.598	645.61	0.10	0.11	0.17	0.10	0.05	0.02	0.23	0.21	0.13	0.08	0.19	0.05	0.21	0.10	0.07	0.01	0.04	0.02	0.03
N24:1	646.614	647.62	0.58	0.49	0.88	0.45	0.53	0.26	1.27	1.09	0.68	0.69	0.97	0.41	0.99	0.50	0.63	0.16	0.38	0.12	0.25
N24:0	648.629	649.64	0.35	0.31	0.49	0.31	0.75	0.29	0.47	0.52	0.36	0.38	0.49	0.37	0.66	0.41	0.36	0.32	0.80	0.14	0.28
N26:1	674.645	675.65	0.05	0.03	0.07	0.05	0.04	0.01	0.06	0.05	0.02	0.04	0.07	0.04	0.00	0.02	0.00	0.00	0.01	0.01	0.01
		**SUM**	**4.12**	**3.55**	**5.84**	**3.32**	**6.83**	**3.18**	**6.42**	**6.18**	**4.69**	**4.86**	**6.21**	**3.10**	**6.29**	**3.58**	**4.00**	**1.88**	**5.74**	**1.75**	**2.28**

Triacylglycerol (TAG) (nmol/mg protein)	MASS (m/z)	Molecular Weight	717 VAd	717 SAd	681 VAd	681 SAd	711 VAd	711 SAd	771 VAd	771 SAd	777 VAd	777 SAd	779 VAd	779 SAd	L707 VAd	L707 SAd	L657 VAd	L688 VAd	L752 VAd	L694 SAd	L868 SAd
C44:2|||753.6584404932	753.658	746.64	4.24	6.57	47.41	44.99	15.83	6.46	28.65	18.52	24.11	3.29	59.66	48.27	48.75	17.50	62.35	39.66	185.76	13.76	88.75
C44:1|||755.6740905574	755.674	748.66	14.63	30.66	168.03	126.81	100.62	54.97	124.62	142.89	77.55	17.66	264.97	199.30	154.77	58.53	226.84	148.72	717.43	45.12	266.20
C44:0:C45:7|||757.6897406216	757.690	750.67	29.75	66.47	101.95	59.17	240.51	95.43	96.03	217.87	69.60	35.84	161.76	234.48	204.99	147.13	228.10	162.07	673.82	70.28	294.81
C45:6|||759.6114903006	759.611	752.60	1.14	0.46	1.01	0.57	1.75	0.11	0.19	0.00	19.32	0.00	0.72	0.00	12.11	0.00	0.22	21.74	1.48	0.73	9.77
C46:4|||777.6584404932	777.658	770.64	1.48	0.47	7.26	6.75	4.14	0.32	0.19	0.34	10.51	0.12	4.37	0.87	4.89	0.14	3.38	27.81	7.31	1.57	10.55
C46:3|||779.6740905574	779.674	772.66	6.24	12.12	148.47	138.67	22.25	16.25	50.10	39.51	23.05	9.37	140.14	75.36	47.52	17.47	80.22	47.80	192.34	17.38	74.22
C46:2|||781.6897406216	781.690	774.67	137.67	216.46	907.44	811.77	530.60	281.52	585.75	688.96	84.91	101.75	939.78	687.71	500.76	426.19	651.54	188.07	1678.18	187.12	611.95
C46:1:C47:8|||783.7053906858	783.705	776.69	381.09	579.74	1456.33	1193.66	1585.18	949.21	1347.11	2482.79	202.30	320.91	1667.42	1483.71	1013.95	961.50	1451.61	432.40	3456.58	476.87	1353.62
C46:0:C47:7|||785.72104075	785.721	778.71	321.65	528.81	507.21	377.97	1291.09	714.39	605.98	1652.85	272.02	451.64	639.25	790.25	841.96	879.73	755.54	542.03	2027.13	491.61	1051.41
C47:6|||787.642790429	787.643	780.63	0.41	0.00	0.96	0.88	1.25	0.04	0.07	0.23	11.85	0.00	0.81	0.00	8.30	0.00	0.30	25.79	1.41	1.66	10.10
C47:0:C48:7|||799.7366908142	799.737	792.72	4.97	23.71	14.77	6.82	57.55	16.50	13.48	25.47	31.90	4.81	28.62	69.34	26.17	8.14	36.26	62.84	155.41	28.37	170.25
C48:6|||801.6584404932	801.658	794.64	0.16	0.21	4.33	3.36	4.64	0.27	1.12	2.73	14.42	0.77	1.65	1.41	12.88	2.34	3.31	20.69	9.32	6.72	16.14
C48:5|||803.6740905574	803.674	796.66	1.19	2.13	20.44	17.21	9.90	2.14	7.57	7.10	16.99	3.09	12.57	9.33	21.16	8.33	14.32	24.00	22.84	7.51	17.17
C48:4|||805.6897406216	805.690	798.67	48.69	59.69	346.07	368.46	90.88	53.02	117.08	109.89	32.12	17.84	282.77	195.87	157.89	82.87	162.24	45.43	366.11	58.44	95.02
C48:3|||807.7053906858	807.705	800.69	409.43	825.18	2673.02	2800.90	1180.34	927.71	1544.62	1878.30	205.33	375.98	2132.05	1617.45	905.41	755.47	1260.74	273.83	2567.88	510.55	1004.01
C48:2:C49:9|||809.72104075	809.721	802.71	2211.64	3935.03	7705.20	7956.34	6064.33	4545.29	6473.64	10531.74	993.87	2346.94	6407.32	5887.58	3777.33	4343.28	4555.44	1325.27	8397.22	2407.71	3912.85
C48:1:C49:8|||811.7366908142	811.737	804.72	3558.57	5538.67	6842.80	6226.16	10093.68	6767.08	7753.08	17943.08	1644.79	3770.76	6953.78	7421.04	5692.67	6849.69	6149.48	2436.27	11906.91	3891.71	5752.81
C48:0:C49:7|||813.7523408784	813.752	806.74	706.19	1099.24	750.60	504.62	2056.54	1167.64	1021.27	2788.64	678.62	1186.92	751.76	934.59	1742.78	2056.28	1055.69	1264.58	3064.86	900.09	1568.41
C49:3:C50:10|||821.72104075	821.721	814.71	8.87	25.57	77.11	123.81	35.01	32.73	59.88	53.59	17.10	14.59	57.12	49.62	16.98	7.89	23.03	25.94	39.33	20.98	42.00
C49:2:C50:9|||823.7366908142	823.737	816.72	112.64	222.31	360.34	473.57	441.65	321.76	426.72	615.14	67.28	106.23	438.07	376.10	135.89	94.79	166.79	106.08	443.38	144.65	318.89
C49:1:C50:8|||825.7523408784	825.752	818.74	248.84	357.86	379.93	444.13	866.32	585.80	578.86	1302.57	134.66	219.61	625.30	627.70	324.68	324.97	356.17	226.28	1027.46	293.95	537.53
C49:0:C50:7|||827.6740905574	827.674	820.66	12.11	53.47	26.98	38.63	73.57	42.37	54.21	105.18	36.84	26.83	49.20	72.12	55.73	24.82	57.24	57.14	139.12	77.07	101.84
C50:6|||829.6897406216	829.690	822.67	3.64	8.10	23.99	38.74	14.79	9.16	9.47	16.51	19.76	7.03	13.99	13.71	39.44	30.47	18.44	33.87	34.28	40.25	18.26
C50:5|||831.7053906858	831.705	824.69	66.06	130.25	409.57	565.77	159.41	135.50	150.20	178.53	56.47	70.66	252.00	231.94	161.61	121.99	200.92	57.00	258.97	127.12	149.22
C50:4|||833.72104075	833.721	826.71	929.66	1945.88	4125.84	4970.85	2021.19	1894.73	2353.92	3098.52	465.38	1043.57	2532.45	2214.51	1459.63	1508.02	1762.14	421.06	2669.87	1058.88	1349.98
C50:3:C51:10|||835.7366908142	835.737	828.72	5235.86	10702.09	18027.92	20643.59	11540.59	10168.27	14591.00	21003.39	2376.33	5843.73	11833.10	10328.81	6927.88	8002.82	8568.63	2308.96	12852.75	5419.98	6618.83
C50:2:C51:9|||837.7523408784	837.752	830.74	12414.52	21837.80	27774.96	30582.24	27765.88	21435.36	30219.70	52939.72	6337.00	14064.91	20982.47	19363.17	17724.82	21878.19	17539.86	7104.21	28831.36	12673.02	15381.22
C50:1:C51:8|||839.7679909426	839.768	832.75	9222.07	13179.94	10103.22	8558.21	20853.58	12896.76	13496.50	34326.63	5712.49	11432.66	9243.93	9534.29	15918.42	21248.01	10295.07	7393.11	24009.02	9141.57	12358.52
C50:0:C51:7|||841.7836410068	841.784	834.77	377.01	508.77	280.62	184.99	859.68	476.91	436.92	1315.03	469.84	686.62	307.50	402.47	995.62	1166.70	436.93	696.49	1665.64	406.51	824.50
C51:5|||845.72104075	845.721	838.71	2.47	4.83	7.97	11.91	5.39	2.45	1.64	5.37	4.17	3.32	5.29	5.70	6.85	3.38	4.11	14.37	5.84	5.58	8.08
C51:4:C52:11|||847.7366908142	847.737	840.72	73.14	102.76	129.69	209.28	116.68	93.67	87.70	128.95	28.34	43.05	86.84	111.65	56.50	52.93	41.38	33.54	70.57	57.75	74.87
C51:2:C52:9|||851.7679909426	851.768	844.75	711.19	1150.88	1250.45	1629.33	1717.85	1258.78	1614.44	2913.03	349.57	680.25	1290.05	1299.23	755.07	873.08	887.87	416.50	1674.66	750.68	976.75
C51:1:C52:8|||853.6897406216	853.690	846.67	228.69	361.41	282.01	407.01	577.36	407.24	477.82	1018.98	157.88	241.28	396.95	358.44	362.93	392.81	306.62	208.34	760.62	347.30	454.14
C51:0:C52:7|||855.7053906858	855.705	848.69	58.21	113.95	58.63	132.74	110.66	94.04	92.83	212.12	65.86	57.29	66.84	89.12	167.67	135.08	97.82	88.04	211.19	300.04	132.35
C52:6|||857.72104075	857.721	850.71	159.60	297.22	504.42	969.87	217.19	272.52	214.09	348.44	100.14	191.22	238.32	298.47	296.86	299.54	278.24	77.39	222.08	547.73	213.68
C52:5|||859.7366908142	859.737	852.72	2333.91	4475.10	5271.68	7265.92	3307.18	3449.42	3063.10	5382.28	1256.76	3236.95	3029.92	3162.64	3465.40	4677.08	2794.13	770.22	3375.58	2704.07	1904.57
C52:4:C53:11|||861.7523408784	861.752	854.74	17046.65	28307.26	26154.50	32079.20	22854.84	19972.96	20541.16	37241.53	8146.53	20173.61	17317.18	17902.34	22255.13	31273.50	14216.04	5096.27	21634.74	15176.84	11609.89
C52:3:C53:10|||863.7679909426	863.768	856.75	43389.76	65106.40	56748.25	67018.27	64038.75	50693.99	59359.09	113481.49	19593.84	45089.10	44141.82	45444.44	53987.95	74978.37	37511.50	15996.82	59391.06	38907.31	29335.07
C52:2:C53:9|||865.7836410068	865.784	858.77	37056.98	51220.61	42338.48	49078.22	62834.72	45162.94	57037.46	121525.65	16990.37	36187.91	38896.68	42265.52	47259.95	64875.28	37760.72	18194.61	61222.56	35546.10	28305.62
C52:1:C53:8|||867.799291071	867.799	860.78	3464.47	4321.16	3211.80	2703.57	7263.24	4342.49	4575.98	12406.30	2649.15	4888.05	3161.15	3638.18	7142.57	9532.14	3748.87	3019.06	11895.51	3383.19	5144.84
C52:0:C53:7|||869.8149411352	869.815	862.80	179.59	78.79	47.64	40.51	152.33	73.59	75.29	205.69	249.68	127.41	57.92	73.01	284.53	217.19	84.38	278.32	434.96	96.32	285.51
C53:6|||871.7366908142	871.737	864.72	0.00	2.25	5.19	11.75	3.35	1.81	0.33	1.19	4.28	3.63	0.58	2.66	4.05	3.99	1.55	9.32	3.10	1.55	9.17
C53:5:C54:12|||873.7523408784	873.752	866.74	35.72	37.43	48.52	89.67	48.06	27.45	16.18	40.65	16.61	20.33	16.19	34.06	30.75	23.92	18.61	26.87	23.75	16.01	43.48
C53:4:C54:11|||875.7679909426	875.768	868.75	253.37	348.24	330.79	499.30	438.00	357.29	335.85	521.59	139.22	233.75	302.21	234.62	266.86	308.04	160.29	100.52	401.65	189.73	239.62
C53:3:C54:10|||877.7836410068	877.784	870.77	815.00	1345.72	1084.01	1457.67	1376.40	1129.29	1274.28	2131.96	452.82	855.22	1073.37	1030.17	1102.61	1324.09	815.91	414.26	1574.21	935.47	806.19
C53:2:C54:9|||879.7053906858	879.705	872.69	716.21	1147.79	720.99	1081.49	1181.69	918.06	1100.24	2181.46	371.59	637.84	934.58	893.94	1025.10	1245.62	749.05	401.71	1468.95	1016.15	746.72
C53:1:C54:8|||881.72104075	881.721	874.71	368.99	588.86	296.05	598.99	571.53	495.11	605.83	1302.84	213.40	295.30	497.06	464.34	641.59	744.13	442.53	270.97	880.90	762.15	435.87
C53:0:C54:7|||883.7366908142	883.737	876.72	359.85	651.00	576.35	1101.93	493.20	473.95	274.00	575.12	261.59	564.61	368.61	492.94	898.55	1099.69	511.05	181.03	654.61	597.61	357.86
C54:6|||885.7523408784	885.752	878.74	2712.29	4891.33	3887.48	5872.63	3869.28	3452.52	2284.53	4707.35	1516.57	3633.14	2554.20	2974.99	4607.83	6575.77	2828.21	1015.52	4710.77	2780.12	2127.29
C54:5:C55:12|||887.7679909426	887.768	880.75	10636.20	18203.74	13923.39	17097.23	15959.80	12823.58	10694.55	18656.64	5473.00	12105.95	9962.92	10218.70	15055.27	21019.37	9947.81	4171.45	19732.61	9428.80	7458.15
C54:4:C55:11|||889.7836410068	889.784	882.77	20640.51	33576.39	27451.29	30542.91	34934.10	26470.14	27568.10	41615.67	10549.12	21524.57	21644.75	20492.26	28248.08	38369.18	21524.46	10257.35	43890.97	19209.38	15920.17
C54:3:C55:10|||891.799291071	891.799	884.78	18682.90	27570.42	24447.33	25358.80	35762.63	25502.35	31089.60	47110.54	9883.03	18735.37	21419.28	20124.21	26868.10	35279.04	24025.34	11620.05	48352.86	18536.78	16122.51
C54:2:C55:9|||893.8149411352	893.815	886.80	5502.91	6631.73	4982.59	3924.46	10508.61	6437.17	6967.03	14761.48	3501.14	6198.90	5062.82	5123.89	9570.58	12610.48	5548.73	3995.50	18292.59	4984.40	5944.92
C54:1:C55:8|||895.8305911994	895.831	888.81	393.03	334.41	274.27	192.33	863.39	430.05	368.96	1111.08	474.87	561.27	296.48	359.37	1036.21	1162.44	362.93	548.43	2372.51	393.61	869.58
C54:0:C55:7|||897.8462412636	897.846	890.83	271.41	30.42	5.97	52.34	71.61	39.11	43.36	51.11	245.57	31.73	22.59	16.56	114.79	32.91	60.15	163.70	145.07	66.83	52.46
C55:6|||899.7679909426	899.768	892.75	7.86	14.29	11.04	30.49	13.85	14.24	10.29	27.63	6.22	15.95	6.57	12.86	16.61	25.39	11.79	11.22	11.51	8.56	15.93
C55:5:C56:12|||901.7836410068	901.784	894.77	58.32	72.02	36.96	62.69	53.02	40.72	33.69	81.97	32.60	42.22	33.49	45.28	78.17	68.78	39.96	36.43	60.39	56.98	68.83
C55:4:C56:11|||903.799291071	903.799	896.78	252.86	311.15	147.41	217.37	326.10	244.52	229.23	356.73	129.54	124.79	239.97	163.12	335.52	333.89	158.58	139.25	511.74	266.20	286.82
C55:3:C56:10|||905.72104075	905.721	898.71	278.88	428.28	205.59	331.01	446.15	352.44	425.29	606.84	150.45	155.32	361.34	255.34	434.41	464.49	290.05	192.50	773.48	395.70	356.92
C55:2:C56:9|||907.7366908142	907.737	900.72	206.94	298.69	136.54	294.40	379.65	309.35	365.19	545.90	129.93	126.43	285.28	203.86	399.01	434.00	255.09	178.95	693.75	357.58	268.48
C55:1:C56:8|||909.7523408784	909.752	902.74	210.59	364.57	181.97	516.11	270.35	295.74	155.17	442.91	120.08	252.87	130.68	241.17	676.28	854.87	169.53	117.21	350.73	216.66	191.42
C55:0:C56:7|||911.7679909426	911.768	904.75	958.74	1557.81	670.93	1840.35	1032.11	1126.36	683.96	2101.73	301.52	1029.73	391.61	898.51	1613.34	2872.98	509.24	253.13	760.70	587.70	439.40
C56:6|||913.7836410068	913.784	906.77	2323.29	3222.91	1336.19	2861.01	2259.06	2076.15	1709.88	5044.33	525.90	1986.00	857.20	1734.60	2114.68	4491.77	1171.36	448.83	1490.74	1039.51	731.84
C56:5:C57:12|||915.799291071	915.799	908.78	2680.08	3097.94	1111.98	1749.32	2217.13	1645.32	1734.71	4822.97	547.52	1771.04	895.20	1479.66	1829.02	3947.78	1249.97	499.61	1689.54	911.32	785.95
C56:4:C57:11|||917.8149411352	917.815	910.80	1959.69	1763.17	794.85	740.71	1690.15	918.18	1336.05	2375.47	460.82	976.88	890.57	870.80	1606.66	2707.61	1073.27	491.23	2023.66	751.67	798.48
C56:3:C57:10|||919.8305911994	919.831	912.81	1280.69	941.84	612.68	469.20	1500.15	689.08	1163.46	1647.68	325.87	542.73	793.98	628.93	1256.08	1668.58	933.34	514.16	2341.24	589.23	667.32
C56:2:C57:9|||921.8462412636	921.846	914.83	286.28	159.69	119.86	81.14	543.30	159.29	215.85	350.50	115.53	128.06	206.12	133.29	413.26	407.75	198.98	222.14	1345.97	135.38	305.87
C56:1:C57:8|||923.8618913278	923.862	916.85	21.91	11.76	12.55	7.36	59.47	18.66	15.51	26.55	37.77	13.59	16.31	19.10	70.53	46.16	21.24	47.65	234.34	16.05	67.44
C56:0:C57:7:C58:14|||925.7836410068	925.784	918.77	7.49	7.86	5.95	7.76	6.10	7.15	7.29	18.37	13.08	7.45	6.32	5.50	23.85	20.49	5.81	13.20	12.51	7.75	16.62
C57:6:C58:13|||927.799291071	927.799	920.78	15.74	20.07	11.17	21.11	15.66	17.04	13.47	31.80	8.61	16.53	7.86	15.46	30.24	35.37	15.07	8.48	17.78	11.62	13.77
C57:5:C58:12|||929.8149411352	929.815	922.80	32.85	38.15	13.27	24.66	25.51	27.47	30.59	63.29	15.38	20.63	22.54	34.65	43.38	45.47	28.68	11.89	33.94	25.46	33.47
C57:4:C58:11|||931.7366908142	931.737	924.72	27.31	29.14	10.49	18.88	20.64	26.34	29.21	56.57	15.27	14.09	26.71	31.29	48.18	45.61	29.48	16.35	37.30	27.40	40.00
C57:3:C58:10|||933.7523408784	933.752	926.74	38.71	36.55	22.26	37.76	29.09	37.34	38.19	79.71	30.43	24.98	37.00	37.13	116.20	106.23	46.05	30.21	68.00	42.76	48.02
C57:2:C58:9|||935.7679909426	935.768	928.75	63.36	78.82	52.76	93.28	67.69	58.73	48.25	122.51	40.37	62.81	45.89	54.20	220.49	227.24	59.04	31.79	113.13	44.37	66.66
C57:1:C58:8|||937.7836410068	937.784	930.77	174.85	176.78	108.43	165.60	150.31	99.72	104.22	232.52	66.16	125.75	70.71	90.98	407.76	492.34	114.82	54.05	161.49	60.75	70.74
C57:0:C58:7:C59:14|||939.799291071	939.799	932.78	427.55	283.32	160.84	204.72	344.87	195.34	239.70	508.84	117.83	201.73	105.98	128.98	477.60	754.13	195.67	81.86	206.20	96.04	131.65
C58:6:C59:13|||941.8149411352	941.815	934.80	438.45	297.52	149.26	171.16	350.71	193.15	273.07	604.24	103.06	178.65	95.72	129.09	322.66	589.12	223.25	77.15	176.44	75.56	113.73
C58:5:C59:12|||943.8305911994	943.831	936.81	224.14	219.94	147.79	173.66	175.43	118.17	136.82	275.19	61.30	119.27	110.14	94.22	144.56	257.67	135.83	57.36	176.99	77.51	109.76
C58:4:C59:11|||945.8462412636	945.846	938.83	249.86	308.44	240.36	310.45	273.22	187.60	196.13	286.00	49.90	123.35	208.67	122.08	106.67	183.04	58.63	73.69	329.35	154.77	123.12
C58:3:C59:10|||947.8618913278	947.862	940.85	194.71	238.19	173.25	221.63	269.61	162.58	176.31	287.46	31.49	89.67	161.68	110.69	95.85	126.69	48.49	68.99	400.90	125.00	110.53
C58:2:C59:9|||949.877541392	949.878	942.86	13.42	12.45	12.83	10.32	55.16	13.73	11.56	17.56	7.49	8.98	18.03	13.84	34.77	30.09	16.26	25.16	243.37	20.11	25.89
C58:1:C59:8:C60:15|||951.8931914562	951.893	944.88	3.07	2.18	1.01	1.09	5.44	2.84	1.94	3.63	5.31	1.07	5.97	3.05	13.04	5.09	3.14	9.13	25.55	7.24	24.36
C58:0:C59:7:C60:14|||953.8149411352	953.815	946.80	4.91	3.99	1.38	2.11	2.28	5.67	4.32	9.26	4.75	1.95	4.07	4.30	13.38	6.69	4.55	6.06	5.26	8.41	13.35
C59:6:C60:13|||955.8305911994	955.831	948.81	13.85	12.14	2.29	3.73	4.67	13.51	14.30	25.90	10.55	6.58	13.60	16.62	21.46	18.12	16.34	7.68	13.29	14.80	16.91
C59:5:C60:12|||957.7523408784	957.752	950.74	19.93	14.88	2.87	5.45	6.14	18.04	19.88	39.59	14.39	7.31	18.64	28.57	35.80	30.32	22.31	8.36	19.73	16.84	34.15
C59:4:C60:11|||959.7679909426	959.768	952.75	26.19	23.34	9.19	14.43	13.32	20.76	21.88	43.87	12.71	11.62	24.86	32.04	40.65	39.00	23.05	12.09	28.42	19.14	35.04
C59:3:C60:10|||961.7836410068	961.784	954.77	26.10	29.92	11.57	21.66	18.29	19.75	18.24	38.60	8.36	13.10	18.17	18.77	32.88	46.81	12.34	7.45	28.58	19.48	25.72
C59:2:C60:9|||963.799291071	963.799	956.78	32.74	30.91	8.65	18.19	17.17	12.96	13.86	38.98	6.04	15.45	11.20	12.32	25.82	47.78	8.81	6.99	19.22	11.84	11.74
C59:1:C60:8:C61:15|||965.8149411352	965.815	958.80	30.33	22.06	5.96	10.78	12.07	10.01	9.59	31.62	6.40	13.53	6.36	7.24	20.96	39.24	10.63	5.29	11.60	5.33	13.11
C59:0:C60:7:C61:14|||967.8305911994	967.831	960.81	41.66	40.29	21.77	30.34	22.16	17.63	17.83	36.03	15.33	19.29	13.65	26.45	37.20	13.23	11.11	33.37	17.41	22.00	
C60:6:C61:13|||969.8462412636	969.846	962.83	86.58	115.42	69.03	92.64	72.96	65.92	63.09	94.60	25.74	37.09	68.82	35.98	49.06	50.12	26.68	32.72	122.20	53.29	43.70
C60:5:C61:12|||971.8618913278	971.862	964.85	140.25	216.35	146.33	188.19	175.45	130.62	116.46	148.29	29.76	63.22	127.77	69.63	55.23	71.13	25.86	55.36	263.52	94.36	120.64
C60:4:C61:11|||973.877541392	973.878	966.86	115.61	173.52	133.07	152.30	171.35	118.96	122.99	141.11	24.38	50.13	117.80	61.21	38.14	59.98	16.73	51.70	273.35	87.74	80.22
C60:3:C61:10|||975.8931914562	975.893	968.88	20.57	27.98	17.89	10.71	28.35	18.44	13.87	28.12	5.29	10.91	18.34	12.03	7.86	15.40	3.33	12.94	72.13	19.69	34.03
C60:0:C61:7:C62:14|||981.7523408784	981.752	974.74	5.04	2.62	0.26	0.00	5.13	3.40	3.40	10.95	6.17	3.50	2.92	10.39	5.87	4.50	4.78	2.58	5.76	13.48	
C61:6:C62:13|||983.7679909426	983.768	976.75	9.32	7.47	0.12	0.04	0.00	12.15	12.94	34.63	12.61	1.82	10.42	5.93	21.90	16.34	16.03	6.60	5.85	14.70	25.69
C61:5:C62:12|||985.7836410068	985.784	978.77	7.50	6.47	1.55	2.22	1.41	9.04	8.28	18.47	5.00	2.21	7.77	3.69	15.59	11.71	8.32	4.48	7.21	8.90	21.73
C61:4:C62:11|||987.799291071	987.799	980.78	7.37	10.40	3.76	7.10	4.39	6.51	5.93	7.32	1.70	2.13	7.03	2.43	5.80	6.22	1.54	3.07	10.60	6.53	8.59
C61:3:C62:10|||989.8149411352	989.815	982.80	5.93	9.23	4.30	6.14	4.81	5.26	4.75	5.14	0.87	1.55	5.10	1.73	1.14	5.14	0.57	1.15	10.63	11.40	4.56
C61:2:C62:9|||991.8305911994	991.831	984.81	4.70	1.63	0.15	1.49	0.24	0.29	0.14	1.27	0.15	0.75	1.04	0.81	2.28	1.20	0.42	0.53	1.70	2.12	9.05
C61:1:C62:8:C63:15|||993.8462412636	993.846	986.83	10.07	9.49	2.18	6.39	1.98	7.44	2.71	10.06	4.52	2.99	4.74	4.91	10.11	5.54	0.37	3.11	5.05	6.32	11.78
C61:0:C62:7:C63:14|||995.8618913278	995.862	988.85	34.00	28.32	5.97	11.33	8.12	24.04	19.22	57.34	15.14	8.43	19.10	17.18	44.10	17.80	2.42	10.41	22.25	16.39	22.48
C62:6:C63:13|||997.877541392	997.878	990.86	48.71	42.24	5.69	7.66	6.40	53.63	57.91	139.05	37.96	13.57	42.53	23.71	85.70	35.58	9.07	28.33	38.93	24.53	28.19
		**SUM**	**215692.49**	**327665.97**	**304275.74**	**347953.12**	**370412.07**	**277592.66**	**321829.59**	**605100.68**	**106493.98**	**226946.22**	**246211.73**	**247917.44**	**297940.38**	**399942.12**	**228140.86**	**106881.13**	**426093.27**	**201836.56**	**190097.38**

Fatty Acyl Chains in TAG (FA) (nmol/mg protein)	MASS (m/z)	Molecular Weight	717 VAd	717 SAd	681 VAd	681 SAd	711 VAd	711 SAd	771 VAd	771 SAd	777 VAd	777 SAd	779 VAd	779 SAd	L707 VAd	L707 SAd	L657 VAd	L688 VAd	L752 VAd	L694 SAd	L868 SAd
228(14:0)	228	228	11267.26	17530.76	22096.00	21465.76	29043.22	18738.90	20464.30	40680.17	4761.55	10658.71	23369.09	24158.37	18903.04	22018.12	18411.35	7814.84	43034.75	11357.05	19535.80
254(16:1)	254	254	19035.38	41293.86	78913.76	93971.59	42328.98	41769.31	71281.69	88746.52	8622.48	20852.87	46570.94	36464.41	20606.30	23498.12	38090.85	8721.44	42900.00	20667.83	25271.34
256(16:0)	256	256	168751.58	246869.78	209573.66	233428.33	286172.69	220693.25	235027.37	517425.79	82957.49	183953.37	174499.14	190632.61	234832.26	319304.11	159925.87	84743.82	286721.43	157581.28	152299.18
278(18:3)	278	278	6512.56	11688.58	11682.65	14301.89	10259.51	8817.43	7347.31	15210.77	4371.52	9496.28	8779.43	8977.87	12652.87	16700.91	10516.70	3462.86	13777.33	8952.58	6464.27
280(18:2)	280	280	123328.02	194331.25	159519.98	184421.84	184304.64	141091.68	136110.47	261845.29	62819.40	138433.11	120012.70	123650.29	173524.81	236474.87	100997.70	47598.86	208278.28	106282.74	92453.97
282(18:1)	282	282	254633.26	387586.29	371390.26	425508.05	464041.10	349262.73	429349.28	737717.73	121318.66	251322.98	314417.75	302114.97	335278.38	448812.49	300297.17	131985.08	543773.99	249597.58	216961.93
284(18:0)	284	284	30851.33	43758.64	35992.73	34175.42	60174.61	39557.85	37835.93	86105.83	23890.82	42349.85	31755.60	33206.06	61591.30	80143.32	33660.78	23348.93	100969.48	31842.49	38893.04
302(20:5)	302	302	898.73	1604.38	1305.98	1977.31	1574.35	1361.17	1409.46	2653.54	757.21	958.56	1241.34	1303.76	1975.54	2337.35	1056.48	847.15	2023.65	1309.12	1261.81
304(20:4)	304	304	3108.67	5941.76	3563.56	8555.38	4959.25	5532.25	4070.45	11662.75	1393.27	3437.35	2692.34	4632.12	4088.20	6793.80	2878.15	1748.20	4873.30	3029.38	2573.49
306(20:3)	306	306	4508.20	6022.01	2382.71	4646.76	3823.03	3039.98	2800.45	8925.91	954.40	2810.85	1638.24	3261.91	2806.10	6150.77	1970.52	1128.21	3273.60	1644.45	1395.13
308(20:2)	308	308	4622.90	4601.53	2307.67	2799.32	3295.88	2262.63	3078.94	6855.32	1262.61	2717.07	2199.06	2393.35	3836.34	7064.84	2289.73	3029.13	3297.60	1991.93	2132.98
310(20:1)	310	310	7557.79	7664.80	5067.73	7087.30	8053.18	5775.97	6931.33	14207.03	2130.80	4822.38	4633.71	5525.32	7428.62	11515.35	5404.28	2586.12	9068.69	3999.31	4101.34
312(20:0)	312	312	3540.30	4262.68	2578.67	3662.60	4386.79	2618.13	2705.80	6580.97	1084.85	2121.24	2042.81	2760.49	3573.07	4941.65	2226.05	1579.43	6656.25	2078.17	2176.52
328(22:6)	328	328	572.99	755.99	553.12	616.96	582.39	713.35	700.83	1725.49	745.21	681.43	654.81	618.63	2931.33	2956.72	707.38	762.95	1022.50	629.49	933.62
330(22:5)	330	330	1179.02	1293.00	1249.49	1686.04	1496.61	1135.07	1284.48	3234.07	913.70	1306.22	840.22	999.26	2838.35	3605.21	1265.84	753.39	1292.25	1088.25	1055.02
332(22:4)	332	332	2221.60	2355.12	1339.33	1720.65	2004.84	1364.60	1829.19	4510.86	840.57	1628.14	945.47	1197.22	1965.46	3315.58	1627.29	739.59	1159.87	725.32	899.15
334(22:3)	334	334	613.53	595.57	491.24	510.87	633.88	566.93	764.89	1748.00	604.90	662.72	502.94	756.65	2066.14	2518.64	1362.14	627.83	855.54	534.40	809.62
336(22:2)	336	336	811.76	917.82	917.70	1143.15	1068.47	747.33	909.86	2063.36	667.29	901.37	626.89	708.18	1806.82	2440.81	927.08	709.47	984.29	641.04	791.65
338(22:1)	338	338	2218.63	2669.95	1808.99	2205.53	2361.69	1562.04	1716.63	3653.55	817.48	1503.73	1349.90	1172.16	1807.89	2649.27	1118.01	878.18	2344.27	1222.11	1313.09
340(22:0)	340	340	2029.54	2931.78	2357.37	2988.69	3117.24	1977.71	2024.96	3051.70	734.92	1059.21	2072.59	1360.86	1211.72	1560.78	849.40	1201.25	5106.85	1532.22	1740.11

			648263.05	984675.53	915092.60	1046873.45	1113682.36	834588.31	967643.63	1818604.65	321649.14	681677.43	740844.96	745894.51	895724.55	1200802.71	685582.77	324266.73	1281413.93	606706.73	573063.06

Phosphatidylcholine (PC) (nmol/mg protein)	MASS (m/z)	Molecular Weight	717 VAd	717 SAd	681 VAd	681 SAd	711 VAd	711 SAd	771 VAd	771 SAd	777 VAd	777 SAd	779 VAd	779 SAd	L707 VAd	L707 SAd	L657 VAd	L688 VAd	L752 VAd	L694 SAd	L868 SAd
D14:0-16:1	710.53	703.52	0.05	0.04	0.17	0.17	0.03	0.05	0.08	0.03	0.01	0.01	0.15	0.12	0.01	0.01	0.05	0.01	0.05	0.04	0.04
D14:0-16:0	712.55	705.53	0.49	0.30	0.43	0.49	0.31	0.44	0.33	0.15	0.14	0.25	0.75	0.70	0.27	0.26	0.29	0.10	0.48	0.41	0.38
P16:0-16:0/A16:0-16:1	724.58	717.57	1.25	1.58	1.18	1.67	0.62	5.35	0.93	1.07	0.64	3.55	1.41	2.61	0.70	1.62	0.61	0.60	0.44	2.17	0.93
A16:0-16:0	726.60	719.58	0.37	0.47	0.32	0.55	0.28	1.23	0.34	0.37	0.23	0.84	0.78	0.82	0.21	0.37	0.20	0.25	0.27	0.84	0.34
D16:1-16:1/D14:1-18:1	736.55	729.53	0.33	0.24	0.57	0.88	0.19	0.39	0.46	0.11	0.09	0.19	0.72	0.53	0.12	0.14	0.31	0.08	0.36	0.31	0.25
D16:0-16:0	740.58	733.56	2.31	1.44	4.97	1.99	1.49	2.20	2.15	0.97	1.51	2.78	4.19	2.73	1.81	1.77	1.17	1.02	1.72	2.00	1.69
P16:0-18:2/A16:0-18:3	748.58	741.57	12.21	13.65	7.41	9.03	5.68	19.19	5.89	4.11	4.35	31.20	12.44	17.02	2.79	16.32	7.91	5.52	5.50	18.01	9.76
P16:0-18:1/P18:1-16:0	750.60	743.58	5.48	8.28	4.79	6.18	3.30	9.44	3.51	2.58	2.51	12.16	7.95	9.65	1.83	6.32	4.31	3.04	4.48	10.70	4.93
P16:0-18:0/P18:0-16:0/A18:1-16:0/A16:0-18	752.61	745.60	1.18	1.88	1.72	1.90	0.68	2.10	1.02	0.80	0.61	1.76	2.32	2.65	0.68	1.08	1.03	0.66	1.30	2.00	0.92
A16:0-18:0	754.63	747.61	0.20	0.18	0.73	0.50	0.36	0.85	0.48	0.25	0.13	0.25	0.78	0.67	0.27	0.06	0.37	0.09	0.33	0.30	0.29
D16:1-18:2	762.56	755.55	0.99	0.82	2.75	1.88	0.59	1.11	1.19	0.35	0.39	0.88	2.16	1.49	0.56	0.69	0.96	0.42	1.18	0.85	0.74
D16:0-18:2	764.58	757.56	17.11	14.53	16.02	17.11	12.05	31.22	22.67	7.67	9.80	23.17	31.08	19.96	12.59	15.16	12.20	10.96	17.74	19.80	10.75
D16:0-18:1	766.59	759.58	8.90	7.73	9.55	10.30	7.13	16.16	13.44	5.15	4.69	12.18	18.14	13.41	6.97	7.21	6.01	4.89	10.33	9.30	5.83
D16:0-18:0	768.61	761.59	0.30	0.16	0.92	0.27	0.22	0.24	0.15	0.07	0.20	0.31	0.45	0.30	0.36	0.33	0.20	0.19	0.36	0.23	0.25
P16:0-20:4/P20:4-16:0/P18:2-18:2	772.58	765.57	0.22	0.51	0.13	0.58	0.10	1.67	0.20	0.37	0.03	1.12	0.16	1.29	0.01	0.49	0.03	0.03	0.02	0.54	0.18
A16:0-20:4	774.60	767.58	0.17	0.33	0.14	0.34	0.08	0.59	0.13	0.16	0.04	0.43	0.17	0.53	0.02	0.20	0.05	0.05	0.07	0.36	0.12
P18:1-18:1	776.61	769.60	4.14	6.46	3.45	5.13	2.07	7.04	2.81	1.84	1.58	9.90	5.46	7.13	1.54	5.34	2.63	2.30	3.31	8.40	4.02
P18:0-18:1/P18:1-18:0	778.63	771.61	1.60	1.83	1.80	3.14	1.07	2.65	1.42	0.98	0.73	2.55	2.81	3.31	0.80	1.89	1.25	1.36	1.94	3.09	1.54
A18:0-18:1/P18:0-18:0	780.65	773.63	3.08	2.47	3.63	3.71	2.85	6.51	2.73	1.78	1.87	4.52	6.44	5.38	2.34	3.26	3.79	2.69	3.88	4.86	2.75
D18:2-18:3/D16:1-20:4	786.56	779.55	0.14	0.16	0.33	0.53	0.13	0.42	0.24	0.14	0.06	0.24	0.46	0.63	0.15	0.25	0.17	0.42	0.24	0.29	0.16
D18:2-18:2/D16:0-20:4	788.58	781.56	2.99	3.20	3.22	4.93	2.59	6.01	4.07	3.15	1.74	3.89	4.97	6.42	2.51	3.24	2.05	3.19	3.64	3.34	2.10
D18:1-18:2/D16:0-20:3	790.59	783.58	4.53	4.31	5.53	6.08	3.36	7.37	5.80	1.73	2.11	5.70	9.92	6.49	2.72	3.55	3.47	3.44	7.27	3.90	3.27
D18:0-18:2/D18:1-18:1	792.61	785.59	8.97	7.74	7.00	8.00	6.52	9.73	7.50	2.64	4.29	10.40	15.88	9.51	7.59	10.54	6.32	5.34	14.80	7.56	4.82
D18:0-18:1	794.63	787.61	2.46	1.86	3.96	2.60	1.89	1.71	1.33	0.83	1.11	1.72	4.39	2.84	2.46	2.82	2.33	1.40	5.49	1.70	1.35
P18:0-20:4	800.61	793.60	0.09	0.29	0.07	0.36	0.05	0.65	0.12	0.17	0.01	0.38	0.10	0.60	0.02	0.18	0.00	0.02	0.03	0.30	0.08
P18:2-20:1/P20:1-18:2/A18:0-20:4	802.63	795.61	0.07	0.09	0.12	0.23	0.07	0.20	0.09	0.09	0.03	0.14	0.14	0.25	0.05	0.11	0.02	0.14	0.08	0.11	0.04
P18:1-20:1/P16:1-22:1	804.65	797.63	0.61	0.76	1.64	2.12	0.82	2.19	1.07	0.89	0.41	1.14	1.40	2.33	0.51	0.89	0.76	1.08	0.93	0.93	0.60
P18:0-20:1/P16:0-22:1	806.66	799.65	0.99	0.75	2.07	2.22	1.05	2.08	1.00	0.76	0.37	1.37	2.31	2.27	0.68	1.05	1.21	1.15	1.84	1.06	1.05
D16:0-22:6/D18:2-20:4	812.58	805.56	0.52	0.53	0.63	0.92	0.40	0.84	0.48	0.45	0.30	0.73	0.83	1.10	0.62	1.02	0.32	0.37	0.85	0.67	0.49
D18:1-20:4/D16:0-22:5	814.59	807.58	0.77	1.00	1.23	2.06	0.88	1.63	1.42	0.81	0.54	1.07	1.78	2.06	0.77	1.03	0.66	0.45	1.31	0.95	0.66
D18:2-20:2/D18:0-20:4	816.61	809.59	1.73	1.98	1.74	2.55	1.56	2.65	1.68	1.41	1.03	2.04	2.45	2.95	1.80	2.37	1.08	0.84	2.25	1.71	0.86
D18:0-20:3	818.63	811.61	0.89	0.54	0.81	0.52	0.32	0.35	0.39	0.26	0.17	0.43	0.68	0.67	0.46	0.65	0.29	0.22	0.59	0.36	0.16
D18:0-20:2/P18:2-22:6	820.64	813.62	0.11	0.11	0.14	0.51	0.09	0.16	0.11	0.10	0.04	0.10	0.20	0.31	0.12	0.17	0.08	0.18	0.19	0.08	0.08
D18:2-22:6	836.58	829.56	0.09	0.10	0.11	0.32	0.07	0.18	0.08	0.12	0.04	0.09	0.14	0.30	0.10	0.20	0.04	0.07	0.18	0.11	0.08
D18:1-22:6/D18:2-22:5	838.59	831.58	0.04	0.20	0.14	0.49	0.12	0.20	0.13	0.17	0.05	0.13	0.15	0.35	0.10	0.19	0.03	0.06	0.14	0.13	0.07
D18:0-22:6	840.61	833.59	0.11	0.23	0.17	0.51	0.15	0.22	0.18	0.14	0.10	0.17	0.26	0.39	0.25	0.37	0.05	0.10	0.20	0.18	0.08
D18:0-22:5	842.63	835.61	0.08	0.28	0.14	0.44	0.24	0.19	0.21	0.13	0.12	0.25	0.21	0.28	0.13	0.22	0.04	0.05	0.18	0.21	0.07
		**SUM**	**85.57**	**87.03**	**89.75**	**101.20**	**59.39**	**145.21**	**85.85**	**42.81**	**42.07**	**138.04**	**144.63**	**130.05**	**54.93**	**91.37**	**62.27**	**52.78**	**93.98**	**107.77**	**61.71**

Lyso Phosphatidylcholine (LPC) (nmol/mg protein)	MASS (m/z)	Molecular Weight	717 VAd	717 SAd	681 VAd	681 SAd	711 VAd	711 SAd	771 VAd	771 SAd	777 VAd	777 SAd	779 VAd	779 SAd	L707 VAd	L707 SAd	L657 VAd	L688 VAd	L752 VAd	L694 SAd	L868 SAd
P16:0	486.35	479.34	0.18	0.46	0.11	0.35	0.13	0.67	0.19	0.23	0.04	1.01	0.19	0.61	0.01	0.52	0.03	0.06	0.07	0.22	0.08
A16:0	488.37	481.35	0.10	0.26	0.07	0.15	0.08	0.20	0.11	0.16	0.04	0.32	0.09	0.25	0.01	0.16	0.01	0.04	0.05	0.15	0.04
16:0	502.35	495.33	0.76	0.82	0.82	0.72	0.58	0.78	0.95	0.74	0.51	0.64	0.96	0.81	0.38	0.98	0.26	0.42	0.72	0.78	0.42
P18:0	514.38	507.37	0.09	0.26	0.07	0.25	0.06	0.29	0.14	0.10	0.02	0.38	0.11	0.32	0.02	0.18	0.01	0.03	0.04	0.15	0.04
18:2	526.35	519.33	1.15	1.21	0.70	0.95	0.65	1.27	1.31	0.85	0.71	1.14	2.00	1.22	1.19	1.13	0.40	0.64	1.12	2.30	1.18
18:1	528.36	521.35	0.50	0.53	0.58	0.58	0.35	0.57	0.80	0.41	0.26	0.38	1.01	0.58	0.36	0.46	0.21	0.26	0.74	0.62	0.44
18:0	530.38	523.36	0.36	0.37	0.48	0.35	0.29	0.25	0.34	0.32	0.27	0.31	0.42	0.29	0.23	0.70	0.13	0.24	0.57	0.26	0.16
		**SUM**	**3.15**	**3.91**	**2.83**	**3.35**	**2.14**	**4.04**	**3.83**	**2.81**	**1.85**	**4.18**	**4.78**	**4.07**	**2.20**	**4.12**	**1.06**	**1.69**	**3.31**	**4.48**	**2.34**

Cardiolipin (CL) (nmol/mg protein)	MASS (m/z)	Molecular Weight	717 VAd	717 SAd	681 VAd	681 SAd	711 VAd	711 SAd	771 VAd	771 SAd	777 VAd	777 SAd	779 VAd	779 SAd	L707 VAd	L707 SAd	L657 VAd	L688 VAd	L752 VAd	L694 SAd	L868 SAd
18:2-18:2-18:2-18:2	723.98	1448.98	2.20	2.58	3.76	2.91	2.71	2.81	3.02	1.68	2.45	3.71	3.37	2.69	2.27	3.49	2.37	2.10	3.85	2.40	1.83
18:2-18:2-18:2-18:1	724.99	1450.99	0.62	1.17	0.88	1.38	0.75	1.51	0.97	0.66	0.32	1.13	0.66	1.35	0.46	1.10	0.60	0.44	0.63	0.78	0.44
18:2-18:2-18:1-18:1	726.00	1453.01	0.05	0.34	0.16	0.42	0.10	0.54	0.00	0.04	0.00	0.06	0.05	0.51	0.00	0.25	0.13	0.10	0.00	0.23	0.08
		**SUM**	**2.87**	**4.09**	**4.80**	**4.71**	**3.56**	**4.87**	**3.98**	**2.38**	**2.78**	**4.90**	**4.08**	**4.54**	**2.73**	**4.84**	**3.10**	**2.64**	**4.48**	**3.40**	**2.35**

Lyso Cardiolipin (LCL) (pmol/mg protein)	MASS (m/z)	Molecular Weight	717 VAd	717 SAd	681 VAd	681 SAd	711 VAd	711 SAd	771 VAd	771 SAd	777 VAd	777 SAd	779 VAd	779 SAd	L707 VAd	L707 SAd	L657 VAd	L688 VAd	L752 VAd	L694 SAd	L868 SAd
18:2-18:2-18:2	592.866	1186.747	158.62	151.29	231.11	199.78	161.43	229.16	328.24	132.33	108.90	341.24	326.11	286.87	162.31	317.58	326.00	305.46	359.97	187.66	386.32
		**SUM**	**158.62**	**151.29**	**231.11**	**199.78**	**161.43**	**229.16**	**328.24**	**132.33**	**108.90**	**341.24**	**326.11**	**286.87**	**162.31**	**317.58**	**326.00**	**305.46**	**359.97**	**187.66**	**386.32**

Phosphatidic Acid (PA) (pmol/mg protein)	MASS (m/z)	Molecular Weight	717 VAd	717 SAd	681 VAd	681 SAd	711 VAd	711 SAd	771 VAd	771 SAd	777 VAd	777 SAd	779 VAd	779 SAd	L707 VAd	L707 SAd	L657 VAd	L688 VAd	L752 VAd	L694 SAd	L868 SAd
16:0-18:2	671.47	672.47	36.43	26.93	46.34	55.48	69.61	190.38	70.02	34.94	19.12	61.76	20.96	95.95	26.67	67.90	29.02	80.09	174.38	71.22	79.55
16:0-18:1	673.48	674.49	21.69	21.80	31.94	42.64	23.71	21.70	42.51	44.62	14.71	39.75	8.13	59.13	10.74	45.18	34.95	33.41	16.07	51.96	46.11
18:0-18:2/18:1-18:1	699.50	700.50	34.07	47.13	61.54	90.12	54.98	29.91	44.86	113.45	44.51	102.82	39.59	82.18	55.62	118.38	151.90	158.00	27.65	128.69	74.73
		**SUM**	**92.19**	**95.86**	**139.82**	**188.25**	**148.30**	**241.99**	**157.39**	**193.00**	**78.35**	**204.34**	**68.69**	**237.26**	**93.03**	**231.45**	**215.87**	**271.49**	**218.11**	**251.87**	**200.39**

'Phosphatidylglycerol (PG) (pmol/mg protein)	MASS (m/z)	Molecular Weight	717 VAd	717 SAd	681 VAd	681 SAd	711 VAd	711 SAd	771 VAd	771 SAd	777 VAd	777 SAd	779 VAd	779 SAd	L707 VAd	L707 SAd	L657 VAd	L688 VAd	L752 VAd	L694 SAd	L868 SAd
16:0-18:1	747.52	748.53	141.92	262.62	203.20	277.85	121.84	317.08	162.76	91.07	107.45	252.18	349.48	292.85	94.97	317.23	86.37	57.43	127.64	178.39	127.15
18:2-18:2	769.50	770.51	160.21	317.34	139.79	340.19	100.73	202.42	166.81	52.03	86.47	302.94	208.76	248.22	62.91	204.33	66.91	69.26	147.26	94.83	37.64
18:1-18:2	771.52	772.53	339.55	684.67	323.04	738.24	245.82	449.98	401.53	107.81	182.31	642.63	491.83	552.79	110.42	405.50	128.42	74.12	270.05	162.19	83.43
18:1-18:1	773.53	774.54	299.92	579.91	340.00	748.33	262.95	569.65	448.56	163.88	188.59	653.36	626.82	703.50	107.61	414.24	156.12	95.70	272.19	220.00	107.41
		**SUM**	**941.60**	**1844.54**	**1006.02**	**2104.61**	**731.35**	**1539.12**	**1179.66**	**414.78**	**564.83**	**1851.11**	**1676.90**	**1797.37**	**375.91**	**1341.31**	**437.81**	**296.51**	**817.14**	**655.41**	**355.63**

Phosphatidylserine (PS) (nmol/mg protein)	MASS (m/z)	Molecular Weight	717 VAd	717 SAd	681 VAd	681 SAd	711 VAd	711 SAd	771 VAd	771 SAd	777 VAd	777 SAd	779 VAd	779 SAd	L707 VAd	L707 SAd	L657 VAd	L688 VAd	L752 VAd	L694 SAd	L868 SAd
16:0-16:1	732.48	733.49	0.09	0.17	0.36	0.74	0.14	0.60	0.42	0.20	0.11	0.11	0.39	0.53	0.09	0.16	0.07	0.04	0.10	0.09	0.14
16:0-18:2	758.50	759.51	1.14	1.09	1.73	2.25	1.19	3.75	1.78	0.70	0.60	4.07	2.22	2.25	0.73	1.36	0.59	0.67	1.25	0.92	1.07
16:0-18:1	760.51	761.52	1.06	1.34	2.27	3.64	1.15	3.90	1.84	1.05	0.47	2.40	2.73	3.40	0.55	1.01	0.66	0.74	1.25	1.04	1.06
18:2-18:2	782.50	783.51	0.18	0.21	0.23	0.67	0.19	0.92	0.35	0.24	0.03	0.83	0.23	0.69	0.06	0.23	0.08	0.09	0.14	0.10	0.18
18:1-18:2	784.51	785.52	0.90	0.98	1.45	2.35	0.77	2.41	1.35	0.49	0.30	2.99	2.04	2.02	0.39	1.01	0.50	0.39	1.24	0.59	0.69
18:0-18:2	786.53	787.54	9.83	10.35	8.26	13.02	6.85	17.61	6.87	3.45	4.29	22.16	14.85	12.02	5.10	13.38	3.67	5.42	8.85	8.02	4.93
18:0-18:1	788.54	789.55	3.89	3.09	4.37	5.96	2.77	4.60	2.66	1.47	1.79	4.25	7.62	5.55	2.61	3.77	1.43	3.07	4.34	2.25	1.72
18:1-20:4	808.51	809.52	0.20	0.32	0.33	0.78	0.33	0.98	0.28	0.22	0.09	0.83	0.30	0.65	0.12	0.73	0.17	0.14	0.20	0.18	0.17
18:0-20:4	810.53	811.54	1.75	2.32	1.19	3.38	1.29	4.14	1.71	1.25	0.52	5.10	2.02	3.49	0.84	2.18	0.43	0.68	1.29	1.09	0.78
18:0-22:6	834.53	835.54	0.19	0.39	0.27	0.53	0.16	0.37	0.21	0.09	0.09	0.50	0.27	0.59	0.72	1.29	0.06	0.00	0.32	0.34	0.13
20:0-20:4/18:0-22:4	838.56	839.57	0.30	0.48	0.25	0.34	0.22	0.71	0.25	0.22	0.19	0.64	0.37	0.46	0.26	0.26	0.07	0.09	0.10	0.14	0.02
		**SUM**	**19.54**	**20.74**	**20.71**	**33.66**	**15.06**	**40.01**	**17.73**	**9.38**	**8.46**	**43.89**	**33.02**	**31.64**	**11.48**	**25.38**	**7.73**	**11.33**	**19.08**	**14.77**	**10.89**

Phosphatidylinositol (PI) (nmol/mg protein)	MASS (m/z)	Molecular Weight	717 VAd	717 SAd	681 VAd	681 SAd	711 VAd	711 SAd	771 VAd	771 SAd	777 VAd	777 SAd	779 VAd	779 SAd	L707 VAd	L707 SAd	L657 VAd	L688 VAd	L752 VAd	L694 SAd	L868 SAd
16:0-18:2	833.518	834.526	0.06	0.04	0.08	0.11	0.03	0.09	0.01	0.03	0.05	0.10	0.12	0.16	0.04	0.08	0.12	1.29	0.09	0.04	0.14
16:0-20:4	857.518	858.526	0.31	0.22	0.68	0.58	0.43	0.63	0.26	0.25	0.22	0.65	0.58	0.70	0.28	0.39	0.74	0.41	0.30	0.36	0.35
18:0-18:2	861.549	862.557	0.09	0.06	0.13	0.16	0.05	0.18	0.06	0.07	0.11	0.25	0.21	0.28	0.07	0.12	0.25	0.35	0.25	0.05	0.34
18:2-20:4/16:0-22:6	881.518	882.526	0.02	0.01	0.06	0.06	0.01	0.03	0.02	0.00	0.00	0.03	0.11	0.09	0.01	0.02	0.07	0.02	0.03	0.03	0.04
18:1-20:4	883.534	884.541	0.49	0.39	1.71	1.27	0.72	1.12	0.71	0.47	0.42	1.46	1.60	1.40	0.40	0.60	1.50	0.41	0.70	0.55	0.57
18:0-20:4	885.549	886.557	2.69	2.54	3.28	2.40	2.05	3.71	1.68	2.27	2.51	4.25	2.43	4.04	2.51	3.69	4.30	2.22	1.87	3.39	1.68
		**SUM**	**3.67**	**3.27**	**5.94**	**4.57**	**3.29**	**5.76**	**2.76**	**3.09**	**3.32**	**6.75**	**5.05**	**6.67**	**3.31**	**4.89**	**6.98**	**4.71**	**3.23**	**4.41**	**3.13**

Sphingomyelin (SM) (nmol/mg protein)	MASS (m/z)	Molecular Weight	717 VAd	717 SAd	681 VAd	681 SAd	711 VAd	711 SAd	771 VAd	771 SAd	777 VAd	777 SAd	779 VAd	779 SAd	L707 VAd	L707 SAd	L657 VAd	L688 VAd	L752 VAd	L694 SAd	L868 SAd
N14:0	681.55	674.54	0.37	0.49	0.29	0.61	0.37	0.69	0.27	0.20	0.16	0.26	0.62	0.69	0.19	0.37	0.17	0.19	0.40	0.38	0.26
N15:0	695.57	688.55	0.22	0.30	0.18	0.36	0.27	0.45	0.18	0.15	0.13	0.18	0.44	0.45	0.16	0.24	0.10	0.13	0.26	0.22	0.22
N16:1	707.57	700.55	0.77	1.42	0.79	1.83	0.77	2.11	0.71	0.70	0.49	1.18	0.89	1.61	0.40	1.24	0.33	0.48	0.67	1.21	0.55
N16:0	709.58	702.57	4.53	5.25	3.72	5.34	3.84	4.80	4.45	3.13	3.87	4.91	6.82	5.38	3.88	5.78	2.80	3.15	5.23	5.75	4.62
N18:1	735.60	728.58	0.27	0.32	0.23	0.37	0.18	0.32	0.30	0.24	0.28	0.48	0.37	0.30	0.15	0.46	0.09	0.16	0.15	0.38	0.12
N18:0	737.61	730.60	0.61	0.67	0.44	0.59	0.35	0.51	0.70	0.52	0.60	0.99	0.84	0.49	0.50	0.93	0.28	0.42	0.45	0.74	0.35
N20:1	763.63	756.61	0.33	0.22	0.27	0.46	0.22	0.23	0.32	0.16	0.17	0.32	0.33	0.26	0.16	0.46	0.13	0.17	0.22	0.42	0.11
N20:0	765.65	758.63	0.66	0.54	0.51	0.68	0.56	0.51	0.62	0.30	0.34	0.64	0.94	0.67	0.56	0.82	0.37	0.54	0.84	0.74	0.42
N22:1	791.66	784.65	0.93	0.98	0.74	1.29	1.01	1.62	0.83	0.48	0.48	0.98	1.13	1.47	0.52	1.52	0.44	0.92	1.42	1.33	0.42
N22:0	793.68	786.66	0.94	1.56	0.91	1.54	0.99	1.85	0.76	0.71	0.71	1.63	1.24	2.13	1.09	2.06	0.82	1.42	2.42	1.63	1.25
N23:1	805.68	798.66	0.29	0.36	0.23	0.45	0.30	0.45	0.31	0.19	0.15	0.30	0.43	0.56	0.12	0.42	0.18	0.15	0.39	0.27	0.22
N23:0	807.69	800.68	0.29	0.51	0.22	0.46	0.32	0.51	0.26	0.22	0.17	0.44	0.39	0.73	0.28	0.50	0.28	0.20	0.61	0.34	0.50
N24:2	817.68	810.66	0.54	0.73	0.53	1.03	0.28	0.55	0.95	0.51	0.43	0.84	0.86	0.73	0.42	1.08	0.33	0.27	0.36	0.50	0.29
N24:1	819.69	812.68	0.99	1.53	0.99	1.86	0.77	1.54	1.57	0.99	0.91	1.69	1.64	1.75	0.99	2.13	0.88	0.86	1.32	1.22	1.02
N24:0	821.71	814.69	0.27	0.59	0.33	0.62	0.31	0.69	0.28	0.27	0.31	0.66	0.46	0.74	0.49	0.80	0.31	0.52	0.75	0.50	0.63
		**SUM**	**12.00**	**15.48**	**10.37**	**17.48**	**10.53**	**16.83**	**12.52**	**8.79**	**9.20**	**15.50**	**17.39**	**17.98**	**9.89**	**18.80**	**7.51**	**9.60**	**15.48**	**15.64**	**10.98**

Phosphatidylethanolamine (PE) (nmol/mg protein)	MASS (m/z)	Molecular Weight	717 VAd	717 SAd	681 VAd	681 SAd	711 VAd	711 SAd	771 VAd	771 SAd	777 VAd	777 SAd	779 VAd	779 SAd	L707 VAd	L707 SAd	L657 VAd	L688 VAd	L752 VAd	L694 SAd	L868 SAd
P16:0-18:2	920.51	699.52	11.56	7.80	12.85	8.39	7.14	6.48	6.95	3.97	6.71	13.94	14.71	6.49	7.20	9.66	9.94	5.85	11.52	7.10	4.46
P18:1-16:0/P16:0-18:1	922.53	701.54	4.95	4.99	6.39	6.63	3.30	6.34	4.13	4.43	3.93	5.48	6.62	5.37	2.18	3.69	2.87	1.92	2.81	3.13	1.69
P18:0-16:0/P16:0-18:0	924.54	703.55	0.33	0.36	0.47	0.43	0.30	0.53	0.33	0.42	0.33	0.38	0.39	0.57	0.44	0.38	0.24	0.51	0.67	0.33	0.28
D16:1-18:2	934.49	713.50	0.97	0.55	1.03	0.76	0.76	0.61	0.61	0.37	0.62	1.13	1.37	0.70	0.66	0.66	0.89	0.83	1.78	0.57	0.48
D16:0-18:2/D16:1-18:1	936.51	715.52	1.79	1.43	2.79	2.30	1.68	2.50	1.80	1.31	1.77	2.04	2.19	2.30	1.41	1.56	1.39	2.92	3.34	1.43	1.58
D16:0-18:1	938.52	717.53	1.42	1.21	2.36	2.52	1.35	1.95	1.85	1.75	1.69	1.23	1.90	2.72	1.09	1.24	1.02	1.30	1.93	1.08	1.16
P16:1-20:4/ P14:0-22:5	942.50	721.51	0.28	0.38	0.80	1.39	0.42	1.34	0.49	0.67	0.53	0.92	0.95	1.44	0.52	1.09	0.35	0.20	0.56	1.01	0.34
P16:0-20:4	944.51	723.52	12.38	17.05	14.00	26.70	11.66	30.94	15.31	17.02	14.80	23.19	13.82	25.94	7.59	15.51	5.40	3.12	9.84	13.83	6.57
P16:0-20:3/P18:1-18:2	946.53	725.54	8.57	5.99	7.68	7.16	4.43	4.83	5.51	3.91	5.45	8.01	10.28	6.47	3.95	6.33	5.93	2.57	8.65	4.27	2.75
P18:1-18:1/P18:0-18:2/P16:0-20:2	948.54	727.55	11.15	7.13	9.27	6.71	8.13	6.74	6.24	4.42	6.15	11.90	13.37	7.21	7.25	9.97	7.18	5.57	19.44	6.42	5.09
P18:0-18:1/P16:0-20:1	950.56	729.57	3.16	2.82	2.76	2.06	2.38	3.11	1.98	1.91	1.96	3.23	3.59	2.92	1.95	3.26	1.53	1.42	3.52	2.09	1.21
A18:0-18:1	952.58	731.58	0.16	0.10	0.27	0.23	0.20	0.16	0.17	0.37	0.16	0.21	0.24	0.29	0.41	0.29	0.15	0.27	0.58	0.20	0.20
D16:1-20:4	958.49	737.50	0.93	1.04	1.13	1.86	1.06	2.08	1.28	1.37	1.18	1.83	1.19	2.37	0.59	1.41	0.44	0.85	0.91	0.92	0.61
D16:0-20:4/D18:2-18:2	960.51	739.52	0.97	1.15	1.29	2.06	0.83	1.82	1.32	1.23	1.35	1.41	0.96	2.21	0.88	1.15	0.79	3.07	1.81	0.87	0.83
D18:1-18:2/D16:0-20:3	962.52	741.53	1.38	1.04	1.96	1.72	1.05	1.38	1.45	0.99	1.44	1.37	1.62	1.57	0.94	1.20	1.10	1.84	3.43	0.88	0.98
D18:0-18:2/D18:1-18:1/D16:0-20:2	964.54	743.55	4.75	4.50	4.60	5.01	3.43	4.83	3.93	3.67	3.67	4.21	4.62	5.22	2.88	4.96	2.63	3.43	8.89	4.07	2.70
D18:0-18:1/D16:0-20:1	966.55	745.56	2.15	1.99	2.10	2.97	1.59	1.84	1.78	1.99	1.62	1.40	2.35	3.04	1.60	2.09	1.08	1.46	2.81	1.47	0.97
P16:0-22:6/D18:0-18:0/P18:2-20:4	968.51	747.52	0.76	0.92	1.28	1.96	0.75	1.75	0.96	0.89	1.04	1.28	1.58	2.44	1.39	2.09	0.45	0.31	0.74	1.26	0.62
P18:1-20:4/P16:0-22:5	970.53	749.54	7.72	8.92	8.89	17.70	6.76	15.14	11.52	8.99	11.26	11.07	10.25	17.67	4.33	8.66	3.40	1.89	6.07	7.20	3.72
P18:0-20:4/P16:0-22:4/P18:1-20:3	972.54	751.55	11.15	12.68	8.62	10.97	12.51	20.96	11.61	12.55	11.19	17.55	10.45	18.00	7.88	15.12	3.95	2.34	12.63	10.42	6.98
P18:0-20:3	974.56	753.57	2.19	1.93	1.21	1.10	1.22	1.21	1.20	1.54	1.16	2.13	1.31	1.71	1.27	2.11	0.95	0.21	2.27	1.01	0.62
P20:1-18:1/P18:1-20:1	976.58	755.58	0.58	0.47	0.36	0.35	0.27	0.39	0.33	0.33	0.29	0.43	0.45	0.49	0.34	0.39	0.22	0.18	0.46	0.20	0.12
D18:1-20:4D/16:0-22:5	986.52	765.53	0.69	0.78	0.95	1.73	0.60	1.30	1.14	0.98	1.11	1.03	0.78	1.57	0.55	0.84	0.60	0.33	1.14	0.68	0.50
D18:0-20:4/D16:0-22:4	988.54	767.55	3.53	4.52	2.90	5.76	2.35	5.01	3.70	4.38	3.50	4.14	2.61	5.82	2.30	3.94	1.96	1.15	3.90	3.02	1.62
D18:0-20:3/D18:1-20:2/D16:0-22:3	990.55	769.56	0.66	0.62	0.34	0.60	0.26	0.49	0.38	0.64	0.38	0.35	0.31	0.67	0.33	0.53	0.19	0.11	0.33	0.30	0.11
		**SUM**	**94.17**	**90.38**	**96.29**	**119.04**	**74.41**	**123.74**	**85.98**	**80.11**	**83.30**	**119.85**	**107.90**	**125.21**	**59.92**	**98.14**	**54.64**	**43.65**	**110.03**	**73.74**	**46.19**

Lyso Phosphatidylethanolamine (LPE) (nmol/mg protein)	MASS (m/z)	Molecular Weight	717 VAd	717 SAd	681 VAd	681 SAd	711 VAd	711 SAd	771 VAd	771 SAd	777 VAd	777 SAd	779 VAd	779 SAd	L707 VAd	L707 SAd	L657 VAd	L688 VAd	L752 VAd	L694 SAd	L868 SAd
P16:0	444.31	437.29	0.78	0.45	0.88	0.63	0.47	0.61	0.37	0.35	0.07	0.67	0.28	0.54	0.03	0.92	0.61	0.68	0.26	0.27	0.25
16:1	458.29	451.27	0.10	0.11	0.26	0.27	0.21	0.17	0.22	0.26	0.16	0.10	0.21	0.19	0.11	0.17	0.49	0.44	0.13	0.12	0.21
16:0	460.30	453.29	0.11	0.09	0.16	0.18	0.12	0.14	0.11	0.14	0.05	0.15	0.09	0.15	0.05	0.16	0.20	0.36	0.09	0.07	0.10
P18:1	470.32	463.31	0.46	0.21	0.55	0.54	0.25	0.35	0.32	0.16	0.03	0.32	0.24	0.41	0.01	0.44	0.31	0.25	0.17	0.10	0.13
P18:0	472.34	465.32	0.49	0.21	0.37	0.26	0.36	0.27	0.23	0.22	0.07	0.46	0.16	0.27	0.03	0.61	0.38	0.48	0.32	0.16	0.26
18:2	484.30	477.29	0.30	1.08	1.10	1.04	1.95	0.85	1.33	0.94	1.50	1.77	2.59	0.88	2.43	1.98	1.20	3.10	2.00	1.45	2.04
18:1	486.32	479.30	0.31	0.63	0.71	0.87	0.82	0.70	0.77	0.96	0.42	0.64	0.95	0.77	0.73	0.78	0.52	1.17	0.64	0.60	0.74
18:0	488.33	481.32	0.25	0.15	0.20	0.21	0.17	0.14	0.16	0.15	0.07	0.16	0.08	0.19	0.06	0.31	0.29	0.32	0.18	0.11	0.13
P20:0	500.37	493.35	0.07	0.07	0.10	0.10	0.22	0.11	0.10	0.11	0.16	0.18	0.19	0.09	0.23	0.17	0.20	0.50	0.18	0.13	0.25
20:4	508.30	501.29	0.34	2.07	1.08	2.79	3.01	3.24	2.99	3.96	0.56	2.77	2.37	3.37	2.51	3.08	0.72	1.28	1.35	2.73	2.99
20:2	512.33	505.32	0.09	0.09	0.11	0.07	0.05	0.02	0.03	0.11	0.04	0.05	0.05	0.03	0.17	0.12	0.03	0.02	0.06	0.04	0.04
		**SUM**	**3.31**	**5.16**	**5.51**	**6.96**	**7.64**	**6.62**	**6.65**	**7.36**	**3.14**	**7.27**	**7.20**	**6.87**	**6.36**	**8.74**	**4.95**	**8.60**	**5.38**	**5.78**	**7.14**

Acylcarnitine (AC) (pmol/mg protein)	MASS (m/z)	Molecular Weight	717 VAd	717 SAd	681 VAd	681 SAd	711 VAd	711 SAd	771 VAd	771 SAd	777 VAd	777 SAd	779 VAd	779 SAd	L707 VAd	L707 SAd	L657 VAd	L688 VAd	L752 VAd	L694 SAd	L868 SAd
7:0	274.20	273.19	37.88	20.73	9.42	11.78	15.02	46.13	10.95	28.17	4.15	6.72	2.68	12.13	27.18	6.61	18.02	6.04	16.86	14.81	17.84
9:0	302.23	301.22	11.40	8.11	3.58	3.92	5.28	13.41	3.47	7.88	1.63	2.22	0.79	3.60	10.13	2.46	3.47	1.56	6.02	6.27	7.41
20:4	448.34	447.33	0.37	2.34	1.52	0.00	0.92	1.69	0.21	2.68	0.90	0.13	0.31	0.73	1.10	0.41	2.71	1.65	0.07	2.18	2.66
		**SUM**	**49.64**	**31.18**	**14.52**	**15.70**	**21.22**	**61.22**	**14.63**	**38.73**	**6.69**	**9.07**	**3.78**	**16.46**	**38.41**	**9.48**	**24.20**	**9.25**	**22.95**	**23.26**	**27.91**

Supplementary Table 4. Lipid–lipid Pearson correlation matrix ofEV lipidomes from lean and obese individuals (related to Fig. 1F)

	SM(N18:0)	SM(N24:0)	SM(N24:1)	LPE(P18:0)	SM(N24:2)	LPE(20:4)	PC(D16:0-16:0)	PC(D16:0-18:0)	PS(18:0-18:1)	SM(N16:1)	SM(N23:0)	LPE(18:2)	SM(N15:0)	SM(N16:0)	SM(N20:0)	SM(N22:0)	PC(D16:0-18:2)	[illegible]
SM(N18:0)	1	0.80394	0.82316	0.60467	0.73755	0.49397	0.72803	0.74338	0.35362	0.44793	0.53788	0.4568	0.61438	0.74644	0.71732	0.6673	0.66262	[illegible]
SM(N24:0)	0.80394	1	0.97733	0.83833	0.83716	0.63669	0.84923	0.83731	0.65592	0.62331	0.75105	0.65485	0.80713	0.87594	0.89145	0.8558	0.86525	[illegible]
SM(N24:1)	0.82316	0.97733	1	0.90154	0.92585	0.70427	0.91057	0.89536	0.60455	0.61045	0.68647	0.67982	0.78718	0.87324	0.84195	0.80501	0.84408	[illegible]
LPE(P18:0)	0.60467	0.83833	0.90154	1	0.89143	0.78666	0.90887	0.87203	0.59019	0.53027	0.49866	0.65266	0.68207	0.7735	0.63013	0.64857	0.67496	[illegible]
SM(N24:2)	0.73755	0.83716	0.92585	0.89143	1	0.77106	0.88273	0.86231	0.57952	0.66312	0.61379	0.75844	0.76691	0.84111	0.74019	0.71808	0.80465	[illegible]
LPE(20:4)	0.49397	0.63669	0.70427	0.78666	0.77106	1	0.85443	0.86608	0.50572	0.47411	0.35337	0.67844	0.67987	0.5727	0.50765	0.51413	0.55807	[illegible]
PC(D16:0-16:0)	0.72803	0.84923	0.91057	0.90887	0.88273	0.85443	1	0.99297	0.39737	0.38494	0.37855	0.5229	0.60997	0.67203	0.60173	0.558	0.62243	[illegible]
PC(D16:0-18:0)	0.74338	0.83731	0.89536	0.87203	0.86231	0.86608	0.99297	1	0.36648	0.36391	0.36934	0.51437	0.61681	0.6463	0.60507	0.55146	0.60139	[illegible]
PS(18:0-18:1)	0.35362	0.65592	0.60455	0.59019	0.57952	0.50572	0.39737	0.36648	1	0.93239	0.89	0.84929	0.89659	0.85743	0.80714	0.8992	0.82148	[illegible]
SM(N16:1)	0.44793	0.62331	0.61045	0.53027	0.66312	0.47411	0.38494	0.36391	0.93239	1	0.90281	0.85527	0.90624	0.88662	0.84431	0.90273	0.83775	[illegible]
SM(N23:0)	0.53788	0.75105	0.68647	0.49866	0.61379	0.35337	0.37855	0.36934	0.89	0.90281	1	0.82815	0.91503	0.88408	0.94348	0.96595	0.91981	[illegible]
LPE(18:2)	0.4568	0.65485	0.67982	0.65266	0.75844	0.67844	0.5229	0.51437	0.84929	0.85527	0.82815	1	0.91941	0.81009	0.77758	0.82846	0.87243	[illegible]
SM(N15:0)	0.61438	0.80713	0.78718	0.68207	0.76691	0.67987	0.60997	0.61681	0.89659	0.90624	0.91503	0.91941	1	0.91299	0.93309	0.9602	0.90249	[illegible]
SM(N16:0)	0.74644	0.87594	0.87324	0.7735	0.84111	0.5727	0.67203	0.6463	0.85743	0.88662	0.88408	0.81009	0.91299	1	0.91933	0.9528	0.90733	[illegible]
SM(N20:0)	0.71732	0.89145	0.84195	0.63013	0.74019	0.50765	0.60173	0.60507	0.80714	0.84431	0.94348	0.77758	0.93309	0.91933	1	0.97828	0.92995	[illegible]
SM(N22:0)	0.6673	0.8558	0.80501	0.64857	0.71808	0.51413	0.558	0.55146	0.8992	0.90273	0.96595	0.82846	0.9602	0.9528	0.97828	1	0.92474	[illegible]
PC(D16:0-18:2)	0.66262	0.86525	0.84408	0.67496	0.80465	0.55807	0.62243	0.60139	0.82148	0.83775	0.91981	0.87243	0.90249	0.90733	0.92995	0.92474	1	[illegible]
SM(N14:0)	0.51971	0.76045	0.76114	0.71493	0.78495	0.57248	0.54502	0.51213	0.93533	0.95203	0.91221	0.92034	0.93297	0.93598	0.87967	0.93023	0.92033	[illegible]
PA(18:0-20:4)	0.44794	0.71626	0.65808	0.53965	0.5477	0.61758	0.5231	0.54177	0.67884	0.56371	0.74194	0.80855	0.80507	0.61771	0.75196	0.74726	0.80483	[illegible]
PC(D18:0-18:1)	0.71785	0.75388	0.77969	0.61832	0.74661	0.72144	0.7659	0.78733	0.36517	0.36588	0.50599	0.66491	0.64325	0.5731	0.63787	0.57094	0.74625	[illegible]
PC(D18:0-18:2/D18:	0.78559	0.86218	0.8531	0.66589	0.77172	0.7328	0.78978	0.80459	0.53793	0.52765	0.65497	0.71421	0.76706	0.71836	0.78817	0.73499	0.84856	[illegible]
PC(D18:1-18:2/D16:	0.70361	0.87558	0.88411	0.73984	0.80846	0.67943	0.81314	0.80485	0.48748	0.46285	0.61223	0.68247	0.68816	0.68501	0.74081	0.67506	0.8477	[illegible]
PC(D14:0-16:0)	0.75686	0.83802	0.86039	0.68887	0.83052	0.71438	0.78417	0.79867	0.50229	0.51998	0.65542	0.76134	0.7618	0.71141	0.76508	0.7084	0.85084	[illegible]
PC(D16:0-18:1)	0.68847	0.80619	0.84271	0.72505	0.86429	0.78765	0.78074	0.78676	0.59564	0.61244	0.67856	0.85108	0.81302	0.74388	0.7604	0.72972	0.87479	[illegible]
LPE(20:1)	0.54323	0.6258	0.64007	0.46338	0.61095	0.37174	0.63051	0.59022	0.21016	0.265	0.32085	0.26176	0.29785	0.45761	0.45351	0.3618	0.60153	[illegible]
PC(D18:0-20:3)	0.34143	0.47211	0.54006	0.49299	0.57238	0.50323	0.61073	0.59593	0.036338	0.0070337	0.13978	0.38375	0.22394	0.21554	0.23152	0.1509	0.47085	[illegible]
SM(N20:1)	0.44888	0.37336	0.38562	0.32778	0.43066	0.16441	0.11915	0.1127	0.64916	0.77157	0.68655	0.61558	0.66213	0.6973	0.62846	0.69725	0.53273	[illegible]
SM(N18:1)	0.72876	0.65064	0.73553	0.66652	0.86312	0.58249	0.61643	0.60046	0.63256	0.81074	0.66123	0.73764	0.76664	0.86197	0.72849	0.74357	0.73667	[illegible]
[illegible]	[illegible]	[illegible]	[illegible]	[illegible]	[illegible]	[illegible]	[illegible]	[illegible]	[illegible]	[illegible]	[illegible]	[illegible]	[illegible]	[illegible]	[illegible]	[illegible]	[illegible]	[illegible]

[illegible]	[illegible]

Supplementary Table 5. Lipid species contributing to the correlation clustersin Figure 1F and their fold changes between obese and lean EV samples.

Lipid index (as Fig. 1F)	Lipid ID	Fold change (FC) Obese vs. Lean	Cluster ID (Fig. 1F)
1	SM(N18:0)	0.554299356	a
2	SM(N24:0)	0.44223433	
3	SM(N24:1)	0.609808679	
4	LPE(P18:0)	0.736998586	
5	SM(N24:2)	0.917066849	
6	LPE(20:4)	0.871192347	
7	PC(D16:0-16:0)	0.662712158	
8	PC(D16:0-18:0)	0.605822621	
9	PS(18:0-18:1)	0.646551513	b
10	SM(N16:1)	0.838329122	
11	SM(N23:0)	0.56231182	
12	LPE(18:2)	0.553783583	
13	SM(N15:0)	0.661325326	
14	SM(N16:0)	0.625229202	
15	SM(N20:0)	0.629028872	
16	SM(N22:0)	0.556250046	
17	PC(D16:0-18:2)	0.777111258	
18	SM(N14:0)	0.687621701	
19	PA(18:0-20:4)	0.330404425	
20	PC(D18:0-18:1)	0.677527779	c
21	PC(D18:0-18:2/D18:1-18:1)	0.708118125	
22	PC(D18:1-18:2/D16:0-20:3)	0.79615758	
23	PC(D14:0-16:0)	0.614712427	
24	PC(D16:0-18:1)	0.734513873	
25	LPE(20:1)	1.010961798	d
26	PC(D18:0-20:3)	1.198740707	
27	SM(N20:1)	0.88478944	
28	SM(N18:1)	0.905623613	
29	SM(N22:1)	0.957905117	
30	SM(N23:1)	0.793042983	
31	PC(D16:1-18:2)	0.790329607	
32	LPE(18:1)	0.66454987	e
33	PC(D16:1-16:0/D14:1-18:0)	0.790781441	
34	LPE(P20:0)	0.652434105	
35	PC(D16:1-16:1/D14:1-18:1)	0.794140564	
36	PS(18:1-18:2)	1.18911163	f
37	LPC(16:0)	1.199065796	
38	LPC(18:2)	0.935228234	
39	TG(C52:0/C53:7)	0.493294675	
40	TG(C52:1/C53:8)	0.370977711	
41	FA(284(18:0))	0.602017785	
42	TG(C54:1/C55:8)	1.089824386	
43	PC(D18:0-20:2/P18:2-22:6)	1.213236454	
44	FA(254(16:1))	0.891806223	
45	TG(C48:1)	0.677241287	
46	FA(228(14:0))	0.631639881	
47	TG(C54:2/C55:9)	0.710017903	
48	TG(C52:4/C53:11)	0.886517624	
49	TG(C54:5/C55:12)	0.784536111	
50	FA(280(18:2))	0.87425785	
51	TG(C50:1/C51:8)	0.487870555	
52	FA(256(16:0))	0.691374119	
53	TG(C54:3/C55:10)	0.578409137	
54	FA(282(18:1))	0.677345692	
55	TG(C52:3/C53:10)	0.749696374	
56	TG(C54:4/C55:11)	0.72230386	
57	TG(C50:2/C51:9)	0.693316204	
58	TG(C50:3/C51:10)	0.800019438	
59	TG(C52:2/C53:9)	0.769249695	
60	LPE(16:1)	0.811260443	
61	TG(C48:0/C49:7)	0.3249048	
62	TG(C50:0/C51:7)	0.34880829	
63	LPC(18:0)	1.068501224	
64	LPE(18:0)	0.613657161	
65	TG(C53:0/C54:7)	2.216161187	
66	LPC(P16:0)	2.44449786	g
67	TG(C48:3)	0.966037217	
68	TG(C50:4)	0.933133444	
69	TG(C52:5)	1.133085829	
70	TG(C48:2)	1.077330127	
71	TG(C54:6)	1.020993168	
72	LPE(20:2)	1.729954101	h
73	LPE(P22:0)	1.289522696	
74	PA(16:0-18:1)	1.730751376	
75	PA(16:0-18:2)	1.313837004	
76	LPC(18:1)	1.838467066	
77	LPE(P16:0)	2.360750248	
78	LPE(P18:1)	2.845772839	
79	TG(C58:0/C59:7/C60:14)	1.11215969	i
80	LPE(16:0)	0.856769401	
81	TG(C54:0/C55:7)	0.680802581	
82	FA(278(18:3))	1.018990753	
83	PS(16:0-16:1)	0.617411709	
84	TG(C53:4/C54:11)	0.877507433	
85	PC(P18:1-20:1/P16:1-22:1)	1.089093216	
86	TG(C53:1/C54:8)	1.407261216	
87	TG(C55:4/C56:11)	1.630230423	
88	PC(P18:0-20:1/P16:0-22:1)	1.643940684	
89	LPC(A16:0)	2.928774794	
90	TG(C55:3/C56:10)	1.189210128	
91	TG(C53:2/C54:9)	1.572651634	
92	TG(C53:3/C54:10)	1.38954049	
93	TG(C55:2/C56:9)	1.486896329	
94	TG(C58:3/C59:10)	0.788674432	
95	PS(16:0-18:1)	0.940514249	
96	PI(16:0-18:2)	1.356300752	j
97	PI(18:0-18:2)	0.722791425	
98	TG(C59:5/C60:12)	1.125193941	
99	PC(D18:2-18:3/D16:1-20:4)	1.39303201	
100	TG(C58:1/C59:8/C60:15)	2.33130193	
101	PC(A16:0-16:0)	1.216876401	
102	PC(D18:1-22:6/D18:2-22:5)	1.286178128	
103	PC(D18:0-22:5)	1.115025288	
104	PC(D18:0-22:6)	0.915441485	
105	TG(C55:5/C56:12)	2.36005385	
106	TG(C55:6)	1.644573346	
107	TG(C58:2/C59:9)	1.980187559	
108	PC(P16:0-18:0/P18:0-16:0/A18:1-16:0/A16:0-18:1)	1.524697839	
109	PC(A18:0-18:1/P18:0-18:0)	0.37142287	
110	PC(D16:0-22:6/D18:2-20:4)	3.09328434	
111	PC(D18:1-20:4/D16:0-22:5)	2.925432827	
112	PC(P16:0-18:1/P18:1-16:0)	2.666021168	
113	PC(P16:0-18:2/A16:0-18:3)	3.176354585	
114	PC(D18:2-18:2/D16:0-20:4)	2.635395943	
115	PC(D18:2-20:2/D18:0-20:4)	2.369535644	
116	PC(P18:0-18:1/P18:1-18:0)	1.767550644	
117	PI(16:0-20:4)	0.575936244	
118	PI(18:0-20:4)	0.595573897	
119	LPC(P18:0)	3.240216307	
120	PA(18:0-18:2/18:1-18:1)	2.26323374	
121	PS(16:0-18:2)	1.343894332	
122	TG(C59:6/C60:13)	2.45949913	
123	PI(18:2-20:4/16:0-22:6)	0.644097564	
